# Assessing the relationship between coverage of essential health services and poverty levels in low- and middle-income countries

**DOI:** 10.1093/heapol/czae002

**Published:** 2024-02-01

**Authors:** Stefanny Guerra, Laurence Sj Roope, Apostolos Tsiachristas

**Affiliations:** Health Economics Research Centre, Nuffield Department of Population Health, University of Oxford, Richard Doll Building, Old Road Campus, Oxford OX3 7LF, United Kingdom; Department of Population Health Sciences, King’s College London, Guy’s Campus, Great Maze Pond, London SE1 1UL, United Kingdom; Health Economics Research Centre, Nuffield Department of Population Health, University of Oxford, Richard Doll Building, Old Road Campus, Oxford OX3 7LF, United Kingdom; Nuffield Department of Primary Care Health Sciences, University of Oxford, Radcliffe Primary Care Building, Radcliffe Observatory Quarter, Woodstock Road, Oxford OX2 6GG, United Kingdom

**Keywords:** Essential health services, global health, global poverty, poverty, Sustainable Development Goals, universal health coverage

## Abstract

Universal health coverage (UHC) aims to provide essential health services and financial protection to all. This study aimed to assess the relationship between the service coverage aspect of universal health coverage and poverty in low- and middle-income countries (LMICs). Using country-level data from 96 LMICs from 1990 to 2017, we employed fixed-effects and random-effects regressions to investigate the association of eight service coverage indicators (inpatient admissions; antenatal care; skilled birth attendance; full immunization; cervical and breast cancer screening rates; diarrhoea and acute respiratory infection treatment rates) with poverty headcount ratios and gaps at the $1.90, $3.20 and $5.50 poverty lines. Missing data were imputed using within-country linear interpolation or extrapolation. One-unit increases in seven service indicators (breast cancer screening being the only one with no significant associations) were associated with reduced poverty headcounts by 2.54, 2.46 and 1.81 percentage points at the $1.90, $3.20 and $5.50 lines, respectively. The corresponding reductions in poverty gaps were 0.99 ($1.90), 1.83 ($3.20) and 1.89 ($5.50) percentage points. Apart from cervical cancer screening, which was only significant in one poverty headcount model ($5.50 line), all other service indicators were significant in either the poverty headcount or gap models at both $1.90 and $3.20 poverty lines. In LMICs, higher service coverage rates are associated with lower incidence and intensity of poverty. Further research is warranted to identify the causal pathways and specific circumstances in which improved health services in LMICs might help to reduce poverty.

Key messages

- To our knowledge, this is the first study assessing the association between the service coverage dimensions of universal health coverage and poverty across low- and middle-income countries (LMICs).
- The study covered a wide range of LMICs over a period of 27 years and adjusted for other factors that could explain reductions in poverty, such as gross domestic product, trade, current health expenditure and political instability.
- Our findings suggest that, beyond improving population health and reducing health inequalities, policies that progress coverage of essential health services may have the additional benefit of reducing poverty.

## Introduction

Mortality and morbidity are disproportionally higher among the poor at both individual and country level (Peters *et al*., 2008; OECD, 2019). This is because factors of living in poverty, such as inadequate housing, sanitation and nutrition, are associated with infectious diseases and certain child and maternal outcomes (Gakidou *et al*., 2007). Growing evidence is also emerging on the greater burden experienced by the poor from non-communicable diseases and injuries (Bukhman *et al*., 2020). Furthermore, ill health can be a barrier to poverty alleviation, both indirectly by limiting employment opportunities and productivity and directly as single health events can be economically devastating for poor households (Cotlear *et al*., 2015). Unsurprisingly, access to quality health care has long been considered as a means to achieve better economic development and reduce health and income inequalities (WHO Commission on Macroeconomics and Health, 2001; Jamison *et al*., 2013). Universal health coverage (UHC) aims to provide health services to all people in need so that no one suffers financial hardship or health deterioration due to necessary health care expenditure. The ‘Global Health 2035’ report from the Lancet Commission on Investing in Health endorses two pro-poor approaches towards UHC (Jamison *et al*., 2013). The first focuses on providing services for events that disproportionately affect the poor, while the second advocates a wider range of health services for which the poor are exempt from any form of payment. Across low- and middle-income countries (LMICs), programmes that initially covered only poor populations have eventually expanded to include informal care workers. This illustrates how pro-poor approaches may serve as a core building block for UHC achievement (Cotlear *et al*., 2015).

UHC is considered as a key factor in achieving equity and prosperity goals and is enshrined in the Sustainable Development Goal (SDG) 3, adopted by all United Nations Member States in 2015 (UN General Assembly, 2015; Witter *et al*., 2017). As a sub-goal of SDG 3, UHC includes two indicators that monitor its progress: (1) coverage of essential health services and (2) catastrophic health expenditure, defined as out-of-pocket (OOP) health expenditures exceeding 10% or 25% of total household consumption or income (World Health Organization, World Bank, 2017). The large body of evidence on the impoverishing impact of OOP payments shows that in 2015, 926.6 million people worldwide incurred catastrophic health expenditures that exceeded 10% of their total household income or consumption (Wagstaff *et al*., 2018a,b; World Health Organization & International Bank for Reconstruction and Development, 2020). As a result, 98.8 million people were pushed below the $3.20-a-day poverty line and 9.7 million below the $1.90-a-day ‘extreme’ poverty line [2011 purchasing power parity (PPP) poverty line]. The world’s poor are mostly concentrated in LMICs (World Bank, 2022), and national studies from these countries have provided evidence that the impoverishing effects of OOP health expenditure affect poorer households to a greater extent (Limwattananon *et al*., 2011; Ataguba *et al*., 2014; Khan *et al*., 2017; Njagi *et al*., 2018; Ergo *et al*., 2019; Salari *et al*., 2019; Zhang *et al*., 2019; Kwesiga *et al*., 2020; Araujo and Coelho, 2021; Verma *et al*., 2021).

In contrast to financial risk protection, the relationship between the service coverage dimensions of UHC and poverty is under-investigated. Evidence on the relationship between service coverage indicators and poverty levels would be valuable to address this research gap.

There are plausible mechanisms via which the service coverage dimensions of UHC could impact on poverty, independently, or in the absence, of any reduction in catastrophic health expenditure. Indeed, in some instances, catastrophic health expenditures have been found to be concentrated amongst richer households (Rashad and Sharaf, 2015; Aregbeshola and Khan, 2018). In some such cases, it has been argued that this is due to poorer individuals not accessing health care in the first place, as this phenomenon is not observed in countries with better payment exceptions for the poor (Rashad and Sharaf, 2015). Similarly, in a study in Mauritius that reported increased OOP health expenditures but no impact on poverty levels (Nundoochan *et al*., 2019), this finding was partly attributed to people foregoing health care. In cases such as these, there may be important, unrecognized, impacts of UHC on poverty through its service coverage dimensions.

The literature on service coverage has mostly focused on tracking its progress (Anindya *et al*. 2021; Neal *et al*., 2015; Wagstaff *et al*., 2015; 2016; World Health Organization, World Bank, 2017; Sully *et al*., 2019; Zhang *et al*., 2019; Mwangi *et al*., 2021). In relation to poverty, reports reveal lower coverage rates for the poor on maternal (Anindya *et al*. 2021; Neal *et al*., 2015; Zhang *et al*., 2019), reproductive (Sully *et al*., 2019), new-born, child (Anindya *et al*. 2021), non-communicable disease and injury-related health services (Mwangi *et al*., 2021) across LMICs. Despite UHC progress and pro-poor efforts towards achieving UHC, it is clear that socioeconomic inequalities persist. The aim of this study was to address this evidence gap by exploring the relationship between the service coverage dimensions of UHC and poverty levels in LMICs.

## Methods

### Study design

We conducted a longitudinal retrospective observational study using country-level data from 117 LMICs. This panel data cover 27 years of observations, from 1990 to 2017, on eight UHC service coverage indicators (exposure variables), six poverty measures (outcome variables) and covariates related to economic development, health expenditure and governance (confounders).

### Data

Progress towards UHC is tracked with two SDG UHC indicators measured by different sources across WHO Member States (World Health Organization, The International Bank for Reconstruction and Development, 2022). The UHC service coverage index combines 14 tracer indicators of essential health services to monitor SDG Indicator 3.8.1 (service coverage), while financial hardship indicators monitor SDG Indicator 3.8.2 (financial protection) (World Health Organization, The International Bank for Reconstruction and Development, 2022).

As we were interested in studying the service coverage dimensions of UHC (not financial protection), we chose eight of these tracer indicators, which cover a broad range of essential health services in the categories defined by the UHC service coverage index (reproductive, maternal, new-born and child health, infectious diseases, non-communicable diseases and service capacity and access). Secondly, we chose indicators that are relatively unproblematic in terms of data availability in LMICs (Wagstaff *et al*., 2015; 2016). We obtained the following eight indicators from the WHO STEPwise Approach to NCD Risk Factor Surveillance and the Demographic and Health Surveys (DHS): rates of skilled birth attendance (SBA), antenatal care (ANC) utilization, diarrhoea and acute respiratory infection (ARI) treatment for children, full immunization of children, inpatient admissions, and screening for breast cancer (bc) and cervical cancer. These represent a percentage of service users who accessed the given health service in the past 2 weeks (e.g. diarrhoea), 12 months (e.g. inpatient) or 2 to 5 years (e.g. smear test) (Table 1). These represent reproductive, maternal and child health services, as well as infectious and non-communicable diseases. Inpatient admission may additionally serve as an indication of the wider range of services being implemented as part of UHC (Wagstaff and Neelsen, 2020).

**Table 1.** Description and source of the service coverage indicators analysed

Indicator characteristics	Indicator name	Definition	Data sources
Prevention Maternal health	ANC utilization	Percentage of most recent births in last 2 years with at least four ANC visits (women aged 15–49 years at the time of the survey)	DHS
Prevention Child health	Full immunization	Percentage of children aged 15–23 months who received Bacillus Calmette–Guérin, measles/measles–mumps–rubella, three doses of polio (excluding polio given at birth) and three doses of diphtheria–pertussis–tetanus/pentavalent vaccinations, either verified by vaccination card or by recall of respondent	DHS
Prevention Non-communicable disease	BC screening	Percentage of women who received a mammogram in the last 2 years (preferably ages 50–69 years but age groups may vary)	DHS, STEPS
Prevention Non-communicable disease	Cervical cancer screening	Percentage of women who received a pap smear in the last 5 years (preferably ages 30–49 years but age groups may vary)	DHS, STEPS
Treatment Maternal health	SBA	Percentage of most recent births in last 2 years attended by any skilled health personnel (women aged 15–49 years at the time of the survey). Definition of skilled varies by country and survey but always includes doctor, nurse, midwife and auxiliary midwife.	DHS
Treatment Child health, communicable disease	ARI treatment	Percentage of children under 5 with cough and rapid breathing (in case of data from UNICEF's Multiple Indicator Cluster Survey, originating from the chest) in the 2 weeks preceding the survey who had a consultation with a formal health care provider, excluding pharmacies and visits to other health care providers. Definition of formal health care providers varies by country and data source.	DHS
Treatment	Inpatient care	Percentage of population aged 18 years and older using inpatient care in the last 12 months	DHS

STEPS: STEPwise approach to surveillance.

Poverty headcount ratios and poverty gaps (%), both at different poverty lines: $1.90, $3.20 and $5.50 a day, expressed in 2011 US$ adjusted for PPP, were obtained from the World Development Indicators database. We also extracted data on possible confounders, including per-capita gross domestic product (GDP 2011 PPP) from the World Development Indicators database, overall current health expenditure (CHE) as a % of GDP from the WHO Global Health Expenditure Database, trade (i.e. the annual sum of exports and imports of goods and services within countries as a % of GDP) from the World Bank national accounts data, as well as control of corruption and political stability and absence of violence/terrorism (PSAV), obtained from the Worldwide Governance Indicators database. The last two governance indicators are composite measures constructed from multiple sources and presented in units of a standard normal distribution with mean zero, ranging from −2.5 to 2.5, where higher values indicate better control of corruption and PSAV (Kaufmann *et al*., 2010). All other variables are expressed in their natural units. These possible confounders were identified a priori from relevant literature. Their role and relationships with exposure and outcome variables are discussed at length and depicted in a directed acyclic graph in Supplementary Appendix II.

### Sample

We initially obtained longitudinal data on service coverage indicators and poverty measures from different datasets that collected data at various, often different, years between 1990 and 2017. This resulted in a panel data of 117 LMICs with substantial missing observations (Table 2), particularly for service coverage indicators, as data on poverty included country-year observations where no service coverage data were available. Based on the degree of missingness, we decided to match the data according to service coverage availability. This first involved replacing the outcome variable (poverty) with following year values, allowing a 1-year lag in the impact of service coverage indicators on poverty outcomes. We chose to lag these variables on the basis that improvements in service coverage may not lead to instantaneous changes in poverty. Moreover, poverty and service coverage levels are captured only on an annual basis. They are unlikely to have been measured at exactly the same time in any given country-year, and we wanted to rule out the likely possibility that we were regressing poverty variables measured early in a year $t$ on service variables measured at a later point in a year $t$. While longer lags are also plausible, we chose to lag the service indicators by only 1 year. This was mainly because, in contrast to, e.g., longer-term infrastructural investments, several of the service indicators (diarrhoea, ARI, full immunization of children, inpatient admissions) seemed likely to have a relatively fast impact on poverty, by reducing the OOP costs and lower productivity associated with infectious diseases. Whilst we could also have added additional lags, with the relatively high level of missing data, this would have reduced the sample available still further.

**Table 2.** Sample selection process and the number and percentage of missing observations in each dataset

	Initial dataset: poverty outcomes for at least 2 years, UHC data on any given year (*N* = 1315 from 117 countries)		After removing observations with no UHC data in lagged country-year (*N* = 596 from 112 countries)		Final dataset: keeping only countries with at least 2 years of poverty and lagged UHC data^b^ (*N* = 559 from 96 countries)	
Datasets	Total number of observations	Missing observations, *n* (%)	Total number of observations	Missing observations, *n* (%)	Total number of observations	Missing observations, *n* (%)
Poverty gaps and headcount ratios^a^	969	346 (26.31)	462	134 (22.48)	449	110 (19.68)
Inpatient admission	215	1100 (83.65)	215	381 (63.93)	203	356 (63.69)
SBA	389	926 (70.42)	389	207 (34.73)	368	191 (34.17)
Diarrhoea treatment	337	978 (74.37)	337	259 (43.46)	320	239 (42.75)
ARI treatment	321	994 (75.59)	321	275 (46.14)	308	251 (44.90)
BC screening	87	1228 (93.38)	87	509 (85.40)	82	477 (85.33)
Cervical cancer screening	135	1180 (89.73)	135	461 (77.35)	125	434 (77.64)
ANC utilization	335	980 (74.52)	335	261 (43.79)	320	239 (42.75)
Full immunization	364	951 (72.32)	364	232 (38.93)	346	213 (38.10)

Note: Poverty gaps and headcount ratios had same degree of missingness at all poverty lines.

a 2011 $ PPP (%).

b Dataset used in complete case analyses.

Next, country-year observations with no data on any of the eight service coverage indicators were removed (*n* = 5), reducing the panel to 112 complete cases. From these 112 countries, 16 were removed because they either had no remaining data on the outcome variable (*n* = 3) or had it for one year only (*n* = 13). This resulted in 96 LMIC complete cases with at least 2 years of data on both poverty outcomes and service coverage indicators. These 96 complete cases were included in the main analysis, which covered 27 years, and provide a sample size of 559 observations (i.e. 5.8 time points on average for each country). Details on the missing observations and the sample selection process are shown in Table 2. Descriptive statistics of the variables included in the analysis of these 96 complete cases are displayed in Table 3.

### Main regression analysis

This study consisted of an unbalanced panel dataset, covering $i$ = 1,…, 96 countries, over a period $t$ = 1,…,27 years. We first identified independent variables, which, in the literature, have been associated with both UHC service coverage indicators and poverty, in LMICs (Supplementary Appendix II). Variables identified included GDP, CHE, PSAV, trade and control of corruption. These potential confounders were then independently added to baseline models of each poverty outcome ($\gamma _{it}^{\mathrm{^{\prime}}}$) regressed on each service coverage indicator, lagged by 1 year (${\beta _1}X_{1it - 1}^{\mathrm{^{\prime}}}$) (Equation 1). The baseline fixed-effects regression was estimated as:


(1) $$\gamma _{it}^{^{\prime}} = {\beta _1}X_{1it - 1}^{^{\prime}} + \nu _i^{^{\prime}} + \varepsilon _{it}^{^{\prime}}$$

We decided to analyse the relationship between each service coverage indicator and each poverty level individually, partly to avoid issues of multicollinearity and partly because different coverage indicators are measured and collected differently, at different time points and on different populations, across individual countries. Thus, we built the final models to be tested in complete case analyses with a forward model-building strategy, where each identified confounder was added to 48 baseline models (${\gamma _{it}}$ = 6; ${X_{it - 1}}$ = 8). The number of countries analysed changed in each regression (see Table 4). As such, as each confounder was independently added to a baseline model, we calculated the Bayesian information criterion (BIC) and the Akaike information criterion (AIC) of this new model, in order to compare it to the BIC and AIC values of the previous model without the confounder. Confounders were thus independently selected based on goodness of fit using the BIC and AIC values. These forward model-building strategies resulted in different confounders being included in each regression (Supplementary Appendix II).

**Table 4.** Results from complete case analyses: the effect of service coverage indicators on poverty

	Full immunization in children^a^ (*N* countries = 85)		SBA^b^ (*N* countries = 85)		Diarrhoea treatment in children^c^ (*N* countries = 81)		ARI treatment^d^ (*N* countries = 81)	
	Coefficient (95% CI)	*P*-value	Coefficient (95% CI)	*P*-value	Coefficient (95% CI)	*P*-value	Coefficient (95% CI)	*P*-value
Poverty gap $1.90 (2011 PPP)	−0.191 (−0.323 to −0.060)^++^	0.005	−0.209 (−0.290 to −0.127)^++^	<0.001	−0.267 (−0.408 to −0.127)^++^	<0.001	−0.101 (−0.167 to −0.004)	0.040
Poverty gap $3.20 (2011 PPP)	−0.197 (−0.280 to −0.114)	<0.001	−0.259 (−0.330 to −0.187)	<0.001	−0.335 (−0.514 to −0.156)^++^	<0.001	−0.137 (−0.256 to −0.0171)	0.025
Poverty gap $5.50 (2011 PPP)	−0.209 (−0.301 to −0.117)	<0.001	−0.262 (−0.342 to −0.182)	<0.001	−0.326 (−0.573 to −0.0785)^++^	0.010	−0.122 (−0.246 to 0.003)	0.055
Poverty headcount ratio $1.90 (2011 PPP)	−0.316 (−0.524 to −0.109)^++^	0.003	−0.372 (−0.529 to −0.216)^++^	<0.001	−0.437 (−0.702 to −0.173)^++^	0.001	−0.207 (−0.378 to −0.037)	0.017
Poverty headcount ratio $3.20 (2011 PPP)	−0.268 (−0.397 to −0.139)	<0.001	−0.358 (−0.472 to −0.245)	<0.001	−0.387 (−0.768 to −0.007)^++^	0.046	−0.149 (−0.319 to 0.0201)	0.084
Poverty headcount ratio $5.50 (2011 PPP)	−0.182 (−0.292 to −0.072)	0.001	−0.195 (−0.294 to −0.0960)	<0.001	−0.244 (−0.599 to 0.110)^++^	0.175	0.019 (−0.106 to 0.145)^++^	0.755
	ANC utilization^e^ (*N* countries = 81)		Inpatient admission^f^ (*N* countries = 65)		BC screening^g^ (*N* countries = 45)		Cervical cancer screening^h^ (*N* countries = 60)	
	Coefficient (95% CI)	*P*-value	Coefficient (95% CI)	*P*-value	Coefficient (95% CI)	*P*-value	Coefficient (95% CI)	*P*-value
Poverty gap $1.90	−0.231 (−0.330 to −0.131)^++^	<0.001	−0.222 (−0.472 to 0.0285)^++^	0.082	−0.032 (−0.096 to 0.032)	0.323	−0.011 (−0.063 to 0.041)	0.677
Poverty gap $3.20	−0.314 (−0.404 to −0.224)	<0.001	−0.588 (−1.07 to −0.107)^++^	0.017	0.004 (−0.078 to 0.086)^++^	0.922	−0.045 (−0.129 to 0.039)	0.298
Poverty gap $5.50	−0.375 (−0.472 to −0.279)	<0.001	−0.716 (−1.28 to −0.156)	0.012	−0.034 (−0.172 to −0.104)	0.629	−0.097 (−0.202 to 0.075)	0.069
Poverty headcount ratio $1.90	−0.423 (−0.557 to −0.289)	<0.001	−0.790 (−1.46 to −0.123)^++^	0.021	0.008 (−0.099 to 0.115)^++^	0.875	−0.057 (−0.175 to 0.061)	0.342
Poverty headcount ratio $3.20	−0.515 (−0.651 to −0.378)	<0.001	−0.928 (−1.80 to −0.0592)	0.036	0.023 (−0.133 to 0.178)^++^	0.771	−0.138 (−0.293 to 0.168)	0.081
Poverty headcount ratio $5.50	−0.377 (−0.499 to −0.254)	<0.001	−0.871 (−1.56 to −0.181)	0.013	0.0004 (−0.190 to 0.191)	0.997	−0.184 (−0.315 to −0.054)	0.006

* All poverty gaps and headcount ratios are 2011 PPP.

Random effects unless otherwise specified.

++ Fixed-effects regression analyses.

Confounders for each UHC indicator:

a All regressions on full immunization included GDP, CHE and trade. Poverty gap at 1.90 and poverty headcount at 1.90 also included PSAV.

b All regressions on skilled birth attendance included GDP, CHE and trade.

c All regressions on diarrhoea treatment included GDP, CHE and trade. Poverty headcount at 1.90 also included PSAV.

d All regressions on ARI included GDP, CHE and trade.

e All regressions on ANC utilization included GDP, CHE and trade. Poverty gap at 1.90 and poverty headcount at 5.50 also included PSAV.

f Regressions on inpatient admissions included GDP and CHE. Poverty gap 1.90 and poverty headcount 3.20 also included PSAV, and trade poverty headcount 1.90 also included PSAV and control of corruption.

g All regressions on BC screening included GDP, CHE, trade and control of corruption. Poverty gap and headcount ratio at 1.90 only included GDP and CHE.

h All regressions on cervical cancer included GDP, CHE, PSAV and trade.

Both BIC and AIC are criteria for selection of econometric models that help to prevent overfitting. Models with lower BIC and AIC values indicate better fitting models and so are preferred. To assess the strength of the evidence given by BIC and AIC for one model against another, we calculated delta (Δ) AIC and BIC of candidate models (i.e. the model with the new added variable). Delta is the difference between the new candidate model and the previous model (also known as the ‘best’ model). If ΔAIC or ΔBIC is <2, there is evidence for the candidate model over the best model (Burnham and Anderson, 2004). Here, the candidate model was chosen if there was support from both AIC and BIC (i.e. if both resulted in Δ below 2). Since BIC is more conservative than AIC (as it more strongly penalizes the model for the number of parameters included), candidate models were not chosen if ΔAIC indicated that the candidate model was a better fit but ΔBIC did not. The final model of each fixed-effects regression was estimated as:


(2) $$\gamma _{it}^{\prime} = {\beta _1}X_{1it - 1}^{\prime} + {\beta _2}X_{2it - 1}^{\prime} + \nu _i^{\prime} + \varepsilon _{it}^{\prime}$$

All are time demeaned variables that represent a value for country $i$ at time $t$. ${\gamma _{it}}$ denotes a poverty gap or headcount ratio at the $1.90, $3.20 or $5.50 poverty line. ${X_{1it - 1}}$ represents each of the eight lagged service coverage indicators, while ${X_{2it - 1}}{\ }$are the set of lagged confounders based on AIC and BIC values. ${v_i}{\ }$ denotes unobserved country-specific effects invariant over time that may be correlated with the independent variables, and ${\varepsilon _{it}}$ is the error term.

We conducted fixed- and random-effects regression analyses on all final models (48 in total based on Equation 2) and employed the Hausman specification test to identify the most appropriate technique for each final model (Supplementary Appendix III). In panel analyses, the Hausman test can be used to differentiate between random- and fixed-effects models by testing the null hypothesis that individual-specific effects are uncorrelated with the independent variables. Therefore, under the null hypothesis, random-effects models are more efficient and preferred. Under the alternative hypothesis, the random-effects estimator is inconsistent, and so the fixed-effects estimator is to be preferred. We rejected the null hypothesis and preferred a fixed-effects model if *P* < 0.05. After the structure and type of each regression model was selected, we performed complete case analyses to test the relationship of each service coverage indicator with each poverty gap and headcount ratio.

### Sensitivity analyses

We performed two sensitivity analyses to test the robustness of the results. First, we replaced missing values of the initial dataset using interpolation and extrapolation techniques. To do this, we calculated the mean annual growth rate (AGR) of all poverty and service coverage indicators within each country and replaced the missing observations with the nearest observation adjusted for its respective AGR. We then specified the regression models following the same approach as in the main analysis. Second, given that the structure of the regression models was chosen using fixed-effects, we performed fixed-effects versions of all the models where random-effects regressions had been used to test the consistency of the complete case analysis findings regardless of the results of the Hausman test.

## Results

### Relationship between service coverage indicators and poverty measurements

**Figure 1.**
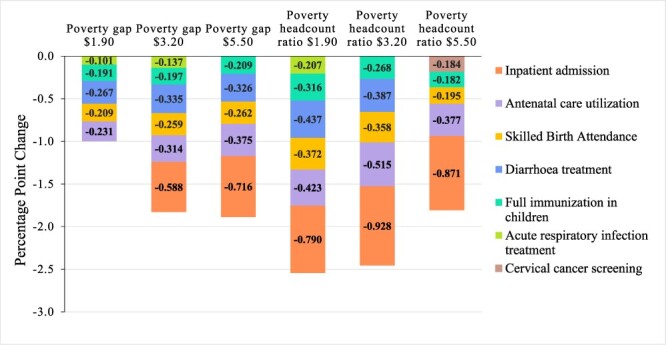
Aggregated results of main regression analyses: service coverage indicators’ associations with poverty

Note: Final models for each poverty measure on each UHC indicator were based on a forward inclusion model strategy that guided the selection of confounders included in each regression; thus, the confounder variables included in each regression vary. (See Supplementary Appendix II for a detailed description of these processes and of the confounders included in each model).

The results of the main regression analyses and the aggregated findings are shown in Table 4 and Figure 1, respectively. A detailed description of the 48 final models obtained for the regression of each service coverage indicator on each poverty measure can be found in Supplementary Appendix II. The Hausman tests indicating the preferred model, fixed- or random- effects, are provided in Supplementary Appendix III. Detailed results of sensitivity analyses are provided in Supplementary Appendix V.

Most regressions controlled for GDP, CHE and trade. PSAV was additionally included in all cervical cancer screening models; in the poverty gap at $1.90-per-day regressions on inpatient admissions, full immunization rates and ANC utilization; and in the poverty headcount ratio at $1.90-per-day regressions on inpatient admissions, full immunization rates and diarrhoea treatment. Control of corruption was included in most BC screening models (except for the poverty gap at $1.90-per-day model) and in the poverty headcount ratio at $1.90-per-day model with inpatient admissions (Table 4).

The results show that all service coverage indicators were negatively associated with at least one poverty measure, except for BC screening. This figure includes only statistically significant associations (where confidence intervals did not cross the null value of 0). Notably, increased SBA, ANC and rates of full immunization in children were associated with reductions in all poverty measures, while cervical cancer screening rates were only associated with a lower poverty headcount ratio for the $5.50 line (*P* = 0.006).

In detail, a one-unit increase in inpatient admission rate was associated with a reduction of 0.79 (*P* = 0.02), 0.928 (*P* = 0.04). 0.87 (*P* = 0.01), 0.59 (*P* = 0.02) and 0.72 (*P* = 0.01) percentage points (pp) in the respective poverty headcount ratio at $1.90, $3.20 and $5.50 lines and the poverty gap at $3.20 and $5.50 lines. Similarly, a one-unit increase in SBA rate was associated with a reduction in the poverty headcount ratio at $1.90, $3.20 and $5.50 lines and the poverty gap at $1.90, $3.20 and $5.50 lines by 0.37 (*P* < 0.001), 0.36 (*P* < 0.001), 0.20 (*P* < 0.001), 0.21 (*P* < 0.001), 0.26 (*P* < 0.001) and 0.26 (*P* < 0.001) pp, respectively. A one-unit increase in rates of diarrhoea treatment in children was associated with 0.44 (*P* = 0.001), 0.39 (*P* = 0.05), 0.27 (*P* < 0.001), 0.34 (*P* < 0.001) and 0.33 (*P* = 0.001) lower pp of poverty headcount ratio at $1.90 and $3.20 lines and poverty gap at $1.90, $3.20 and $5.50 lines, respectively. ARI treatment for children had negative and statistically significant associations with the poverty headcount ratio at the $1.90 line (−0.21, *P* = 0.02) and with the poverty gap at the $1.90 line (−0.10; *P* = 0.04) and $3.20 line (−0.14, *P* = 0.03). Moreover, a one-unit increase in the ANC rate was associated with a reduced poverty headcount ratio at the $1.90, $3.20 and $5.50 lines and a reduced poverty gap at the $1.90, $3.20 and $5.50 lines by 0.42 (*P* < 0.01), 0.52 (*P* < 0.001), 0.38 (*P* = 0.05), 0.23 (*P* < 0.001), 0.31 (*P* < 0.001) and 0.38 (*P* < 0.001), respectively. A one-unit increase in the full immunization rate was associated with a 0.32 (*P* = 0.003), 0.27 (*P* < 0.001), 0.18 (*P* = 0.001), 0.19 (*P* = 0.005), 0.20 (*P* < 0.001) and 0.21 (*P* < 0.001) pp reduction in the poverty headcount ratio at the $1.90, $3.20 and $5.50 lines and the poverty gap at the $1.90, $3.20 and $5.50 lines, respectively.

**Figure 2.**
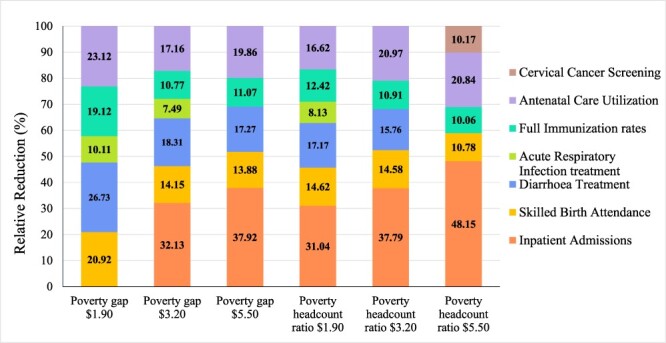
Aggregated results of main analyses: relative association of service coverage indicators with poverty

Note: Final models for each poverty measure on each UHC indicator were based on a forward inclusion model strategy that guided the selection of confounders included in each regression; thus, the confounder variables included in each regression vary. (See Supplementary Appendix II for a detailed description of these processes and of the confounders included in each model).

Figure 2 shows the relative impact of service coverage indicators on poverty measures (for statistically significant relationships only). Inpatient admissions were associated with the greatest relative reductions on the different poverty measures, except for the extreme poverty gap. For this poverty measure, a greater association was observed with diarrhoea treatment, ANC and SBA, with relative reductions of 27%, 23% and 21%, respectively. Similar relative reductions in the poverty gap at the $3.20 line and the headcount ratio at the $1.90 line were associated with inpatient admissions (32% and 31%, respectively), diarrhoea treatment (18%, 17%), ANC (both 17%), SBA (14%, 15%), full immunization (11% and 12%) and ARI treatment (8% for both). Inpatient admissions were associated with the greatest relative reduction (38%) of the poverty gap at the $5.50 line, followed by ANC (20%), diarrhoea treatment (18%), SBA (14%) and full immunization (11%). The greatest relative reductions in the headcount ratio at the $5.50 poverty line were associated with impatient admissions (48%), followed by ANC (21%), SBA (11%), full immunization (10%) and cervical cancer screening (10%).

### Robustness of results

**Figure 3.**
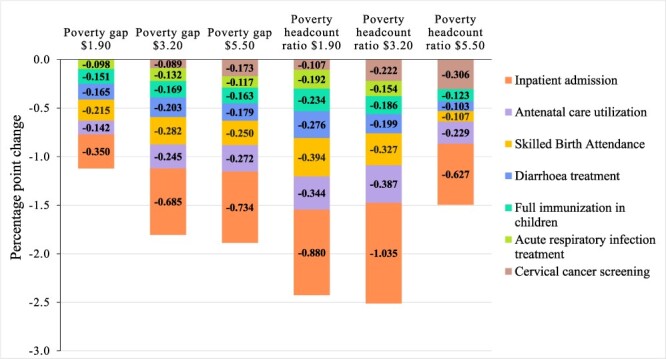
Aggregated results of sensitivity analyses: service coverage indicators’ associations with poverty in the imputed sample

**Figure 4.**
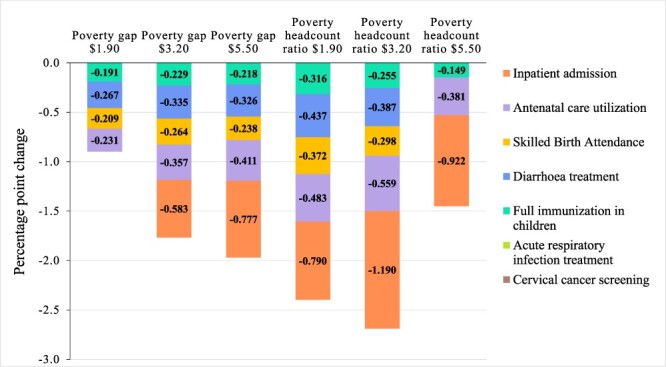
Aggregated results of sensitivity analysis: service coverage indicators’ associations with poverty, using only fixed-effects regressions

The results from the regression analysis on the imputed dataset were similar to the results of the main analysis for inpatient admission, full immunization, diarrhoea treatment, SBA, ANC utilization and BC screening rates (see Figure 3). However, the results of the imputed dataset also indicated that inpatient admissions and diarrhoea were associated with a reduced poverty gap at the $1.90 line and a reduced headcount ratio at the $5.50 line—the only poverty measures not related to these indicators in the main analyses. The ARI treatment and the cervical cancer screening rate coefficients became negative and statistically significant, indicating an association with lower poverty for most poverty measures, except for the poverty headcount ratio at the $5.50 line and the poverty gap at the $1.90 line. Associations were overall smaller, and the ones that showed a statistically significant relationship in the main analyses were maintained in sensitivity analyses. When looking at the results of only fixed-effects regressions (Figure 4), the magnitude of coefficients, the direction of relationships and the *P*-values were similar to those of the main analysis, with three notable exceptions. The associations between ARI treatment rates and cervical cancer screening rates and poverty were no longer statistically significant for any poverty measure and neither was the association between SBA rate and the poverty headcount at the $5.50 line.

## Discussion

We sought to understand the association between increased health service coverage and poverty in LMICs. Depending on the health service indicator and the poverty variable analysed, we found that service coverage was either associated with reduced poverty or had no significant relationship with poverty (in either direction). Overall, the results illustrate that higher coverage rates of inpatient, maternal and child health services are associated with smaller poverty gaps and fewer people living below the $1.90, $3.20 and $5.50-per-day poverty lines, when controlling for key confounders, such as GDP per capita, CHE and trade. These associations were maintained for most associations in sensitivity analyses.

Most service coverage indicators had greatest associations with the poverty headcount ratio at the $1.90 and $3.20 lines, followed by the poverty gap at the $5.50 and $3.20 lines and least association with the poverty headcount ratio at the $5.50 line and the poverty gap at the $1.90 line. All indicators, except cervical cancer screening, followed this pattern, with slight variations. Overall, these results appear to suggest that these services have the greatest association with poverty reduction for people below the lower poverty lines. However, an important caveat is the finding that the association with the poverty gap at the $1.90 line is small. This together with the strong association with the headcount at the $1.90 line might suggest that these services are good at helping people a little below the $1.90 line but not good at helping people far below this (already very low) line. However, it is not immediately clear what might explain this.

Inpatient admission was associated with the greatest reduction across most poverty variables, although it also had one of the lowest median coverage rates (Table 3). Notoriously high and unpredictable costs of hospitalizations may contribute to delaying or avoiding inpatient care altogether, especially if not covered. In Bangladesh, e.g., insured poor people show higher hospitalization rates than non-covered poor individuals, despite similar duration of stay and OOP health expenditures (Ahmed *et al*., 2021). Moreover, it may be that increasing low covered indicators is most beneficial for poorer people, who in such situations may access services to an even lesser extent. For example, the literature on maternal service coverage has shown lower coverage rates for poorer women; a gap that increases as coverage decreases, with the most profound inequalities observed among poorer women in countries with the lowest coverage rates (Neal *et al*., 2015). It may be that the effects of increasing service coverage are exponential rather than linear, particularly for under accessed or not covered services, such as inpatient admissions and maternal health services.

**Table 3.** Descriptive statistics of all variables used in complete case analysis, expressed as medians (IQR) and means (SD)

Variables	Sample size	Median (IQR)	Min.–Max.	Variables	Sample size	Median (IQR)	Min.–Max.
Poverty gap $1.90 (2011 PPP) (%)	449	3.5 (12.1)	0.00–64.1	Inpatient admissions^a^	203	6.27 (3.05)	0.53–14.8
Poverty gap $3.20 (2011 PPP) (%)	449	11.9 (26.4)	0.00–77.4	SBA	368	68.7 (44.2)	5.76–100
Poverty gap $5.50 (2011 PPP) (%)	449	28.2 (38.8)	0.00–86.6	Diarrhoea treatment^a^	320	36.8 (17.0)	5.88–85.5
Poverty headcount ratio $1.90 (2011 PPP) (%)	449	12.8 (34.9)	0.00–94.3	ARI treatment^a^	308	51.2 (18.0)	6.32–94.87
Poverty headcount ratio $3.20 (2011 PPP) (%)	449	34.6 (56.7)	0.00–98.5	BC screening	82	6.53 (14.8)	0.08–67.4
Poverty headcount ratio $5.50 (2011 PPP) (%)	449	64.6 (50.8)	0.80–99.7	Cervical cancer screening	125	17.7 (52.3)	0.16–87.9
GDP per capita (2011 $ PPP)	544	4387.8 (6360.8)	495.4–25 551.1	ANC utilization	320	62.1 (37.7)	6.17–99.7
Control of corruption^a^	514	−0.60 (0.46)	−1.66 to 1.57	Full immunization rates	346	64.1 (27.8)	10.4–97.2
PSAV^a^	513	−0.56 (0.73)	−2.64 to 1.12	CHE (% of GDP)^a^	461	5.53 (2.21)	1.06–16.6
Trade (% of GDP)^a^	546	70.5 (33.4)	0.167–277.1				

Note: All poverty gaps and headcount ratios are lagged.

a Expressed as mean (standard deviation).

IQR: interquartile range.

Our findings show that reductions in poverty are associated with increased coverage rates of maternal and child health services. Previous research has reported inequalities across LMICs for poorer households and those living in rural areas for these services (Anindya *et al*. 2021; Khan *et al*., 2017; Joseph *et al*., 2018; Sully *et al*., 2019). It is thus likely that maternal and child health services are particularly relevant, useful and accessed (when covered) for the poorest families in LMICs. For instance, a longitudinal study in Ghana found that the impact of immunization on child survival is greater for the poorest children, even in a context where almost all are poor (Bawah *et al*., 2010). In another Bangladesh study, catastrophic health expenditure at the 10% level occurred in >46% of families seeking diarrhoea treatment for children, and this was highest among those in the poorest quintile (Hasan *et al*., 2021).

It is worth noting that the statistically significant associations for increased ARI treatment had relatively the weakest association with reduced poverty in both complete case and sensitivity analyses and was only found when random-effect models were employed. If effect sizes are very small, fixed-effects regressions are often less able than random-effects regressions to detect them, which may explain the differences between these models. However, it is sometimes difficult to argue that the individual-specific effects are uncorrelated with the independent variables, and therefore, the fixed-effects are preferred over the random-effects models (Wooldridge, 2012).

The non-communicable disease indicators (breast and cervical cancer screening) were generally not associated with poverty, the only statistically significant association being between cervical cancer screening rates and the poverty headcount ratio at $5.50. This may be because, as with child health, preventative measures may take longer periods of time to have an effect on poverty. In addition, the degree of missingness was greater among non-communicable disease indicators (Table 2). Prevention for non-communicable diseases may only recently have been implemented in many LMICs, with low uptakes and strategies indicative of a previous major focus on infectious diseases. It remains crucial to improve data collection for non-communicable diseases indicators, particularly in LMICs where it is most limited.

The results of the imputed dataset (sensitivity) analysis on cervical cancer screening showed significant negative relationships with all poverty measures, except with the extreme poverty gap. Whilst data availability for these two indicators is the main challenge of interpretation, it is worth noting that the pattern observed for increased cancer screening rates in the imputed dataset follows the reverse pattern of the other indicators. This may suggest that, contrary to the other indicators, cervical cancer screening may provide most benefit to the least poor. This is relevant because most service coverage indicators explored had a weaker association, or no association, with the headcount ratio at the highest poverty line.

### Implications

Coronavirus disease 2019 (COVID-19) has weakened and disrupted health systems globally, with communicable disease services particularly affected (Kickbusch and Gitahi, 2020). Estimates suggest that if it had not been for COVID-19, ∼31 million people would have otherwise escaped extreme poverty in 2020. In the same year, the pandemic pushed 97 million people into poverty (Gerszon Mahler *et al*., 2021). While poverty continued to decline in many high- and upper-middle-income countries in 2021, low-income countries and countries in Sub-Saharan Africa are expected to continue to see increases (Gerszon Mahler *et al*., 2021).

As decades of steady progress on reducing extreme poverty have come to an end and the goal of eradicating it by 2030 is deemed unlikely (Slotman, 2020), policymakers have to envisage what the strategies for poverty alleviation and reduction will include. It has been suggested that these should both reduce inequality and promote development (Kickbusch and Gitahi, 2020). UHC service coverage is potentially one relevant option. However, even before the pandemic, UHC service coverage had weakened globally since 2010, especially in lower-income countries.

### Strengths and limitations

To our knowledge, this is the first study assessing the association of the service coverage dimensions of UHC with poverty across LMICs. The study covered a wide range of LMICs over a period of 27 years and included indicators of child, inpatient, maternal and preventative services. We also controlled for key confounders that could have explained reductions in poverty and increased access to UHC. These included country-level factors related to overall economic development (GDP per capita and trade), governance measures (PSAV and control of corruption) and health investments (CHE). However, missing data remain a central challenge of this study, similar to the overall literature on UHC progress, particularly for non-communicable diseases and in LMICs. As such, in this study, we used lagged health care service variables and imputation techniques, with results being broadly similar in sensitivity analyses. Fixed- and random-effects regressions adjust for any unobserved heterogeneity that is time invariant. However, though we adjusted for a number of time-varying confounders proposed in the literature (Supplementary Appendix I), we cannot rule out the possibility that our results could be affected by unobserved time-varying heterogeneity, meaning that the associations described in this study may not necessarily be causal. Another limitation is that the dataset did not permit us to adjust for or disaggregate according to education, urban/rural populations or regions. This is an area that has only recently been reported for UHC indicators (World Health Organization, World Bank, 2017) and would be an important avenue for future research. An additional area for future research may include finding ways to adequately investigate the possible long-run impact of UHC indicators, which may provide further evidence on the impact of child and preventative health. Finally, as in all studies where statistical inference is employed after model selection, *P*-values and statistical significance must be treated with caution and our analysis must be regarded as exploratory (Shen *et al*., 2004).

## Conclusions

Increasing coverage of maternal, child and inpatient services is associated with reduced poverty in LMICs. Our findings support pro-poor approaches towards UHC, with the notion that providing health services known to affect the poor may help to prevent and reduce poverty. At a time when the long decline in global poverty levels has unfortunately reversed, these findings suggest that policies that increase coverage of essential health services might also contribute to reductions in poverty levels. Further research is needed to replicate our results and to identify the causal pathways and specific circumstances in which improved health services in LMICs might help to reduce poverty.

## Supplementary Material

czae002_Supp

## Data Availability

The data that support the findings of this study are available from the corresponding author upon reasonable request. The data that support the findings of this study are publicly available from the following resources: The DHS Program—Available Datasets, World Development Indicators | DataBank (worldbank.org), Global Health Expenditure Database (who.int), WGI 2021 Interactive > Home (worldbank.org), STEPwise Approach to NCD Risk Factor Surveillance (STEPS) (who.int).

## Reflexivity statement

The authors include one female and two males and span varying levels of seniority from research assistant to associate professor. The first author (S.G.) has a background in medicine and population health, with a special focus on Latin America, where she is originally from. The second author (L.S.J.R.) has extensive expertise across a range of topics in development economics and health economics, with a particular focus on measurement of poverty and inequalities at a global level. The third author (A.T.) is an expert in the economic evaluation of complex health interventions and leads programmes of research that focus on the economic evaluation of new models of health care delivery and organization. All three authors have extensive experience conducting quantitative research focused on LMICs.

## References

[R1] Ahmed S, Ahmed MW, Hasan MZ et al. 2021. Assessing the incidence of catastrophic health expenditure and impoverishment from out-of-pocket payments and their determinants in Bangladesh: evidence from the nationwide household income and expenditure survey 2016. *International Health* 14: 84–96. 10.1093/inthealth/ihab015 PMC8769950 33823538

[R2] Anindya K, Marthias T, Vellakkal S et al. 2021. Socioeconomic inequalities in effective service coverage for reproductive, maternal, newborn, and child health: a comparative analysis of 39 low-income and middle-income countries. *EClinicalMedicine* 40: 101103. 10.1016/j.eclinm.2021.101103 PMC8430373 34527893

[R3] Araujo EC, Coelho BDP. 2021. Measuring financial protection in health in Brazil: catastrophic and poverty impacts of health care payments using the latest national household consumption survey. *Health Systems & Reform* 7: e1957537. 10.1080/23288604.2021.1957537 34547982

[R4] Aregbeshola BS, Khan SM. 2018. Out-of-pocket payments, catastrophic health expenditure and poverty among households in Nigeria 2010. *International Journal of Health Policy and Management* 7: 798–806. 30316228 10.15171/ijhpm.2018.19 PMC6186489

[R5] Ataguba JE, Day C, McIntyre D. 2014. Monitoring and evaluating progress towards universal health coverage in South Africa. *PLoS Medicine* 11: e1001686. 10.1371/journal.pmed.1001686 PMC4170961 25243462

[R6] Bawah AA, Phillips JF, Adjuik M et al. 2010. The impact of immunization on the association between poverty and child survival: evidence from Kassena-Nankana District of northern Ghana. *Scandinavian Journal of Public Health* 38: 103–95. 10.1177/1403494809352532 19884162

[R7] Bukhman G, Mocumbi AO, Atun R et al. 2020. The Lancet NCDI Poverty Commission: bridging a gap in universal health coverage for the poorest billion. *The Lancet* 396: 991–1044. 10.1016/S0140-6736(20)31907-3 PMC7489932 32941823

[R8] Burnham KP, Anderson DR. 2004. Multimodel inference: understanding AIC and BIC in model selection. *Sociological Methods & Research* 33: 261–304.

[R9] Cotlear D, Nagpal S, Smith O et al. 2015. *Going Universal: How 24 Developing Countries are Implementing Universal Health Coverage from the Bottom Up*. Washington, DC: World Bank.

[R10] Ergo A, Htoo TS, Badiani-Magnusson R et al. 2019. A new hope: from neglect of the health sector to aspirations for universal health coverage in Myanmar. *Health Policy & Planning* 34: i38–46. 31644797 10.1093/heapol/czy110 PMC6807514

[R11] Gakidou E, Oza S, Fuertes CV et al. 2007. Improving child survival through environmental and nutritional interventions: the importance of targeting interventions toward the poor. *Journal of the American Medical Association* 298: 1876–87. 17954539 10.1001/jama.298.16.1876

[R12] Gerszon Mahler D, Yonzan N, Lakner C et al. 2021. Updated estimates of the impact of COVID-19 on global poverty: turning the corner on the pandemic in 2021? *Data Blog, World Bank Blogs* Washington DC, United States: World Bank.

[R13] Hasan MZ, Mehdi GG, de Broucker G et al. 2021. The economic burden of diarrhea in children under 5 years in Bangladesh. *International Journal of Infectious Diseases* 107: 37–46. 33864914 10.1016/j.ijid.2021.04.038 PMC8208894

[R14] Jamison DT, Summers LH, Alleyne G et al. 2013. Global health 2035: a world converging within a generation. *The Lancet* 382: 1898–955. 10.1016/S0140-6736(13)62105-4 24309475

[R15] Joseph G, Mohnsam da Silva IC, Barros AJD et al. 2018. Socioeconomic inequalities in access to skilled birth attendance among urban and rural women in low-income and middle-income countries. *BMJ Global Health* 3: e000898. 10.1136/bmjgh-2018-000898 PMC6278921 30588340

[R16] Kaufmann D, Kraay A, Mastruzzi M. 2010. The worldwide governance indicators: methodology and analytical issues. *Hague Journal on the Rule of Law* 3: 220–46.

[R17] Khan JAM, Ahmed S, Evans TG. 2017. Catastrophic healthcare expenditure and poverty related to out-of-pocket payments for healthcare in Bangladesh—an estimation of financial risk protection of universal health coverage. *Health Policy & Planning* 32: 1102–10. 28575415 10.1093/heapol/czx048

[R18] Kickbusch I, Gitahi G. 2020. COVID-19 (coronavirus): universal health coverage in times of crisis. *Investing in Health, World Bank Blogs* Washington DC, United States: World Bank.

[R19] Kwesiga B, Aliti T, Nabukhonzo P et al. 2020. What has been the progress in addressing financial risk in Uganda? Analysis of catastrophe and impoverishment due to health payments. *BMC Health Services Research* 20: 1–8. 10.1186/s12913-020-05500-2 PMC7425531 32787844

[R20] Limwattananon S, Vongmongkol V, Prakongsai P et al. 2011. The equity impact of universal coverage: health care finance, catastrophic health expenditure, utilization and government subsidies in Thailand. *Consortium for Research on Equitable Health Systems (CREHS)*. Thailand: International Health Policy Program, Ministry of Public Health, 1–40.

[R21] Mwangi K, Gathecha G, Nyamongo M et al. 2021. Reframing non-communicable diseases and injuries for equity in the era of universal health coverage: findings and recommendations from the Kenya NCDI Poverty Commission. *Annals of Global Health* 87: 1–16. 33505862 10.5334/aogh.3085 PMC7792462

[R22] Neal S, Channon AA, Carter S et al. 2015. Universal health care and equity: evidence of maternal health based on an analysis of demographic and household survey data. *International Journal for Equity in Health* 14: 1–12. 26076751 10.1186/s12939-015-0184-9 PMC4489140

[R23] Njagi P, Arsenijevic J, Groot W. 2018. Understanding variations in catastrophic health expenditure, its underlying determinants and impoverishment in sub-Saharan African countries: a scoping review. *Systematic Reviews* 7: 1–23. 30205846 10.1186/s13643-018-0799-1 PMC6134791

[R24] Nundoochan A, Thorabally Y, Monohur S et al. 2019. Impact of out of pocket payments on financial risk protection indicators in a setting with no user fees: the case of Mauritius. *International Journal for Equity in Health* 18: 1–11. 31053077 10.1186/s12939-019-0959-5 PMC6500054

[R25] OECD . 2019. *Health for Everyone? Social Inequalities in Health and Health Systems*. Paris: OECD Publishing.

[R26] Peters DH, Garg A, Bloom G et al. 2008. Poverty and access to health care in developing countries. *Annals of the New York Academy of Sciences* 1136: 161–71. 17954679 10.1196/annals.1425.011

[R27] Rashad AS, Sharaf MF. 2015. Catastrophic economic consequences of healthcare payments: effects on poverty estimates in Egypt, Jordan, and Palestine. *Economies* 3: 216–34.

[R30] Salari P, Di Giorgio L, Ilinca S et al. 2019. The catastrophic and impoverishing effects of out-of-pocket healthcare payments in Kenya, 2018. *BMJ Global Health* 4: e001809. 10.1136/bmjgh-2019-001809 PMC6882550 31803510

[R31] Shen X, Huang HC, Ye J. 2004. Inference after model selection. *Journal of the American Statistical Association* 99: 751–62.

[R28] Slotman JR . 2020. UN/DESA Policy Brief #86: the long-term impact of COVID-19 on poverty.

[R32] Sully EA, Biddlecom AS, Darroch JE. 2019. Not all inequalities are equal: differences in coverage across the continuum of reproductive health services. *BMJ Global Health* 4: e001695. 10.1136/bmjgh-2019-001695 PMC6730583 31544002

[R33] UN General Assembly . 2015. A/RES/70/1—Transforming our world: the 2030 Agenda for Sustainable Development. *Sustainable Development Knowledge Platform*.

[R34] Verma VR, Kumar P, Dash U. 2021. Assessing the household economic burden of non-communicable diseases in India: evidence from repeated cross-sectional surveys. *BMC Public Health* 21: 1–22. 33962625 10.1186/s12889-021-10828-3 PMC8106177

[R35] Wagstaff A, Cotlear D, Eozenou PHV et al. 2016. Measuring progress towards universal health coverage: with an application to 24 developing countries. Washington, DC: Oxford Academic.

[R36] Wagstaff A, Dmytraczenko T, Almeida G et al. 2015. Assessing Latin America’s progress toward achieving universal health coverage. *Health Affairs* 34: 1704–12. 26438747 10.1377/hlthaff.2014.1453

[R37] Wagstaff A, Flores G, Hsu J et al. 2018a. Progress on catastrophic health spending in 133 countries: a retrospective observational study. *The Lancet Global Health* 6: e169–79. 29248367 10.1016/S2214-109X(17)30429-1

[R38] Wagstaff A, Flores G, Smitz MF et al. 2018b. Progress on impoverishing health spending in 122 countries: a retrospective observational study. *The Lancet Global Health* 6: e180–92. 29248366 10.1016/S2214-109X(17)30486-2

[R39] Wagstaff A, Neelsen S. 2020. A comprehensive assessment of universal health coverage in 111 countries: a retrospective observational study. *The Lancet Global Health* 8: e39–49. 31837954 10.1016/S2214-109X(19)30463-2

[R40] WHO Commission on Macroeconomics and Health . 2001. Macroeconomics and health: investing in health for economic development. Executive summary/report of the Commission on Macroeconomics and Health. Geneva, Switzerland: World Health Organization.

[R41] Witter S, Govender V, Ravindran TS et al. 2017. Minding the gaps: health financing, universal health coverage and gender. *Health Policy & Planning* 32: v4–v12. 28973503 10.1093/heapol/czx063 PMC5886176

[R42] Wooldridge J . 2012. *Introductory Econometrics: A Modern Approach*, 5th edn. Washington, DC: South-Western College Pub.

[R29] World Bank . 2022. *Total of population living in extreme poverty by world region* World Bank Poverty and Inequality Platform (with major processing by Our World in Data), Washington Dnited States.

[R43] World Health Organization & International Bank for Reconstruction and Development . 2020. Global monitoring report on financial protection in health 2019. Geneva, Switzerland.

[R44] World Health Organization, The International Bank for Reconstruction and Development . 2022. Tracking universal health coverage: 2021 global monitoring report. Geneva, Switzerland

[R45] World Health Organization, World Bank . 2017. Tracking universal health coverage: 2017 global monitoring report. Washington DC, United States: World Bank Group.

[R46] Zhang C, Shafiur Rahman MD, Mizanur Rahman MD et al. 2019. Trends and projections of universal health coverage indicators in Ghana, 1995-2030: a national and subnational study. *PLoS One* 14: e0209126. 10.1371/journal.pone.0209126 PMC6530887 31116754

