# Supplementary material for: Assessing the relationship between coverage of essential health services and poverty levels in low- and middle-income countries
Source: Health Policy Plan. 2024 Feb 1;39(2):156–67. doi: 10.1093/heapol/czae002 (PMC10883664; doi:10.1093/heapol/czae002)
Supplement: czae002_Supp [file czae002_supp.zip › suppl_data/Appendix I - Literature covariates amended.docx]

# **Appendix I – Literature on variables associated with UHC and poverty**

A review of previous literature and factors that affect both exposure and outcome was conducted to identify and control for key confounders. Figure A1 summarises the directed acyclic graph developed to depict these relationships.

A variable usually accounted for in cross-country data analyses of health and impoverishment is GDP (Adams 2003; Doorslaer *et al.* 2006; Wagstaff *et al.* 2018a, 2018b). GDP measures the total production of a country, serving as an indicator of the size of an economy and its development. As such, GDP tends to be negatively associated with poverty in developing countries (Dollar and Kraay 2002; Xu *et al.* 2007; Dollar, Kleineberg and Kraay 2016). GDP is also associated with the service coverage indicators used in this study (except for inpatient admissions), showing significant positive associations across countries (Wagstaff and Neelsen 2020) Further, evidence from a longitudinal study of 194 countries suggest that for countries to start acting on legislation towards UHC, a minimum GDP per capita of $5000 (2005 PPP) is needed (Feigl and Ding 2013).

Current health expenditure (CHE) indicates various financing schemes by which individual countries may advance UHC. WHO’s latest recommendations encourage countries to review their CHE and increase it as necessary to achieve UHC. These also emphasise its target for all nations to increase public health spending by at least an additional 1% of GDP, a goal believed to be achievable by all countries (World Health Organization 2019). Other analyses recommend LMIC to set CHE targets to at least 5% of GDP in order to progress towards UHC(McIntyre, Meheus and Rottingen 2017). Moreover, CHE has been significantly positively associated with the eight service coverage indicators included in this study, and negatively associated with catastrophic health expenditure (Wagstaff and Neelsen, 2020). Numerous health financing schemes can make up CHE in a given country, such as government, non-profit, voluntary, social health insurance (SHI) and compulsory schemes, though only SHI and government schemes have shown positive significant associations with service coverage indicators (Wagstaff and Neelsen, 2020).

Poverty follows a similar pattern, whereby SHI and government schemes are significantly negatively associated with incidence of impoverishment due to out-of-pocket (OOP) health expenditure, while total health expenditure is positively correlated with OOP impoverishment at a relative poverty line (50% of median consumption) and at the $3.10-per-day poverty line (Wagstaff *et al.* 2018b). Growing bodies of research have further evidenced impoverishment as a consequence of OOP health expenditure, particularly in LMIC and among poorer households (Wagstaff and van Doorslaer 2003; McIntyre *et al.* 2006; Arsenijevic, Pavlova and Groot 2013; van Minh *et al.* 2013; Hamid, Ahsan and Begum 2014; Rashad and Sharaf 2015; Koch, Cid Pedraza and Schmid 2017). Further, impoverishment due to OOP health expenditure has been observed for countries at all income levels (Wagstaff *et al.* 2018b).

We know implementing UHC is a political decision. We aimed to capture this by including estimates of governance effectiveness, namely political stability and lack of terrorism (PSAV), and control of corruption. There are numerous ways in which corruption can affect UHC attainment and health services delivery. For example, corruption impedes reforms to health systems that may otherwise pave the way for UHC. It limits access and affects the quality, equity, responsiveness, outcomes, and efficiency of health services (Vian 2008; García 2019). Furthermore, corruption leads to overall weakening of public services. Since the poor are less likely to seek private services, they suffer the consequences of corrupt systems to a greater extent. This disproportionate and detrimental effect has been investigated in LMIC, showing associations of corruption with higher income inequalities, and poorer health and education(Gupta, Davoodi and Alonso-Terme 2002; Chetwynd, Chetwynd and Spector 2003; Rahayu and Widodo 2012; Doumbia 2019; Eshun and Baah 2020)In South Africa, corruption has been found to intensify poverty despite globalisation efforts that otherwise may have had the opposite effect (Salahuddin *et al.* 2019).

In addition, economic development may be a challenge when nations deal with political instability and terrorism, as these are associated with negative risk perceptions from potential investors, lower growth rates of GDP per capita, productivity, and human capital accumulation (Nel 2003; Aisen and Veiga 2013; Shabbir, Anwar and Adil Shahid 2016; Shumetie and Watabaji 2019). Political instability and terrorism are also more likely to occur in poorer countries; within these, related events are more likely to occur in areas where the poor live and impact sectors that they rely on, such as tourism (Nel 2003; The Borgen Project 2018). At the same time, political- related instability and violence may weaken basic public services and decrease public funding, thus limiting the resources available for the poor (Nel 2003; Ndubai *et al.* 2017; The Borgen Project 2018). In line with this, democracy has been identified as an important predictor of countries likelihood of achieving UHC, as it impacts their ability to create UHC laws and expand coverage and access to essential health services (Feigl and Ding 2013).

Lastly, although scarce evidence is available, the extent and nature of international trade may facilitate decisions towards UHC, particularly for LMIC that may not have the infrastructure, access to technology, and other resources to increase and deliver essential health care services. Trade agreements that would benefit UHC may not be popular with the public and may contradict other economic goals that take priority. Hence, trade could advance and also impede UHC (Fukuda-Parr and Treanor 2018).

Trade is usually seen as a way of alleviating poverty and, at a national level, to boost economic growth. However, there is ambiguous theoretical and empirical evidence on the effect of trade on poverty, with studies finding associations with increased, reduced, and null effects(Fukuda-Parr and Treanor 2018). It is likely that trade’s impact on poverty depends on the stage of economic development, the type of agreements reached, the time evaluated (most studies find a positive correlation in the long-term) and country-specific characteristics such as corruption and government effectiveness (Salahuddin *et al.* 2019).

**Supplementary Figure A1. Depiction of pathways via which service coverage might impact on poverty**


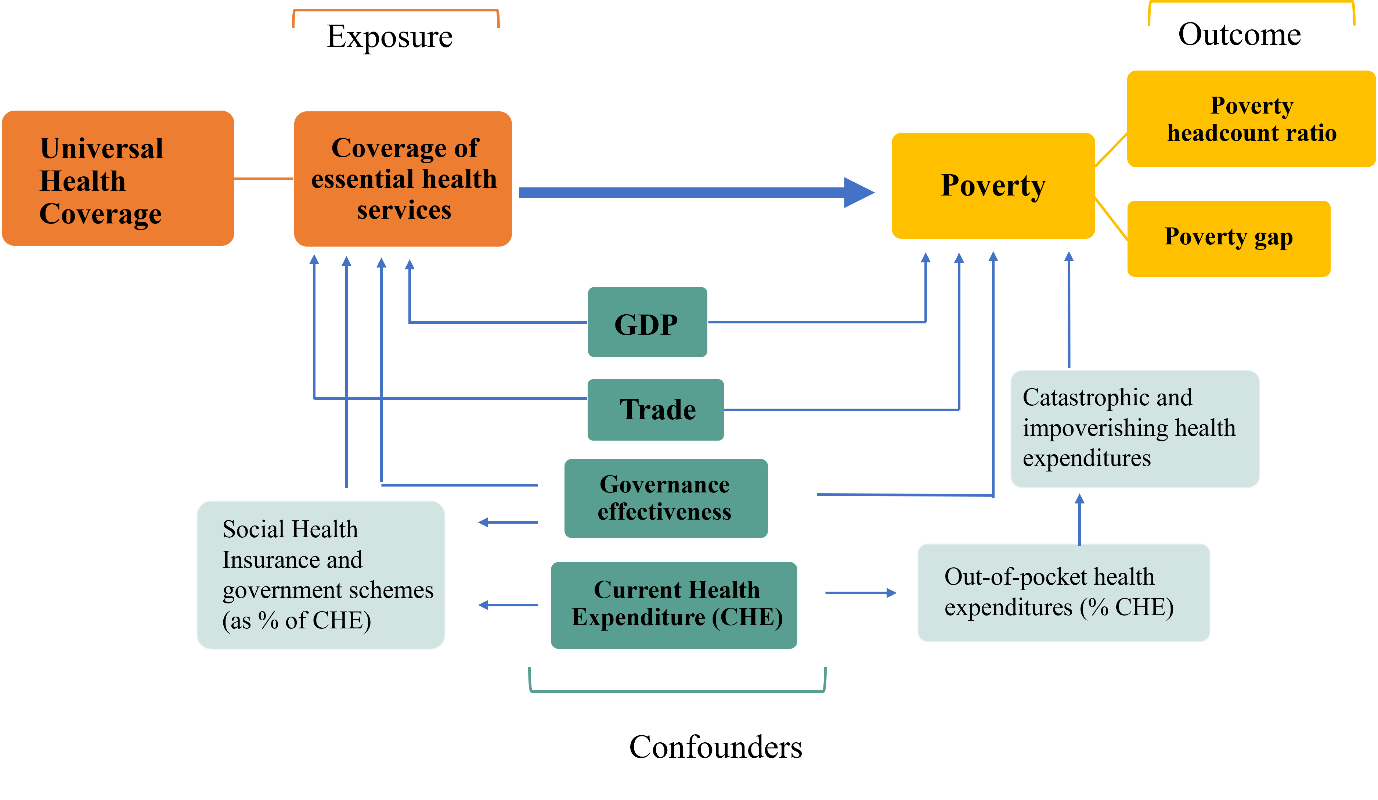


**References**

Adams RJr. *Economic Growth, İnequality, and Poverty: Findings from a New Data Set*. The World Bank, 2003.

Aisen A, Veiga FJ. How does political instability affect economic growth? *Eur J Polit Econ* 2013;**29**:151–67.

Arsenijevic J, Pavlova M, Groot W. Measuring the catastrophic and impoverishing effect of household health care spending in Serbia. *Soc Sci Med* 2013;**78**:17–25.

Chetwynd E, Chetwynd F, Spector B. *Corruption and Poverty: A Review of Recent Literature*., 2003.

Dollar D, Kleineberg T, Kraay A. Growth still is good for the poor. *Eur Econ Rev* 2016;**81**:68–85.

Dollar D, Kraay A. Growth is Good for the Poor. *Journal of Economic Growth 2002 7:3* 2002;**7**:195–225.

Doorslaer E van, O’Donnell O, Rannan-Eliya RP *et al.* Effect of payments for health care on poverty estimates in 11 countries in Asia: an analysis of household survey data. *The Lancet* 2006;**368**:1357–64.

Doumbia D. The quest for pro-poor and inclusive growth: the role of governance. *Appl Econ* 2019;**51**:1762–83.

Eshun J, Baah JA. What Does Recent Survey Data Say About the Effect of Corruption on Poverty in Africa? *Journal of Economics, Management and Trade* 2020:1–13.

Feigl AB, Ding EL. Evidenced formal coverage index and universal healthcare enactment: A prospective longitudinal study of economic, social, and political predictors of 194 countries. *Health Policy (New York)* 2013;**113**:50–60.

Fukuda-Parr S, Treanor K. *Trade Agreements and Policy Space for Achieving Universal Health Coverage (SDG Target 3.8)*. United Nations, Department of Economics and Social Affairs, 2018.

García PJ. Corruption in global health: the open secret. *The Lancet* 2019;**394**:2119–24.

Gupta S, Davoodi H, Alonso-Terme R. Does corruption affect income inequality and poverty? *Economics of Governance* 2002;**3**:23–45.

Hamid SA, Ahsan SM, Begum A. Disease-specific impoverishment impact of out-of-pocket payments for health care: evidence from rural Bangladesh. *Appl Health Econ Health Policy* 2014;**12**:421–33.

Koch KJ, Cid Pedraza C, Schmid A. Out-of-pocket expenditure and financial protection in the Chilean health care system-A systematic review. *Health Policy* 2017;**121**:481–94.

McIntyre D, Meheus F, Rottingen JA. What level of domestic government health expenditure should we aspire to for universal health coverage? *Health Econ Policy Law* 2017;**12**:125–37.

McIntyre D, Thiede M, Dahlgren G *et al.* What are the economic consequences for households of illness and of paying for health care in low- and middle-income country contexts? *Soc Sci Med* 2006;**62**:858–65.

van Minh H, Kim Phuong NT, Saksena P *et al.* Financial burden of household out-of pocket health expenditure in Viet Nam: findings from the National Living Standard Survey 2002-2010. *Soc Sci Med* 2013;**96**:258–63.

Ndubai RE, Mbeche IM, Pokhariyal GP *et al.* A Study of the Intervening Effect of Political Stability on the Relationship between Performance Contracting and Measurement, and Public Service Delivery in Kenya. *Open Access Library Journal* 2017;**4**:1–14.

Nel P. Income Inequality, Economic Growth, and Political Instability in Sub-Saharan Africa. *Journal of Modern African Studies* 2003;**41**:611–39.

Rahayu IP, Widodo T. The Causal Relationship between Corruption and Poverty in ASEAN: a General Method of Moments/Dynamic Panel Data Analysis. *Journal of Economics, Business, and Accountancy* 2012;**15**:527.

Rashad AS, Sharaf MF. Catastrophic and impoverishing effects of out-of-pocket health expenditure: New evidence from Egypt. *American Journal of Economics* 2015;**5**:526–33.

Salahuddin M, Vink N, Ralph N *et al.* Globalisation, poverty and corruption: Retarding progress in South Africa. *Dev South Afr* 2019;**37**:617–43.

Shabbir G, Anwar M, Adil Shahid. Corruption, Political Stability and Economic Growth. *The Pakistan Development Review,* 2016;**55**:689–702.

Shumetie A, Watabaji MD. Effect of corruption and political instability on enterprises’ innovativeness in Ethiopia: Pooled data based. *J Innov Entrep* 2019;**8**:1–19.

The Borgen Project. How Politics Affect Poverty . 2018.

Vian T. Review of corruption in the health sector: theory, methods and interventions. *Health Policy Plan* 2008;**23**:83–94.

Wagstaff A, van Doorslaer E. Catastrophe and impoverishment in paying for health care: with applications to Vietnam 1993-1998. *Health Econ* 2003;**12**:921–33.

Wagstaff A, Flores G, Hsu J *et al.* Progress on catastrophic health spending in 133 countries: a retrospective observational study. *Lancet Glob Health* 2018a;**6**:e169–79.

Wagstaff A, Flores G, Smitz MF *et al.* Progress on impoverishing health spending in 122 countries: a retrospective observational study. *Lancet Glob Health* 2018b;**6**:e180–92.

Wagstaff A, Neelsen S. A comprehensive assessment of universal health coverage in 111 countries: a retrospective observational study. *Lancet Glob Health* 2020;**8**:e39–49.

World Health Organization. Primary health care on the road to universal health coverage: 2019 global monitoring report: executive summary. *World Health Organization* 2019:151.

Xu K, Evans DB, Carrin G *et al.* Protecting households from catastrophic health spending. *Health Aff* 2007;**26**:972–83.
