# Supplementary material for: Assessing the relationship between coverage of essential health services and poverty levels in low- and middle-income countries
Source: Health Policy Plan. 2024 Feb 1;39(2):156–67. doi: 10.1093/heapol/czae002 (PMC10883664; doi:10.1093/heapol/czae002)
Supplement: czae002_Supp [file czae002_supp.zip › suppl_data/Appendix II - Forward model building amended.docx]

**Appendix II - Forward model building: using AIC and BIC to choose a final model based on fixed- effect regressions of UHC indicators on poverty measures**

We employed fixed-effects regression analyses on the main dataset composed of 96 LMIC to build the final regression models thereafter used in the Hausman tests. This was done by independently adding identified confounders (see Appendix II) to a baseline model, for each outcome variable (6 poverty measures) on each exposure variable (8 UHC indicators). For each resulting model, we obtained the Bayesian information criterion (BIC) and the Aikake information criterion (AIC). Both are criteria for selection of econometric models that help to prevent overfitting. Models with lower BIC and AIC values indicate better fitting models and so are preferred. To assess the strength of the evidence given by BIC and AIC for a model against another, we calculated delta (𝛥) AIC and BIC of candidate models. These are the difference between a candidate model and the model providing the lowest AIC or BIC (also known as the best model). If 𝛥AIC or 𝛥BIC are less than 2, there is evidence for the candidate model over the best model. Here, the candidate model was chosen if there was support from both AIC and BIC (i.e. if both resulted in Δ below 2). Furthermore, BIC is more conservative than AIC in the sense that it more strongly penalises the model for the number of parameters included. As such, models were not chosen if AIC stated the candidate model was a better fit but BIC did not.

The confounders included in these processes were: GDP per capita, CHE, trade, control of corruption, and absence of political instability and violence/terrorism (PSAV). These forward model building strategies resulted in different confounders being included in each regression. All final models of each poverty variable on each UHC indicator included GDP per capita and CHE. Most also included trade, except for inpatient admissions models with the poverty headcount ratio at $5.50-per-day, and the poverty gap at $3.20 and at $5.50-per-day. APIV was included in all cervical cancer screening models; in the regressions of the poverty gap at $1.90-per-day on inpatient admissions, full immunization rates, and antenatal care utilization, and in the regressions of the poverty headcount ratio at $1.90-per-day on inpatient admissions, diarrhoea treatment, and full immunization rates. The model for antenatal care utilization and the poverty headcount ratio at $5.50-per-day also included APIV. Control of corruption was included in most breast cancer screening models, except with the poverty gap at $1.90-per-day, and in the inpatient admissions model with the poverty headcount ratio at $1.90-per-day.

# Tables B1 Fixed-effect regressions of inpatient admissions on poverty

|  | **Baseline model** | | **+ GDP** | | **+ Current health expenditure** | | **+ Control of corruption** | | **+ Absence of political instability and violence** | | **+ Trade**** | |
| --- | --- | --- | --- | --- | --- | --- | --- | --- | --- | --- | --- | --- |
|  | Coefficient (95% CI) | p-value | Coefficient (95% CI) | p-value | Coefficient (95% CI) | p-value | Coefficient (95% CI) | p-value | Coefficient (95% CI) | p-value | Coefficient (95% CI) | p-value |
| **Inpatient admissions** | -0.387  (-0.893 – 0.118) | 0.13 | -0.335  (-0.75 – 0.083) | 0.11 | -0.253  (-0.505 – -0.001) | 0.05 | -0.250  (-0.510 – 0.011) | 0.06 | -0.240  (-0.489 – 0.008) | 0.06 | -0.222  (-0.472 – 0.029) | 0.08 |
| **GDP per capita, PPP (2011 $)** |  |  | -0.0007  (-0.0012 – -0.0001) | 0.02 | -0.0005  (-0.001 – -0.0001) | 0.03 | -0.0004  (-0.001 – -0.00004) | 0.03 | -0.0004  (-0.001 – -0.00003) | 0.07 | -0.0005  (-0.001 – 0.00001) | 0.05 |
| **Current health expenditure** |  |  |  |  | -0.625  (-1.17 – -0.077) | 0.03 | -0.631  (-1.20 – -0.059) | 0.03 | -0.671  (-1.18 – -0.162) | 0.01 | -0.593  (-1.11 – -0.143) | 0.01 |
| **Control of corruption** |  |  |  |  |  |  | -1.01  (-3.16 – 1.14) | 0.35 |  |  |  |  |
| **Absence of political instability and violence** |  |  |  |  |  |  |  |  | -0.956  (-2.54 – 0.624) | 0.23 | -1.13  (-2.62 – 0.368) | 0.14 |
| **Trade (% of GDP)** |  |  |  |  |  |  |  |  |  |  | -0.017  (-0.072 – 0.037) | 0.53 |
| **AIC** | 794.73 | | 755.33 | | 538.21 | | 538.19 | | 535.99 | | 533.36 | |
| **BIC** | 797.94 | | 761.73 | | 547.52 | | 550.61 | | 548.41 | | 548.86 | |

**Table B1.1 Fixed-effect regressions of inpatient admissions and potential confounders on the poverty gap at $1.90-per-day**

**final model

**Table B1.2. Fixed-effect regressions of inpatient admissions and potential confounders on the poverty gap at $3.20-per-day.**

|  | **Baseline model** | | **+ GDP** | | **+ Current health expenditure**** | | **+ Control of corruption** | | **+ Absence of political instability and violence-** | | **+ Trade** | |
| --- | --- | --- | --- | --- | --- | --- | --- | --- | --- | --- | --- | --- |
|  | Coefficient (95% CI) | p-value | Coefficient (95% CI) | p-value | Coefficient (95% CI) | p-value | Coefficient (95% CI) | p-value | Coefficient (95% CI) | p-value | Coefficient (95% CI) | p-value |
| **Inpatient admissions** | -0.788  (-1.80 – 0.223) | 0.12 | -0.655  (-1.37 – 0.063) | 0.07 | -0.588  (-1.07 – 0.107) | 0.017 | -0.578  (-1.18 – 0.037) | 0.02 | -0.557  (-1.01 – -0.100) | 0.018 | -0.583  (-1.07 – -0.093) | 0.02 |
| **GDP per capita, PPP (2011 $)** |  |  | -0.002  (-0.003 – -0.0003) | 0.02 | -0.001  (-0.002 – -0.0002) | 0.018 | -0.001  (-0.003 – -0.0003) | 0.018 | -0.001  (-0.002 – -0.0004) | 0.04 | -0.001  (-0.002 – -0.0002) | 0.018 |
| **Current health expenditure** |  |  |  |  | -1.52  (-2.75 – -0.296) | 0.02 | -1.54  (-2.81 – -0.274) | 0.018 | -1.64  (-2.77 – -0.497) | 0.006 | -1.55  (-2.66 – -0.437) | 0.007 |
| **Control of corruption** |  |  |  |  |  |  | -3.09  (-8.87 – 2.69) | 0.29 |  |  |  |  |
| **Absence of political instability and violence** |  |  |  |  |  |  |  |  | -2.33  (-5.49 – 0.820) | 0.14 |  |  |
| **Trade (% of GDP)** |  |  |  |  |  |  |  |  |  |  | -0.006  (-0.150 – 0.139) | 0.94 |
| **AIC** | 1065.17 | | 1005.87 | | 782.99 | | 782.74 | | 781.33 | | 781.27 | |
| **BIC** | 1068.38 | | 1012.27 | | 794.31 | | 795.16 | | 793.76 | | 793.66 | |

**final model

**Table B1.3. Fixed-effect regressions of inpatient admissions and potential confounders on the poverty gap at $5.50-per-day.**

|  | **Baseline model** | | **+ GDP** | | **+ Current health expenditure **** | | **+ Control of corruption** | | **+ Absence of political instability and violence** | | **+ Trade** | |
| --- | --- | --- | --- | --- | --- | --- | --- | --- | --- | --- | --- | --- |
|  | Coefficient (95% CI) | p-value | Coefficient (95% CI) | p-value | Coefficient (95% CI) | p-value | Coefficient (95% CI) | p-value | Coefficient (95% CI) | p-value | Coefficient (95% CI) | p-value |
| **Inpatient admissions** | -1.05  (-2.59 – 0.483) | 0.18 | -0.828  (-1.84 – 0.182) | 0.11 | -0.782  (-1.41 – -0.153) | 0.016 | -0.771  (-1.43 – -0.138) | 0.018 | -0.749  (-1.36 – -0.140) | 0.017 | -0.777  (-1.38 – -0.173) | 0.01 |
| **GDP per capita, PPP (2011 $)** |  |  | -0.003  (-0.005 – 0.001) | 0.003 | -0.002  (-0.004 – -0.001) | 0.003 | -0.002  (-0.004 – -0.001) | 0.003 | -0.002  (-0.004 – -0.001) | 0.008 | -0.002  (-0.004 – -0.001) | 0.003 |
| **Current health expenditure** |  |  |  |  | -2.27  (-4.07 – -0.471) | 0.01 | -2.29  (-4.13 – -0.453) | 0.015 | -2.39  (-4.11 – -0.666) | 0.007 | -2.31  (-3.87 – -0.747) | 0.004 |
| **Control of corruption** |  |  |  |  |  |  | -3.33  (-11.2 – 4.58) | 0.40 |  |  |  |  |
| **Absence of political instability and violence** |  |  |  |  |  |  |  |  | -3.49  (-6.29 – 2.06) | 0.28 |  | 0.005 |
| **Trade (% of GDP)** |  |  |  |  |  |  |  |  |  |  | -0.004  (-0.218 – 0.210) | 0.97 |
| AIC | 1202.57 | | 1117.02 | | 914.29 | | 914.05 | | 913.49 | | 910.55 | |
| BIC | 1205.78 | | 1123.41 | | 923.61 | | 926.47 | | 925.92 | | 922.95 | |

**final model

|  | **Baseline model** | | **+ GDP** | | **+ Current health expenditure** | | **+ Control of corruption** | | **+ Absence of political instability and violence**** | | **+ Trade** | |
| --- | --- | --- | --- | --- | --- | --- | --- | --- | --- | --- | --- | --- |
|  | Coefficient (95% CI) | p-value | Coefficient (95% CI) | p-value | Coefficient (95% CI) | p-value | Coefficient (95% CI) | p-value | Coefficient (95% CI) | p-value | Coefficient (95% CI) | p-value |
| **Inpatient admissions** | -1.06  (-2.32 – 0.204) | 0.09 | -0.892  (-1.79 – 0.003) | 0.05 | -0.838  (-1.51 – -0.162) | 0.016 | -0.824  (-1.52 – -0.126) | 0.02 | -0.790  (-1.46 – -0.123) | 0.02 | -0.765  (-1.44 – -0.094) | 0.03 |
| **GDP per capita, PPP (2011 $)** |  |  | -0.002  (-0.004 – -0.0003) | 0.02 | -0.002  (-0.003 – -0.0002) | 0.02 | -0.001  (-0.003 – -0.0002) | 0.02 | -0.001  (-0.003 – -0.0003) | 0.06 | -0.001  (-0.003 – -0.000002) | 0.05 |
| **Current health expenditure (% of GDP)** |  |  |  |  | -1.92  (-3.51 – -0.330) | 0.02 | -1.94  (-3.61 – -0.291) | 0.02 | -2.08  (-3.57 – -0.585) | 0.007 | -2.06  (-3.44 – -0.673) | 0.01 |
| **Control of corruption** |  |  |  |  |  |  | -4.33  (-12.5 – 3.85) | 0.30 | -3.34  (-11.3 – 4.65) | 0.41 | -3.28  (-11.7 – 5.11) | 0.44 |
| **Absence of political instability and violence** |  |  |  |  |  |  |  |  | -2.84  (-7.15 – 1.47) | 0.193 | -2.92  (-6.31 - 0.465) | 0.09 |
| **Trade (% of GDP)** |  |  |  |  |  |  |  |  |  |  | -0.025  (-0.216 – 0.166) | 0.79 |
| AIC | 1173.52 | | 1121.03 | | 877.62 | | 874.86 | | 872.21 | | 867.28 | |
| BIC | 1176.73 | | 1127.42 | | 886.94 | | 887.29 | | 887.74 | | 885.88 | |

**Table B1.4. Fixed-effect regressions of inpatient admissions and potential confounders on the poverty headcount ratio at $1.90-per-day.**

**Table B1.5. Fixed-effect regressions of inpatient admissions and potential confounders on the poverty headcount ratio at $3.20-per-day**

|  | **Baseline model** | | **+ GDP** | | **+ Current health expenditure** | | **+ Control of corruption** | | **+ Absence of political instability and violence** | | **+ Trade**** | |
| --- | --- | --- | --- | --- | --- | --- | --- | --- | --- | --- | --- | --- |
|  | Coefficient (95% CI) | p-value | Coefficient (95% CI) | p-value | Coefficient (95% CI) | p-value | Coefficient (95% CI) | p-value | Coefficient (95% CI) | p-value | Coefficient (95% CI) | p-value |
| **Inpatient admissions** | -1.51  (-3.75 – 0.736) | 0.18 | -1.19  (-2.68 – 0.301) | 0.12 | -1.18  (-2.21 – -0.157) | 0.02 | -1.16  (-2.18 – -0.134) | 0.03 | -1.16  (-2.18 – -0.134) | 0.03 | -1.19  (-2.18 – -0.193) | 0.02 |
| **GDP per capita** |  |  | -0.004  (-0.007 – -0.001) | 0.009 | -0.003  (-0.006 – -0.001) | 0.01 | -0.003  (-0.006 – -0.001) | 0.01 | -0.003  (-0.006 – -0.001) | 0.01 | -0.003  (-0.006 – -0.001) | 0.01 |
| **Current health expenditure** |  |  |  |  | -3.50  (-6.23 – -0.756) | 0.01 | -3.54  (-6.33 – -0.747) | 0.02 | -3.54  (-6.33 – -0.747) | 0.02 | -3.61  (-5.98 – -1.24) | 0.003 |
| **Control of corruption** |  |  |  |  |  |  | -6.68  (19.8 – 6.43) | 0.31 |  |  |  |  |
| **Absence of political instability and violence** |  |  |  |  |  |  |  |  | -4.47  (-11.9 – 3.01) | 0.24 |  |  |
| **Trade** |  |  |  |  |  |  |  |  |  |  | 0.009  (-0.322 – 0.339) | 0.95 |
| **AIC** | 1346.76 | | 1270.30 | | 1064.45 | | 1062.80 | | 1062.65 | | 1059.27 | |
| **BIC** | 1349.97 | | 1276.70 | | 1073.77 | | 1075.23 | | 1075.07 | | 1071.67 | |

**final model

**Table B1.6. Fixed-effect regressions of inpatient admissions and potential confounders on the poverty headcount ratio at $5.50-per-day**

|  | **Baseline model** | | **+ GDP** | | **+ Current health expenditure**** | | **+ Control of corruption** | | **+ Absence of political instability and violence** | | **+ Trade** | |
| --- | --- | --- | --- | --- | --- | --- | --- | --- | --- | --- | --- | --- |
|  | Coefficient (95% CI) | p-value | Coefficient (95% CI) | p-value | Coefficient (95% CI) | p-value | Coefficient (95% CI) | p-value | Coefficient (95% CI) | p-value | Coefficient (95% CI) | p-value |
| **Inpatient admissions** | -1.33  (-3.45 – 0.797) | 0.22 | -0.955  (-2.23 – 0.322) | 0.14 | -0.922  (-1.69 – -0.152) | 0.02 | -0.922  (-1.68 – -0.162) | 0.02 | -0.915  (-1.70 – -0.133) | 0.02 | -0.890  (-1.56 – -0.218) | 0.01 |
| **GDP per capita** |  |  | -0.005  (-0.007 – -0.003) | <0.001 | -0.004  (-0.006 – -0.003) | <0.001 | -0.004  (-0.006 – -0.003) | <0.001 | -0.004  (-0.006 – -0.002) | <0.001 | -0.004  (-0.006 – -0.003) | <0.001 |
| **Current health expenditure** |  |  |  |  | -2.65  (-4.93 – -0.360) | 0.02 | -2.65  (-4.96 – -0.332) | 0.03 | -2.67  (-4.95 – -0.396) | 0.02 | -2.58  (-4.48 – -0.671) | 0.009 |
| **Control of corruption** |  |  |  |  |  |  | 0.249  (-7.71 – 8.20) | 0.95 |  |  |  |  |
| **Absence of political instability and violence** |  |  |  |  |  |  |  |  | -0.463  (-5.72 – 4.80) | 0.86 |  |  |
| **Trade** |  |  |  |  |  |  |  |  |  |  | -0.033  (-0.297 – 0.230) | 0.80 |
| **AIC** | 1324.02 | | 1196.78 | | 999.79 | | 1001.78 | | 1001.73 | | 995.87 | |
| **BIC** | 1327.23 | | 1203.17 | | 1009.11 | | 1014.20 | | 1014.15 | | 1008.27 | |

**final model

# Tables B2: Fixed-effect regressions of skilled birth attendance on poverty

Table B2.1 Fixed-effect regressions of skilled birth attendance on poverty gap at $1.90-per-day approach.

|  | **Baseline model** | | **+ GDP** | | **+ Current health expenditure** | | **+ Control of corruption** | | **+ Absence of political instability and violence** | | **+ Trade**** | |
| --- | --- | --- | --- | --- | --- | --- | --- | --- | --- | --- | --- | --- |
|  | Coefficient (95% CI) | p-value | Coefficient (95% CI) | p-value | Coefficient (95% CI) | p-value | Coefficient (95% CI) | p-value | Coefficient (95% CI) | p-value | Coefficient (95% CI) | p-value |
| **Skilled birth attendance** | -0.240  (-0.319 - -0.162) | <0.001 | -0.237  (-0.321 - -0.153) | <0.001 | -0.218  (-0.304 - -0.132) | <0.001 | -0.218  (-0.304 – -0.130) | <0.001 | -0.218  (-0.304 – -0.130) | <0.001 | -0.209  (-0.290 – -0.127) | <0.001 |
| **GDP per capita** |  |  | -0.0001  (-0.0001 - 0.0001) | 0.35 | -0.0001  (-0.0004 - 0.0002) | 0.56 | -0.0001  (-0.0004 - 0.0002) | 0.55 | -0.0001  (-0.0004 - 0.0002) | 0.55 | -0.0001  (-0.0004 - 0.0002) | 0.46 |
| **Current health expenditure** |  |  |  |  | -0.589  (-1.43 – 0.254) | 0.17 | -0.592  (-1.45 – 0.272) | 0.18 | -0.601  (-1.45 – 0.272) | 0.16 | -0.427  (-1.22 – 0.370) | 0.29 |
| **Control of corruption** |  |  |  |  |  |  | 0.132  (-3.70 – 3.97) | 0.95 |  |  |  |  |
| **Absence of political instability and violence** |  |  |  |  |  |  |  |  | -0.575  (-2.32 – 1.17) | 0.51 |  |  |
| **Trade** |  |  |  |  |  |  |  |  |  |  | -0.007  (-0.076 – 0.063) | 0.85 |
| **AIC** | 1506.28 | | 1482.06 | | 1128.12 | | 1130.11 | | 1129.24 | | 1090.20 | |
| **BIC** | 1059.91 | | 1489.29 | | 1138.37 | | 1143.78 | | 1142.90 | | 1103.78 | |

**final model

**Table B2.2 Fixed-effect regressions of skilled birth attendance on poverty gap at $3.20-per-day**

|  | **Baseline model** | | **+ GDP** | | **+ Current health expenditure** | | **+ Control of corruption** | | **+ Absence of political instability and violence** | | **+ Trade**** | |
| --- | --- | --- | --- | --- | --- | --- | --- | --- | --- | --- | --- | --- |
|  | Coefficient (95% CI) | p-value | Coefficient (95% CI) | p-value | Coefficient (95% CI) | p-value | Coefficient (95% CI) | p-value | Coefficient (95% CI) | p-value | Coefficient (95% CI) | p-value |
| **Skilled birth attendance** | -0.353  (-0.462 – -0.244) | <0.001 | -0.312  (-0.249 – -0.197) | <0.001 | -0.276  (-0.391 – -0.160) | <0.001 | -0.280  (-0.396 – -0.165) | <0.001 | -0.272  (-0.393 – -0.152) | <0.001 | -0.264  (-0.374 – -0.155) | <0.001 |
| **GDP per capita** |  |  | -0.001  (-0.001 - -0.0004) | <0.001 | -0.001  (-0.001 - -0.0004) | <0.001 | -0.001  (-0.001 - -0.0005) | <0.001 | -0.001  (-0.001 - -0.0004) | <0.001 | -0.001  (-0.001 - -0.0004) | <0.001 |
| **Current health expenditure** |  |  |  |  | -0.823  (-1.88 – 0.232) | 0.124 | -0.852  (-1.93 – 0.222) | 0.12 | -0.839  (-1.91 – 0.231) | 0.12 | -0.653  (-1.68 – 0.371) | 0.21 |
| **Control of corruption** |  |  |  |  |  |  | 1.51  (-3.95 – 6.97) | 0.59 |  |  |  |  |
| **Absence of political instability and violence** |  |  |  |  |  |  |  |  | -0.465  (-2.41 – 1.48) | 0.64 |  |  |
| **Trade** |  |  |  |  |  |  |  |  |  |  | -0.011  (-0.099 – 0.078) | 0.81 |
| **AIC** | 1629.24 | | 1524.29 | | 1220.86 | | 1222.19 | | 1222.48 | | 1186.16 | |
| **BIC** | 1632.87 | | 1601.53 | | 1231.11 | | 1235.86 | | 1236.15 | | 1199.73 | |

**final model

**Table B2.3 Fixed-effect regressions of skilled birth attendance on poverty gap at $5.50-per-day**

|  | **Baseline model** | | **+ GDP** | | **+ Current health expenditure** | | **+ Control of corruption** | | **+ Absence of political instability and violence** | | **+ Trade**** | |
| --- | --- | --- | --- | --- | --- | --- | --- | --- | --- | --- | --- | --- |
|  | Coefficient (95% CI) | p-value | Coefficient (95% CI) | p-value | Coefficient (95% CI) | p-value | Coefficient (95% CI) | p-value | Coefficient (95% CI) | p-value | Coefficient (95% CI) | p-value |
| **Skilled birth attendance** | -0.391  (-0.520 - -0.262) | <0.001 | -0.288  (-0.410 - -0.166) | <0.001 | -0.249  (-0.382 – -0.116) | <0.001 | -0.257  (-0.385 – -0.130) | <0.001 | -0.252  (-0.388 – -0.116) | <0.001 | -0.238  (-0.367 – -0.109) | <0.001 |
| **GDP per capita** |  |  | -0.002  (-0.003 - -0.001) | <0.001 | -0.002  (-0.003 - -0.002) | <0.001 | -0.002  (-0.003 - -0.002) | <0.001 | -0.002  (-0.003 - -0.002) | <0.001 | -0.002  (-0.003 - -0.002) | <0.001 |
| **Current health expenditure** |  |  |  |  |  |  | -1.16  (-2.30 – -0.006) | 0.05 | -1.08  (-2.22 – 0.053) | 0.06 | -0.955  (-2.08 – 0.173) | 0.09 |
| **Control of corruption** |  |  |  |  |  |  | 3.04  (-3.35 – 9.43) | 0.35 |  |  |  |  |
| **Absence of political instability and violence** |  |  |  |  |  |  |  |  | 0.440  (-1.58 – 2.46) | 0.67 |  |  |
| **Trade** |  |  |  |  |  |  |  |  |  |  | -0.016  (-0.121 – 0.090) | 0.77 |
| **AIC** | 1723.25 | | 1634.82 | | 1282.41 | | 1282.33 | | 1284.15 | | 1250.59 | |
| **BIC** | 1726.89 | | 1642.05 | | 1292.66 | | 1295.99 | | 1297.82 | | 1264.16 | |

**final model

**Table B2.4 Fixed-effect regressions of skilled birth attendance on poverty headcount ratio at $1.90-per-day**

|  | **Baseline model** | | **+ GDP** | | **+ Current health expenditure** | | **+ Control of corruption** | | **+ Absence of political instability and violence** | | **+ Trade**** | |
| --- | --- | --- | --- | --- | --- | --- | --- | --- | --- | --- | --- | --- |
|  | Coefficient (95% CI) | p-value | Coefficient (95% CI) | p-value | Coefficient (95% CI) | p-value | Coefficient (95% CI) | p-value | Coefficient (95% CI) | p-value | Coefficient (95% CI) | p-value |
| **Skilled birth attendance** | -0.487  (-0.650 – -0.324) | <0.001 | -0.445  (-0.622 – 0.268) | <0.001 | -0.386  (0.550 - -0.222) | <0.001 | -0.393  (-0.555 - -0.231) | <0.001 | -0.379  (-0.549 – -0.209) | <0.001 | -0.372  (-0.529 – -0.216) | <0.001 |
| **GDP per capita** |  |  | -0.001  (-0.001 – -0.0003) | 0.00219 | -0.001  (-0.001 – -0.0003) | 0.002 | -0.001  (-0.002 – -0.0004) | 0.002 | -0.001  (-0.002 – 0.0003) | 0.004 | -0.001  (-0.002 – -0.0003) | 0.005 |
| **Current health expenditure** |  |  |  |  | -0.924  (-2.38 – 0.534) | 0.21 | -0.974  (-2.46 – 0.507) | 0.20 | -0.958  (-2.44 – 0.528) | 0.20 | -0.702  (-2.14 – 0.734) | 0.33 |
| **Control of corruption** |  |  |  |  |  |  | 2.54  (-5.21 – 10.3) | 0.52 |  |  |  |  |
| **Absence of political instability and violence** |  |  |  |  |  |  |  |  | -0.992  (-3.82 – 1.84) | 0.49 |  |  |
| **Trade** |  |  |  |  |  |  |  |  |  |  | -0.002  (-0.124 – 0.119) | 0.97 |
| **AIC** | 1825.64 | | 1792.81 | | 1370.71 | | 1371.73 | | 1371.82 | | 1335.49 | |
| **BIC** | 1829.28 | | 1800.04 | | 1380.96 | | 1385.40 | | 1385.49 | | 1349.06 | |

**final model

**Table B2.5 Fixed-effect regressions of skilled birth attendance on poverty headcount ratio at $3.20-per-day**

|  | Baseline model | | + GDP | | + Current health expenditure | | + Control of corruption | | + Absence of political instability and violence | | + Trade** | |
| --- | --- | --- | --- | --- | --- | --- | --- | --- | --- | --- | --- | --- |
|  | Coefficient (95% CI) | p-value | Coefficient (95% CI) | p-value | Coefficient (95% CI) | p-value | Coefficient (95% CI) | p-value | Coefficient (95% CI) | p-value | Coefficient (95% CI) | p-value |
| Skilled birth attendance | -0.514  (-0.708 - -0.320) | <0.001 | -0.371  (-0.554 - -0.189) | <0.001 | -0.311  (-0.514 - -0.108) | 0.003 | -0.324  (-0.512 – -0.136) | 0.001 | -0.314  (-0.519 - -0.109) | 0.003 | -0.298  (-0.497 - -0.098) | 0.004 |
| GDP per capita |  |  | -0.003  (-0.004 – -0.002) | <0.001 | -0.003  (-0.004 – -0.002) | <0.001 | -0.003  (-0.004 – -0.002) | <0.001 | -0.003  (-0.004 - -0.002) | <0.001 | -0.003  (-0.004 - -0.002) | <0.001 |
| Current health expenditure |  |  |  |  | -1.43  (-3.02 – 0.164) | 0.08 | -1.52  (-3.12 – 0.092) | 0.06 | -1.41  (-2.99 – 0.174) | 0.08 | -1.28  (-2.90 – 0.335) | 0.12 |
| Control of corruption |  |  |  |  |  |  | 4.57  (-4.82 – 13.9) | 0.34 |  |  |  |  |
| Absence of political instability and violence |  |  |  |  |  |  |  |  | 0.410  (-2.47 – 3.29) | 0.78 |  |  |
| Trade |  |  |  |  |  |  |  |  |  |  | -0.021  (-0.175 – 0.133) | 0.78 |
| AIC | 1909.51 | | 1821.50 | | 1445.74 | | 1445.46 | | 1447.63 | | 1414.84 | |
| BIC | 1913.15 | | 1828.74 | | 1455.99 | | 1459.13 | | 1461.30 | | 1428.42 | |

**final model

**Table B2.6 Fixed-effect regressions of skilled birth attendance on poverty headcount ratio at $5.50-per-day**

|  | Baseline model | | + GDP | | + Current health expenditure | | + Control of corruption | | + Absence of political instability and violence | | + Trade** | |
| --- | --- | --- | --- | --- | --- | --- | --- | --- | --- | --- | --- | --- |
|  | Coefficient (95% CI) | p-value | Coefficient (95% CI) | p-value | Coefficient (95% CI) | p-value | Coefficient (95% CI) | p-value | Coefficient (95% CI) | p-value | Coefficient (95% CI) | p-value |
| Skilled birth attendance | -0.374  (-0.541 - -0.207) | <0.001 | -0.155  (-0.269 - -0.041) | 0.008 | -0.123  (-0.258 - -0.012) | 0.07 | -0.136  (-0.259 - -0.014) | 0.03 | -0.141  (-0.279 - -0.003) | 0.05 | -0.118  (-0.254 – 0.018) | 0.09 |
| GDP per capita |  |  | -0.004  (-0.005 – -0.003) | <0.001 | -0.003  (-0.004 – -0.002) | <0.001 | -0.003  (-0.004 – -0.002) | <0.001 | -0.005  (-0.006 - -0.004) | <0.001 | -0.005  (-0.006 - -0.004) | <0.001 |
| Current health expenditure |  |  |  |  | -1.32  (-2.48 - -0.153) | 0.03 | -1.41  (-2.59 – -0.237) | 0.02 | -1.23  (-2.37 - -0.095) | 0.03 | -1.26  (-2.46 - -0.066) | 0.04 |
| Control of corruption |  |  |  |  |  |  | 4.77  (-2.78 – 12.3) | 0.21 |  |  |  |  |
| Absence of political instability and violence |  |  |  |  |  |  |  |  | 2.58  (-0.052 – 5.20) | 0.06 |  |  |
| Trade |  |  |  |  |  |  |  |  |  |  | -0.025  (-0.154 – 0.104) | 0.70 |
| AIC | 1931.12 | | 1731.66 | | 1395.23 | | 1394.11 | | 1391.78 | | 1357.18 | |
| BIC | 1934.76 | | 1738.89 | | 1405.48 | | 1407.78 | | 1405.44 | | 1370.76 | |

**final model

# Tables B3: Fixed-effect regressions of diarrhoea treatment on poverty

**Table B3.1 Fixed-effect regressions of diarrhoea treatment on poverty gap at $1.90-per-day**

|  | **Baseline model** | | **+ GDP** | | **+ Current health expenditure** | | **+ Control of corruption** | | **+ Absence of political instability and violence** | | **+ Trade**** | |
| --- | --- | --- | --- | --- | --- | --- | --- | --- | --- | --- | --- | --- |
|  | Coefficient (95% CI) | p-value | Coefficient (95% CI) | p-value | Coefficient (95% CI) | p-value | Coefficient (95% CI) | p-value | Coefficient (95% CI) | p-value | Coefficient (95% CI) | p-value |
| **Diarrhoea treatment** | -0.231  (-0.347 - -0.114) | <0.001 | -0.175  (-0.301 - -0.049) | 0.007 | -0.264  (-0.404 - -0.125) | <0.001 | -0.262  (-0.400 - -0.124) | <0.001 | -0.258  (-0.404 – -0.112) | 0.001 | -0.267  (-0.408 - -0.127) | <0.001 |
| **GDP per capita** |  |  | -0.001  (-0.001 - -0.0001) | 0.03 | -0.0001  (-0.001 - -0.0006) | 0.84 | -0.0001  (-0.001 - -0.0006) | 0.83 | -0.00003  (-0.001 - -0.0007) | 0.92 | -0.0001  (-0.001 – 0.0005) | 0.75 |
| **Current health expenditure** |  |  |  |  | -0.581  (-1.40 – 0.238) | 0.16 | -0.555  (-1.36 – 0.248) | 0.17 | -0.587  (-1.40 – 0.229) | 0.16 | -0.574  (-1.41 – 0.259) | 0.17 |
| **Control of corruption** |  |  |  |  |  |  | -1.24  (-6.92 – 4.43) | 0.66 |  |  |  |  |
| **Absence of political instability and violence** |  |  |  |  |  |  |  |  | -1.29  (-4.10 – 1.53) | 0.37 |  |  |
| **Trade** |  |  |  |  |  |  |  |  |  |  | -0.003  (-0.082 – 0.077) | 0.95 |
| **AIC** | 1391.50 | | 1344.55 | | 993.76 | | 995.20 | | 992.74 | | 977.94 | |
| **BIC** | 1394.99 | | 1351.49 | | 1003.58 | | 1008.29 | | 1005.83 | | 990.94 | |

**final model

**Table B3.2 Fixed-effect regressions of diarrhoea treatment on poverty gap at $3.20-per-day**

|  | Baseline model | | + GDP | | + Current health expenditure | | + Control of corruption | | + Absence of political instability and violence | | + Trade** | |
| --- | --- | --- | --- | --- | --- | --- | --- | --- | --- | --- | --- | --- |
|  | Coefficient (95% CI) | p-value | Coefficient (95% CI) | p-value | Coefficient (95% CI) | p-value | Coefficient (95% CI) | p-value | Coefficient (95% CI) | p-value | Coefficient (95% CI) | p-value |
| Diarrhoea treatment | 0.326  (-0.477 - -0.176) | <0.001 | 0.216  (-0.381 - -0.051) | 0.01 | 0.332  (-0.509 - -0.155) | <0.001 | -0.331  (-0.511 - -0.152) | <0.001 | -0.324  (-0.511 - -0.136) | 0.001 | -0.335  (-0.514 – -0.156) | <0.001 |
| GDP per capita |  |  | -0.002  (-0.003 - -0.001) | <0.001 | -0.001  (-0.002 - -0.0004) | 0.04 | -0.001  (-0.002 - -0.0004) | 0.04 | -0.001  (-0.002 - 0.0001) | 0.07 | -0.001  (-0.002 - -0.00006) | 0.04 |
| Current health expenditure |  |  |  |  | -0.698  (-1.67 – 0.278) | 0.16 | -0.692  (-1.66 – 0.279) | 0.16 | -0.707  (-1.69 – 0.278) | 0.16 | -0.687  (-1.68 – 0.309) | 0.17 |
| Control of corruption |  |  |  |  |  |  | -0.312  (-6.72 – 6.10) | 0.92 |  |  |  |  |
| Absence of political instability and violence |  |  |  |  |  |  |  |  | -1.61  (-4.74 – 1.53) | 0.31 |  |  |
| Trade |  |  |  |  |  |  |  |  |  |  | 0.006  (-0.078 – 0.092) | 0.88 |
| AIC | 1490.22 | | 1425.26 | | 1067.49 | | 1069.47 | | 1066.27 | | 1050.95 | |
| BIC | 1493.72 | | 1432.19 | | 1077.31 | | 1082.56 | | 1079.37 | | 1063.96 | |

**final model

**Table B3.3 Fixed-effect regressions of diarrhoea treatment on poverty gap at $5.50-per-day**

|  | Baseline model | | + GDP | | + Current health expenditure | | + Control of corruption | | + Absence of political instability and violence | | + Trade** | |
| --- | --- | --- | --- | --- | --- | --- | --- | --- | --- | --- | --- | --- |
|  | Coefficient (95% CI) | p-value | Coefficient (95% CI) | p-value | Coefficient (95% CI) | p-value | Coefficient (95% CI) | p-value | Coefficient (95% CI) | p-value | Coefficient (95% CI) | p-value |
| Diarrhoea treatment | -0.369  (-0.562 - -0.176) | <0.001 | -0.204  (-0.407 - -0.002) | 0.05 | -0.327  (-0.568 – -0.085) | 0.009 | -0.330  (-0.573 - -0.087) | 0.008 | -0.323  (-0.571 - -0.074) | 0.01 | -0.326  (-0.573 - -0.079) | 0.01 |
| GDP per capita |  | <0.001 | -0.003  (-0.004 - -0.002) | 0.001 | -0.002  (-0.004 - -0.001) | 0.001 | -0.002  (-0.004 - -0.001) | 0.001 | -0.002  (-0.004 - -0.001) | 0.001 | -0.002  (-0.004 - -0.001) | 0.001 |
| Current health expenditure |  |  |  |  | -0.800  (-1.83 – 0.234) | 0.13 | -0.837  (-1.87 – 0.195) | 0.11 | -0.804  (-1.85 – 0.240) | 0.13 | -0.797  (-1.85 – 0.258) | 0.14 |
| Control of corruption |  |  |  |  |  |  | 1.78  (-4.64 – 8.20) | 0.58 |  |  |  |  |
| Absence of political instability and violence |  |  |  |  |  |  |  |  | -0.820  (-3.63 – 1.99) | 0.56 |  |  |
| Trade |  |  |  |  |  |  |  |  |  |  | 0.011  (-0.079 – 0.101) | 0.81 |
| AIC | 1525.20 | | 1425.07 | | 1108.21 | | 1109.57 | | 1109.53 | | 1090.90 | |
| BIC | 1528.70 | | 1432.01 | | 1118.03 | | 1122.67 | | 1122.62 | | 1103.91 | |

**final model

**Table B3.4 Fixed-effect regressions of diarrhoea treatment on poverty headcount ratio at $1.90-per-day**

|  | Baseline model | | + GDP | | + Current health expenditure | | + Control of corruption | | + Absence of political instability and violence | | + Trade** | |
| --- | --- | --- | --- | --- | --- | --- | --- | --- | --- | --- | --- | --- |
|  | Coefficient (95% CI) | p-value | Coefficient (95% CI) | p-value | Coefficient (95% CI) | p-value | Coefficient (95% CI) | p-value | Coefficient (95% CI) | p-value | Coefficient (95% CI) | p-value |
| Diarrhoea treatment | -0.433  (-0.641 – -0.225) | <0.001 | -0.287  (-0.520 - -0.053) | 0.02 | -0.443  (-0.686 – -0.120) | 0.001 | -0.442  (-0.692 - -0.194) | 0.001 | -0.429  (-0.691 - -0.168) | 0.002 | -0.437  (-0.702 - -0.173) | 0.001 |
| GDP per capita |  |  | -0.002  (-0.004 – -0.001) | 0.001 | -0.001  (-0.003 – -0.00003) | 0.05 | -0.001  (-0.003 – -0.00001) | 0.05 | -0.001  (-0.002 – 0.0002) | 0.09 | -0.001  (-0.003 – 0.0001) | 0.08 |
| Current health expenditure |  |  |  |  | -0.771  (-2.16 – 0.613) | 0.27 | -0.772  (-2.15 – 0.603) | 0.27 | -0.785  (-2.19 – 0.617) | 0.27 | -0.760  (-2.20 – 0.678) | 0.30 |
| Control of corruption |  |  |  |  |  |  | 0.032  (-9.11 – 9.17) | 0.99 |  |  |  |  |
| Absence of political instability and violence |  |  |  |  |  |  |  |  | -2.68  (-7.32 – 1.96) | 0.25 | -2.66  (-7.27 – 1.96) | 0.26 |
| Trade |  |  |  |  |  |  |  |  |  |  | 0.017  (-0.098 – 0.133) | 0.76 |
| AIC | 1663.09 | | 1597.81 | | 1204.97 | | 1206.97 | | 1202.52 | | 1182.44 | |
| BIC | 1666.58 | | 1604.75 | | 1214.79 | | 1220.06 | | 1215.62 | | 1198.70 | |

**final model

**Table B3.5 Fixed-effect regressions of diarrhoea treatment on poverty headcount ratio at $3.20-per-day**

|  | **Baseline model** | | **+ GDP** | | **+ Current health expenditure** | | **+ Control of corruption** | | **+ Absence of political instability and violence** | | **+ Trade**** | |
| --- | --- | --- | --- | --- | --- | --- | --- | --- | --- | --- | --- | --- |
|  | Coefficient (95% CI) | p-value | Coefficient (95% CI) | p-value | Coefficient (95% CI) | p-value | Coefficient (95% CI) | p-value | Coefficient (95% CI) | p-value | Coefficient (95% CI) | p-value |
| **Diarrhoea treatment** | -0.462  (-0.752 - -0.171) | 0.002 | -0.235  (-0.537 – 0.068) | 0.13 | -0.385  (-0.757 - -0.136) | 0.04 | -0.391  (-0.766 - -0.016) | 0.04 | -0.379  (-0.760 - 0.001) | 0.05 | -0.387  (-0.768 - -0.007) | 0.05 |
| **GDP per capita** |  |  | -0.004  (-0.006 - -0.002) | <0.001 | -0.004  (-0.006 - -0.002) | <0.001 | -0.004  (-0.006 - -0.002) | <0.001 | -0.003  (-0.005 - -0.001) | 0.001 | -0.003  (-0.005 - -0.001) | 0.001 |
| **Current health expenditure** |  |  |  |  | -0.989  (-2.48 – 0.499) | 0.19 | -1.04  (-2.54 – 0.450) | 0.17 | -0.995  (-2.50 – 0.512) | 0.19 | -0.979  (-2.49 – 0.536) | 0.20 |
| **Control of corruption** |  |  |  |  |  |  | 2.61  (-6.42 – 11.6) | 0.57 |  |  |  |  |
| **Absence of political instability and violence** |  |  |  |  |  |  |  |  | -1.21  (-5.15 – 2.72) | 0.54 |  |  |
| **Trade** |  |  |  |  |  |  |  |  |  |  | 0.024  (-0.103 – 0.151) | 0.71 |
| **AIC** | 1680.98 | | 1572.82 | | 1248.84 | | 1250.17 | | 1250.12 | | 1228.12 | |
| **BIC** | 1684.48 | | 1579.75 | | 1258.66 | | 1263.26 | | 1263.21 | | 1241.13 | |

**final model

**Table B3.6 Fixed-effect regressions of diarrhoea treatment on poverty headcount ratio at $5.50-per-day.**

|  | **Baseline model** | | **+ GDP** | | **+ Current health expenditure** | | **+ Control of corruption** | | **+ Absence of political instability and violence** | | **+ Trade**** | |
| --- | --- | --- | --- | --- | --- | --- | --- | --- | --- | --- | --- | --- |
|  | Coefficient (95% CI) | p-value | Coefficient (95% CI) | p-value | Coefficient (95% CI) | p-value | Coefficient (95% CI) | p-value | Coefficient (95% CI) | p-value | Coefficient (95% CI) | p-value |
| **Diarrhoea treatment** | -0.392  (-0.662 – 0.123) | 0.005 | -0.151  (-0.416 – 0.113) | 0.23 | -0.261  (-0.607 – 0.085) | 0.14 | -0.273  (-0.614 – 0.069) | 0.12 | -0.266  (-0.610 – 0.077) | 0.13 | -0.244  (-0.599 – 0.110) | 0.18 |
| **GDP per capita** |  |  | -0.005  (-0.006 – -0.003) | <0.001 | -0.005  (-0.006 - -0.003) | <0.001 | -0.004  (-0.006 - -0.003) | <0.001 | -0.005  (-0.007 - -0.003) | <0.001 | -0.005  (-0.006 - -0.003) | <0.001 |
| **Current health expenditure** |  |  |  |  | -0.710  (-1.82 – 0.403) | 0.21 | -0.826  (-1.94 – 0.291) | 0.15 | -0.704  (-1.81 – 0.403) | 0.21 | -0.738  (-1.85 – 0.383) | 0.19 |
| **Control of corruption** |  |  |  |  |  |  | 5.54  (-1.80 – 12.9) | 0.14 |  |  |  |  |
| **Absence of political instability and violence** |  |  |  |  |  |  |  |  | 1.09  (-1.50 – 3.68) | 0.41 |  |  |
| **Trade** |  |  |  |  |  |  |  |  |  |  | 0.006  (-0.111 – 0.122) | 0.92 |
| **AIC** | 1623.94 | | 1465.19 | | 1205.53 | | 1203.72 | | 1206.80 | | 1176.86 | |
| **BIC** | 1627.38 | | 1472.12 | | 1215.35 | | 1216.82 | | 1219.89 | | 1189.87 | |

**final model

# Tables B4: Fixed-effect regressions of acute respiratory infection treatment on poverty

**Table B4.1 Fixed-effect regressions of acute respiratory infection treatment in children on poverty gap at $1.90-per-day.**

|  | **Baseline model** | | **+ GDP** | | **+ Current health expenditure** | | **+ Control of corruption** | | **+ Absence of political instability and violence** | | **+ Trade**** | |
| --- | --- | --- | --- | --- | --- | --- | --- | --- | --- | --- | --- | --- |
|  | Coefficient (95% CI) | p-value | Coefficient (95% CI) | p-value | Coefficient (95% CI) | p-value | Coefficient (95% CI) | p-value | Coefficient (95% CI) | p-value | Coefficient (95% CI) | p-value |
| **ARI treatment** | -0.156  (-0.332 – 0.020) | 0.08 | -0.115  (-0.300 – 0.070) | 0.22 | -0.123  (-0.314 – 0.067) | 0.20 | -0.115  (-0.302 – 0.070) | 0.22 | -0.121  (-0.312 – 0.070) | 0.21 | -0.108  (-0.296 – 0.081) | 0.23 |
| **GDP per capita** |  |  | -0.001  (-0.002 - -0.0002) | 0.01 | -0.0007  (-0.001 - -0.0001) | 0.02 | -0.0007  (-0.001 - -0.0001) | 0.02 | -0.0007  (-0.001 - -0.0001) | 0.03 | -0.0007  (-0.001 - -0.0001) | 0.02 |
| **Current health expenditure** |  |  |  |  | -0.648  (-1.88 – 0.581) | 0.30 | -0.634  (-1.86 – 0.594) | 0.31 | -0.659  (-1.91 -0.594) | 0.30 | -0.441  (-1.61 – 0.733) | 0.46 |
| **Control of corruption** |  |  |  |  |  |  | -1.32  (-7.13 – 4.48) | 0.65 |  |  |  |  |
| **Absence of political instability and violence** |  |  |  |  |  |  |  |  | -1.05  (-4.98 – 2.28) | 0.53 |  |  |
| **Trade** |  |  |  |  |  |  |  |  |  |  | -0.010  (-0.124 – 0.103) | 0.86 |
| **AIC** | 1375.78 | | 1340.11 | | 1009.69 | | 1011.22 | | 1010.35 | | 973.83 | |
| **BIC** | 1379.23 | | 1346.99 | | 1019.40 | | 1024.16 | | 1023.30 | | 986.67 | |

**final model

**Table B4.2 Fixed-effect regressions of acute respiratory infection treatment in children on poverty gap at $3.20-per-day.**

|  | **Baseline model** | | **+ GDP** | | **+ Current health expenditure** | | **+ Control of corruption** | | **+ Absence of political instability and violence** | | **+ Trade**** | |
| --- | --- | --- | --- | --- | --- | --- | --- | --- | --- | --- | --- | --- |
|  | Coefficient (95% CI) | p-value | Coefficient (95% CI) | p-value | Coefficient (95% CI) | p-value | Coefficient (95% CI) | p-value | Coefficient (95% CI) | p-value | Coefficient (95% CI) | p-value |
| **ARI treatment** | -0.202  (-0.383 - -0.020) | 0.03 | -0.124  (-0.307 – 0.060) | 0.18 | -0.144  (-0.338 – 0.050) | 0.14 | -0.139  (-0.330 – 0.051) | 0.15 | -0.142  (-0.337 – 0.052) | 0.15 | -0.124  (-0.314 – 0.066) | 0.20 |
| **GDP per capita** |  |  | -0.002  (-0.003 - -0.001) | <0.001 | -0.002  (-0.003 - -0.001) | <0.001 | -0.002  (-0.003 - -0.001) | <0.001 | -0.002  (-0.003 - -0.001) | <0.001 | -0.002  (-0.003 - -0.001) | <0.001 |
| **Current health expenditure** |  |  |  |  | -0.738  (-2.14 – 0.673) | 0.30 | -0.729  (-2.13 – 0.673) | 0.30 | -0.747  (-2.18 – 0.687) | 0.30 | -0.509  (-1.86 – 0.841) | 0.46 |
| **Control of corruption** |  |  |  |  |  |  | -0.812  (-7.21 – 5.59) | 0.80 |  |  |  |  |
| **Absence of political instability and violence** |  |  |  |  |  |  |  |  | -0.872  (-4.41 – 2.67) | 0.63 |  |  |
| **Trade** |  |  |  |  |  |  |  |  |  |  | -0.021  (-0.153 – 0.110) | 0.75 |
| **AIC** | 1473.45 | | 1416.72 | | 1069.97 | | 1071.84 | | 1071.31 | | 1033.57 | |
| **BIC** | 1476.92 | | 1423.61 | | 1079.31 | | 1084.79 | | 1084.25 | | 1046.41 | |

**final model

**Table B4.3 Fixed-effect regressions of acute respiratory infection treatment in children on the poverty gap at $5.50-per-day.**

|  | Baseline model | | + GDP | | + Current health expenditure | | + Control of corruption | | + Absence of political instability and violence | | + Trade** | |
| --- | --- | --- | --- | --- | --- | --- | --- | --- | --- | --- | --- | --- |
|  | Coefficient (95% CI) | p-value | Coefficient (95% CI) | p-value | Coefficient (95% CI) | p-value | Coefficient (95% CI) | p-value | Coefficient (95% CI) | p-value | Coefficient (95% CI) | p-value |
| ARI treatment | -0.214  (-0.378 - -0.050) | 0.01 | -0.098  (-0.248 – 0.051) | 0.20 | -0.111  (-0.271 – 0.050) | 0.17 | -0.112  (-0.270 – 0.046) | 0.16 | -0.111  (-0.271 – 0.049) | 0.17 | -0.088  (-0.244 - 0.068) | 0.27 |
| GDP per capita |  |  | -0.003  (-0.004 - -0.002) | <0.001 | -0.003  (-0.004 - -0.002) | <0.001 | -0.003  (-0.004 - -0.002) | <0.001 | -0.003  (-0.004 - -0.002) | <0.001 | -0.003  (-0.004 - -0.002) | <0.001 |
| Current health expenditure |  |  |  |  | -0.823  (-2.21 – 0.566) | 0.24 | -0.825  (-2.20 – 0.550) | 0.24 | -0.823  (-2.22 – 0.571) | 0.24 | -0.622  (-1.95 – 0.703) | 0.35 |
| Control of corruption |  |  |  |  |  |  | 0.161  (-6.18 – 6.50) | 0.96 |  |  |  |  |
| Absence of political instability and violence |  |  |  |  |  |  |  |  | 0.038  (-3.07 – 3.15) | 0.98 |  |  |
| Trade |  |  |  |  |  |  |  |  |  |  | -0.039  (-0.166 – 0.088) | 0.55 |
| AIC | 1498.85 | | 1402.80 | | 1079.54 | | 1081.53 | | 1081.54 | | 1042.49 | |
| BIC | 1502.32 | | 1409.69 | | 1089.25 | | 1094.48 | | 1094.48 | | 1055.33 | |

**final model

**Table B4.4 Fixed-effect regressions of acute respiratory infection treatment in children on the poverty headcount ratio at $1.90-per-day.**

|  | Baseline model | | + GDP | | + Current health expenditure | | + Control of corruption | | + Absence of political instability and violence | | + Trade** | |
| --- | --- | --- | --- | --- | --- | --- | --- | --- | --- | --- | --- | --- |
|  | Coefficient (95% CI) | p-value | Coefficient (95% CI) | p-value | Coefficient (95% CI) | p-value | Coefficient (95% CI) | p-value | Coefficient (95% CI) | p-value | Coefficient (95% CI) | p-value |
| ARI treatment | -0.265  (-0.506 - -0.023) | 0.03 | -0.161  (-0.406 – 0.084) | 0.20 | -0.211  (-0.481 – 0.059) | 0.12 | -0.210  (-0.477 – 0.057) | 0.12 | -0.208  (-0.479 – 0.064) | 0.13 | -0.188  (-0.452 – 0.077) | 0.16 |
| GDP per capita |  |  | -0.003  (-0.004 - -0.001) | <0.001 | -0.002  (-0.003 - -0.001) | <0.001 | -0.003  (-0.004 - -0.001) | <0.001 | -0.002  (-0.003 - -0.001) | <0.001 | -0.002  (-0.003 - -0.001) | <0.001 |
| Current health expenditure |  |  |  |  | -0.791  (-2.76 – 1.18) | 0.43 | 0.789  (-2.75 – 1.17) | 0.42 | -0.808  (-2.82 – 1.20) | 0.43 | -0.497  (-2.43 – 1.43) | 0.61 |
| Control of corruption |  |  |  |  |  |  | -0.143  (-9.11 – 8.83) | 0.98 |  |  |  |  |
| Absence of political instability and violence |  |  |  |  |  |  |  |  | -1.68  (-6.87 – 3.50) | 0.52 |  |  |
| Trade |  |  |  |  |  |  |  |  |  |  | -0.009  (-0.191 – 0.172) | 0.92 |
| AIC | 1639.51 | | 1538.56 | | 1198.45 | | 1200.45 | | 1199.19 | | 1161.78 | |
| BIC | 1642.97 | | 1590.45 | | 1208.16 | | 1213.39 | | 1212.13 | | 1174.62 | |

**final model

**Table B4.5 Fixed-effect regressions of acute respiratory infection treatment in children on the poverty headcount ratio at $3.20-per-day**

|  | **Baseline model** | | **+ GDP** | | **+ Current health expenditure** | | **+ Control of corruption** | | **+ Absence of political instability and violence** | | **+ Trade**** | |
| --- | --- | --- | --- | --- | --- | --- | --- | --- | --- | --- | --- | --- |
|  | Coefficient (95% CI) | p-value | Coefficient (95% CI) | p-value | Coefficient (95% CI) | p-value | Coefficient (95% CI) | p-value | Coefficient (95% CI) | p-value | Coefficient (95% CI) | p-value |
| **ARI treatment** | -0.265  (-0.466 – 0.064) | 0.01 | -0.111  (-0.280 – 0.059) | 0.20 | -0.128  (-0.311 – 0.056) | 0.17 | -0.131  (-0.310 – 0.049) | 0.15 | -0.128  (-0.310 – 0.053) | 0.16 | -0.101  (-0.282 – 0.079) | 0.27 |
| **GDP per capita** |  |  | -0.004  (-0.006 - -0.002) | <0.001 | -0.004  (-0.006 - -0.002) | <0.001 | -0.004  (-0.006 - -0.002) | <0.001 | -0.004  (-0.006 - -0.002) | <0.001 | -0.004  (-0.006 - -0.002) | <0.001 |
| **Current health expenditure** |  |  |  |  | -0.980  (-2.799 – 0.839) | 0.29 | -0.985  (-2.78 – 0.812) | 0.28 | -0.976  (-2.79 – 0.839) | 0.29 | -0.756  (-2.53 – 1.02) | 0.40 |
| **Control of corruption** |  |  |  |  |  |  | 0.495  (-8.01 – 8.99) | 0.91 |  |  |  |  |
| **Absence of political instability and violence** |  |  |  |  |  |  |  |  | 0.334  (-3.63 – 4.29) | 0.87 |  |  |
| **Trade** |  |  |  |  |  |  |  |  |  |  | -0.051  (-0.209 – 0.107) | 0.52 |
| **AIC** | 1638.58 | | 1535.21 | | 1192.26 | | 1194.24 | | 1194.21 | | 1156.30 | |
| **BIC** | 1642.04 | | 1542.09 | | 1201.97 | | 1207.18 | | 1207.16 | | 1169.13 | |

**final model

**Table B4.6 Fixed-effect regressions of acute respiratory infection treatment in children on the poverty headcount ratio at $5.50-per-day**

|  | **Baseline model** | | **+ GDP** | | **+ Current health expenditure** | | **+ Control of corruption** | | **+ Absence of political instability and violence** | | **+ Trade**** | |
| --- | --- | --- | --- | --- | --- | --- | --- | --- | --- | --- | --- | --- |
|  | Coefficient (95% CI) | p-value | Coefficient (95% CI) | p-value | Coefficient (95% CI) | p-value | Coefficient (95% CI) | p-value | Coefficient (95% CI) | p-value | Coefficient (95% CI) | p-value |
| **ARI treatment** | -0.186  (-0.336 – -0.036) | 0.02 | -0.014  (-0.131 – 0.1030 | 0.81 | -0.007  (-0.134 – 0.120) | 0.91 | -0.017  (-0.139 – 0.106) | 0.79 | -0.010  (-0.133 – 0.113) | 0.87 | 0.019  (-0.106 – 0.145) | 0.76 |
| **GDP per capita** |  |  | -0.005  (-0.007 - -0.003) | <0.001 | -0.005  (-0.007 - -0.003) | <0.001 | -0.005  (-0.007 - -0.003) | <0.001 | -0.005  (-0.007 - -0.003) | <0.001 | -0.005  (-0.007 - -0.003) | <0.001 |
| **Current health expenditure** |  |  |  |  | -0.747  (-2.09 – 0.594) | 0.27 | -0.765  (-2.09 – 0.564) | 0.26 | 0.731  (-2.05 – 0.588) | 0.28 | -0.647  (-1.92 – 0.627) | 0.32 |
| **Control of corruption** |  |  |  |  |  |  | 1.74  (-2.09 – 8.95) | 0.63 |  |  |  |  |
| **Absence of political instability and violence** |  |  |  |  |  |  |  |  | 1.58  (-1.33 – 4.48) | 0.28 |  |  |
| **Trade** |  |  |  |  |  |  |  |  |  |  | -0.072  (-0.187 – 0.044) | 0.22 |
| **AIC** | 1569.34 | | 1402.85 | | 1126.43 | | 1127.99 | | 1126.81 | | 1076.87 | |
| **BIC** | 1572.81 | | 1409.73 | | 1134.14 | | 1140.93 | | 1139.76 | | 1089.70 | |

**final model

# Tables B5: Fixed-effect regressions of full immunization rates and poverty

**Table B5.1 Fixed-effect regressions of full immunization rates in children on the poverty gap at $1.90-per-day**

|  | **Baseline model** | | **+ GDP** | | **+ Current health expenditure** | | **+ Control of corruption** | | **+ Absence of political instability and violence** | | **+ Trade**** | |
| --- | --- | --- | --- | --- | --- | --- | --- | --- | --- | --- | --- | --- |
|  | Coefficient (95% CI) | p-value | Coefficient (95% CI) | p-value | Coefficient (95% CI) | p-value | Coefficient (95% CI) | p-value | Coefficient (95% CI) | p-value | Coefficient (95% CI) | p-value |
| **Full immunization rates** | -0.202  (-0.307 – -0.096) | <0.001 | -0.182  (-0.296 - -0.067) | 0.002 | -0.172  (-0.298 - -0.046) | 0.008 | -0.175  (-0.299 - -0.050) | 0.006 | -0.176  (-0.301 - -0.051) | 0.006 | -0.191  (-0.323 - -0.060) | 0.005 |
| **GDP per capita** |  |  | -0.0001  (-0.001 – 0.0003) | 0.63 | -0.0002  (-0.001 – 0.0003) | 0.36 | -0.0002  (-0.001 – 0.0003) | 0.43 | -0.0001  (-0.001 – 0.0004) | 0.63 | -0.0001  (-0.001 – 0.0005) | 0.85 |
| **Current health expenditure** |  |  |  |  | -0.794  (-1.69 – 0.103) | 0.08 | -0.729  (-1.60 – 0.143) | 0.10 | -0.837  (-1.74 – 0.068) | 0.07 | -0.609  (-1.41 – 0.192) | 0.13 |
| **Control of corruption** |  |  |  |  |  |  | -2.46  (-7.97 – 3.04) | 0.38 |  |  |  |  |
| **Absence of political instability and violence** |  |  |  |  |  |  |  |  | -1.59  (-4.11 – 0.921) | 0.21 | -1.80  (-4.33 – 0.724) | 0.16 |
| **Trade** |  |  |  |  |  |  |  |  |  |  | -0.009  (-0.082 – 0.065) | 0.82 |
| **AIC** | 1491.74 | | 1452.69 | | 1081.30 | | 1081.30 | | 1078.99 | | 1033.01 | |
| **BIC** | 1495.31 | | 1459.79 | | 1091.30 | | 1094.63 | | 1092.32 | | 1049.55 | |

**final model

**Table B5.2 Fixed-effect regressions of full immunization rates in children on the poverty gap at $3.20-per-day**

|  | **Baseline model** | | **+ GDP** | | **+ Current health expenditure** | | **+ Control of corruption** | | **+ Absence of political instability and violence** | | **+ Trade**** | |
| --- | --- | --- | --- | --- | --- | --- | --- | --- | --- | --- | --- | --- |
|  | Coefficient (95% CI) | p-value | Coefficient (95% CI) | p-value | Coefficient (95% CI) | p-value | Coefficient (95% CI) | p-value | Coefficient (95% CI) | p-value | Coefficient (95% CI) | p-value |
| **Full immunization rates** | -0.291  (-0.417 - -0.166) | <0.001 | -0.227  (-0.358 - -0.097) | 0.001 | -0.212  (-0.354 - -0.070) | 0.004 | -0.214  (-0.354 - -0.074) | 0.003 | -0.216  (-0.356 - -0.075) | 0.003 | -0.229  (-0.381 - -0.077) | 0.004 |
| **GDP per capita** |  |  | -0.001  (-0.002 - -0.0001) | 0.04 | -0.001  (-0.002 - -0.0005) | 0.002 | -0.001  (-0.002 - -0.0005) | 0.002 | -0.001  (-0.002 - -0.0004) | 0.005 | -0.001  (-0.002 - -0.0004) | 0.008 |
| **Current health expenditure** |  |  |  |  | -1.11  (-2.27 – 0.044) | 0.06 | -1.06  (-2.20 – 0.082) | 0.07 | -1.16  (-2.33 – 0.016) | 0.05 | -0.861  (-1.93 – 0.207) | 0.11 |
| **Control of corruption** |  |  |  |  |  |  | -1.94  (-7.92 – 4.04) | 0.52 |  |  |  |  |
| **Absence of political instability and violence** |  |  |  |  |  |  |  |  | -1.66  (-4.34 – 1.02) | 0.22 |  |  |
| **Trade** |  |  |  |  |  |  |  |  |  |  | -0.010  (-0.107 – 0.086) | 0.83 |
| **AIC** | 1611.54 | | 1562.58 | | 1169.88 | | 1171.07 | | 1168.83 | | 1128.98 | |
| **BIC** | 1615.12 | | 1569.68 | | 1179.87 | | 1184.40 | | 1182.16 | | 1142.22 | |

**final model

**Table B5.3 Fixed-effect regressions of full immunization rates in children on the poverty gap at $5.50-per-day**

|  | **Baseline model** | | **+ GDP** | | **+ Current health expenditure** | | **+ Control of corruption** | | **+ Absence of political instability and violence** | | **+ Trade**** | |
| --- | --- | --- | --- | --- | --- | --- | --- | --- | --- | --- | --- | --- |
|  | Coefficient (95% CI) | p-value | Coefficient (95% CI) | p-value | Coefficient (95% CI) | p-value | Coefficient (95% CI) | p-value | Coefficient (95% CI) | p-value | Coefficient (95% CI) | p-value |
| **Full immunization rates** | -0.343  (-0.461 - -0.224) | <0.001 | -0.210  (-0.331 – -0.089) | 0.001 | -0.195  (-0.330 - -0.060) | 0.005 | -0.195  (-0.329 - -0.062) | 0.005 | -0.196  (-0.330 - -0.062) | 0.005 | -0.218  (-0.362 - -0.073) | 0.004 |
| **GDP per capita** |  |  | -0.002  (-0.003 - -0.001) | <0.001 | -0.002  (-0.003 - -0.001) | <0.001 | -0.002  (-0.003 - -0.001) | <0.001 | -0.002  (-0.003 - -0.001) | <0.001 | -0.003  (-0.004 - -0.001) | <0.001 |
| **Current health expenditure** |  |  |  |  | -1.38  (-2.72 - -0.047) | 0.04 | -1.38  (-2.73 - -0.026) | 0.05 | -1.40  (-2.75 - -0.052) | 0.04 | -1.17  (-2.47 - -0.129) | 0.08 |
| **Control of corruption** |  |  |  |  |  |  | -0.240  (-6.60 – 6.12) | 0.94 |  |  |  |  |
| **Absence of political instability and violence** |  |  |  |  |  |  |  |  | -0.588  (-3.06 – 1.88) | 0.64 |  |  |
| **Trade** |  |  |  |  |  |  |  |  |  |  | -0.0126  (-0.125 – 0.099) | 0.82 |
| **AIC** | 1671.86 | | 1588.83 | | 1217.44 | | 1219.43 | | 1219.14 | | 1180.55 | |
| **BIC** | 1675.43 | | 1595.93 | | 1227.44 | | 1232.76 | | 1232.47 | | 1193.79 | |

**final model

**Table B5.4 Fixed-effect regressions of full immunization rates in children on the poverty headcount ratio at $1.90-per-day**

|  | **Baseline model** | | **+ GDP** | | **+ Current health expenditure** | | **+ Control of corruption** | | **+ Absence of political instability and violence** | | **+ Trade**** | |
| --- | --- | --- | --- | --- | --- | --- | --- | --- | --- | --- | --- | --- |
|  | Coefficient (95% CI) | p-value | Coefficient (95% CI) | p-value | Coefficient (95% CI) | p-value | Coefficient (95% CI) | p-value | Coefficient (95% CI) | p-value | Coefficient (95% CI) | p-value |
| **Full immunization rates** | -0.386  (-0.564 - -0.208) | <0.001 | -0.308  (-0.490 - -0.127) | 0.001 | -0.289  (-0.486 – -0.091) | 0.005 | -0.291  (-0.486 - -0.095) | 0.004 | -0.295  (-0.490 - -0.099) | 0.004 | -0.316  (-0.524 - -0.109) | 0.003 |
| **GDP per capita** |  |  | -0.001  (-0.002 – 0.0001) | 0.08 | -0.002  (-0.003 – -0.001) | 0.004 | -0.002  (-0.003 - -0.0005) | 0.004 | -0.001  (-0.003 - -0.0003) | 0.01 | -0.001  (-0.004 - -0.00004) | 0.04 |
| **Current health expenditure** |  |  |  |  | -1.30  (-2.90 – 0.298) | 0.11 | -1.25  (-2.82 – 0.329) | 0.12 | -1.38  (-3.00 – 0.249) | 0.09 | -1.05  (-2.58 – 0.484) | 0.18 |
| **Control of corruption** |  |  |  |  |  |  | -2.12  (-10.4 – 6.15) | 0.61 |  |  |  |  |
| **Absence of political instability and violence** |  |  |  |  |  |  |  |  | -2.71  (-6.68 – 1.27) | 0.18 | -2.95  (-6.87 – 0.965) | 0.14 |
| **Trade** |  |  |  |  |  |  |  |  |  |  | -0.004  (-0.135 – 0.127) | 0.95 |
| **AIC** | 1795.86 | | 1746.94 | | 1309.21 | | 1310.72 | | 1307.08 | | 1265.15 | |
| **BIC** | 1799.43 | | 1754.04 | | 1319.21 | | 1324.05 | | 1320.41 | | 1281.70 | |

**final model

**Table B5.5 Fixed-effect regressions of full immunization rates in children on the poverty headcount ratio at $3.20-per-day**

|  | **Baseline model** | | **+ GDP** | | **+ Current health expenditure** | | **+ Control of corruption** | | **+ Absence of political instability and violence** | | **+ Trade**** | |
| --- | --- | --- | --- | --- | --- | --- | --- | --- | --- | --- | --- | --- |
|  | Coefficient (95% CI) | p-value | Coefficient (95% CI) | p-value | Coefficient (95% CI) | p-value | Coefficient (95% CI) | p-value | Coefficient (95% CI) | p-value | Coefficient (95% CI) | p-value |
| **Full immunization rates** | -0.434  (-0.600 – -0.268) | <0.001 | -0.256  (-0.418 - -0.094) | 0.002 | -0.234  (-0.415 - -0.053) | 0.01 | -0.234  (-0.414 - -0.053) | 0.01 | -0.236  (-0.416 - -0.055) | 0.01 | -0.255  (-0.452 - -0.059) | 0.01 |
| **GDP per capita** |  |  | -0.003  (-0.004 - -0.001) | <0.001 | -0.004  (-0.005 - -0.002) | <0.001 | -0.004  (-0.005 - -0.002) | <0.001 | -0.004  (-0.005 - -0.002) | <0.001 | -0.004  (-0.006 - -0.002) | <0.001 |
| **Current health expenditure** |  |  |  |  | -1.81  (-3.71 – 0.088) | 0.06 | -1.82  (-3.75 – 0.106) | 0.06 | -1.83  (-3.74 – 0.081) | 0.06 | -1.58  (-3.48 – 0.315) | 0.10 |
| **Control of corruption** |  |  |  |  |  |  | 0.290  (-8.67 – 9.25) | 0.95 |  |  |  |  |
| **Absence of political instability and violence** |  |  |  |  |  |  |  |  | -0.692  (-4.18 – 2.79) | 0.69 |  |  |
| **Trade** |  |  |  |  |  |  |  |  |  |  | -0.015  (-0.179 – 0.149) | 0.86 |
| **AIC** | 1843.83 | | 1757.35 | | 1360.89 | | 1362.89 | | 1362.69 | | 1326.61 | |
| **BIC** | 1847.40 | | 1764.45 | | 1370.89 | | 1376.22 | | 1376.02 | | 1339.84 | |

**final model

**Table B5.6 Fixed-effect regressions of full immunization rates in children on the poverty headcount ratio at $5.50-per-day.**

|  | **Baseline model** | | **+ GDP** | | **+ Current health expenditure** | | **+ Control of corruption** | | **+ Absence of political instability and violence** | | **+ Trade**** | |
| --- | --- | --- | --- | --- | --- | --- | --- | --- | --- | --- | --- | --- |
|  | Coefficient (95% CI) | p-value | Coefficient (95% CI) | p-value | Coefficient (95% CI) | p-value | Coefficient (95% CI) | p-value | Coefficient (95% CI) | p-value | Coefficient (95% CI) | p-value |
| **Full immunization rates** | -0.375  (-0.507 - -0.243) | <0.001 | -0.119  (-0.242 – 0.005) | 0.06 | -0.110  (-0.255 – 0.035) | 0.13 | -0.107  (-0.252 – 0.037) | 0.14 | -0.106  (-0.253 – 0.040) | 0.15 | -0.149  (-0.289 - -0.009) | 0.04 |
| **GDP per capita** |  |  | -0.004  (-0.005 - -0.003) | <0.001 | -0.005  (-0.007 - -0.004) | <0.001 | -0.005  (-0.007 - -0.004) | <0.001 | -0.005  (-0.007 - -0.004) | <0.001 | -0.005  (-0.007 - -0.004) | <0.001 |
| **Current health expenditure** |  |  |  |  | -1.46  (-3.05 – 0.142) | 0.07 | -1.53  (-3.18 – 0.118) | 0.07 | -1.41  (-2.98 – 0.163) | 0.08 | -1.36  (-2.99 – 0.270) | 0.10 |
| **Control of corruption** |  |  |  |  |  |  | 2.79  (-3.18 – 0.118) | 0.49 |  |  |  |  |
| **Absence of political instability and violence** |  |  |  |  |  |  |  |  | 1.76  (-0.865 – 4.38) | 0.19 |  |  |
| **Trade** |  |  |  |  |  |  |  |  |  |  | -0.017  (-0.156 – 0.121) | 0.81 |
| **AIC** | 1812.80 | | 1645.86 | | 1306.68 | | 1307.82 | | 1306.93 | | 1267.39 | |
| **BIC** | 1816.37 | | 1652.96 | | 1316.68 | | 1321.15 | | 1320.26 | | 1280.62 | |

**final model

# Tables B6 Fixed-effect regressions of antenatal care on poverty

**Table B6.1 Fixed-effect regressions of antenatal care on the poverty gap at $1.90-per-day.**

|  | **Baseline model** | | **+ GDP** | | **+ Current health expenditure** | | **+ Control of corruption** | | **+ Absence of political instability and violence** | | **+ Trade**** | |
| --- | --- | --- | --- | --- | --- | --- | --- | --- | --- | --- | --- | --- |
|  | Coefficient (95% CI) | p-value | Coefficient (95% CI) | p-value | Coefficient (95% CI) | p-value | Coefficient (95% CI) | p-value | Coefficient (95% CI) | p-value | Coefficient (95% CI) | p-value |
| **Antenatal care** | -0.178  (-0.259 – -0.097) | <0.001 | -0.161  (-0.270 – -0.051) | 0.005 | -0.211  (-0.318 – -0.104) | <0.001 | -0.211  (-0.318 – -0.104) | <0.001 | -0.210  (-0.307 – -0.114) | <0.001 | -0.231  (-0.330 – -0.131) | <0.001 |
| **GDP per capita** |  |  | -0.0002  (-0.001 - 0.0003) | 0.36 | -0.0002  (-0.001 - 0.0003) | 0.47 | -0.0002  (-0.001 - 0.0003) | 0.47 | 0.00001  (-0.001 - 0.001) | 0.97 | 0.00001  (-0.0004 - 0.001) | 0.83 |
| **Current health expenditure** |  |  |  |  | -0.521  (-1.52 – 0.474) | 0.30 | -0.521  (-1.52 – 0.476) | 0.30 | -0.574  (-1.52 – 0.369) | 0.23 | -0.211  (-1.09 – 0.671) | 0.64 |
| **Control of corruption** |  |  |  |  |  |  | -0.011  (-4.81 – 4.78) | 0.99 |  |  |  |  |
| **Absence of political instability and violence** |  |  |  |  |  |  |  |  | -1.76  (-4.23 – 0.722) | 0.16 | -1.70  (-4.22 – 0.820) | 0.18 |
| **Trade** |  |  |  |  |  |  |  |  |  |  | 0.047  (-0.012 – 0.107) | 0.12 |
| **AIC** | 1318.81 | | 1295.34 | | 896.05 | | 898.05 | | 891.03 | | 824.72 | |
| **BIC** | 1322.29 | | 1302.25 | | 905.73 | | 910.95 | | 903.93 | | 840.71 | |

**final model

**Table B6.2 Fixed-effect regressions of antenatal care on the poverty gap at $3.20-per-day.**

|  | **Baseline model** | | **+ GDP** | | **+ Current health expenditure** | | **+ Control of corruption** | | **+ Absence of political instability and violence** | | **+ Trade**** | |
| --- | --- | --- | --- | --- | --- | --- | --- | --- | --- | --- | --- | --- |
|  | Coefficient (95% CI) | p-value | Coefficient (95% CI) | p-value | Coefficient (95% CI) | p-value | Coefficient (95% CI) | p-value | Coefficient (95% CI) | p-value | Coefficient (95% CI) | p-value |
| **Antenatal care** | -0.345  (-0.452 – -0.238) | <0.001 | -0.296  (-0.432 – -0.159) | <0.001 | -0.337  (-0.462 – -0.212) | <0.001 | -0.342  (-0.468 – -0.215) | <0.001 | -0.336  (-0.462– -0.211) | <0.001 | -0.357  (-0.494– -0.221) | <0.001 |
| **GDP per capita** |  |  | -0.001  (-0.001 – 0.00002) | 0.06 | -0.001  (-0.001 – -0.0004) | 0.001 | -0.001  (-0.001 – -0.0003) | 0.002 | -0.001  (-0.001 – -0.0002) | 0.01 | -0.001  (-0.001 – -0.0002) | 0.002 |
| **Current health expenditure** |  |  |  |  | -0.522  (-1.66 – 0.621) | 0.37 | -0.552  (-1.72 – 0.614) | 0.35 | -0.558  (-1.67 – 0.559) | 0.32 | -0.141  (-1.23 – 0.953) | 0.78 |
| **Control of corruption** |  |  |  |  |  |  | 1.09  (-2.89 – 5.07) | 0.59 |  |  |  |  |
| **Absence of political instability and violence** |  |  |  |  |  |  |  |  | -1.18  (-3.46 – 1.10) | 0.31 |  |  |
| **Trade** |  |  |  |  |  |  |  |  |  |  | 0.046  (-0.032 – 0.124) | 0.25 |
| **AIC** | 1404.15 | | 1373.49 | | 959.26 | | 960.93 | | 959.03 | | 903.42 | |
| **BIC** | 1407.63 | | 1380.41 | | 968.94 | | 973.84 | | 971.93 | | 916.21 | |

**final model

**Table B6.3 Fixed-effect regressions of antenatal care on the poverty gap at $5.50-per-day.**

|  | **Baseline model** | | **+ GDP** | | **+ Current health expenditure** | | **+ Control of corruption** | | **+ Absence of political instability and violence** | | **+ Trade**** | |
| --- | --- | --- | --- | --- | --- | --- | --- | --- | --- | --- | --- | --- |
|  | Coefficient (95% CI) | p-value | Coefficient (95% CI) | p-value | Coefficient (95% CI) | p-value | Coefficient (95% CI) | p-value | Coefficient (95% CI) | p-value | Coefficient (95% CI) | p-value |
| **Antenatal care** | -0.477  (-0.608 - -0.0346) | <0.001 | -0.353  (-0.516 – -0.190) | <0.001 | -0.390  (-0.566 - -0.214) | <0.001 | -0.403  (-0.577 – -0.229) | <0.001 | -0.390  (-0.565 - -0.214) | <0.001 | -0.411  (-0.592 - -0.229) | <0.001 |
| **GDP per capita** |  |  | -0.002  (-0.002 - -0.001) | <0.001 | -0.002  (-0.003 - -0.001) | <0.001 | -0.002  (-0.003 - -0.001) | <0.001 | -0.002  (-0.003 - -0.001) | <0.001 | -0.002  (-0.003 - -0.001) | <0.001 |
| **Current health expenditure** |  |  |  |  | -0.684  (-1.74 – 0.373) | 0.20 | -0.763  (-1.86 – 0.333) | 0.17 | -0.675  (-1.73 – 0.385) | 0.21 | -0.382  (-1.37 – 0.606) | 0.46 |
| **Control of corruption** |  |  |  |  |  |  | 2.85  (-2.08 – 7.79) | 0.25 |  |  |  |  |
| **Absence of political instability and violence** |  |  |  |  |  |  |  |  | 0.320  (-1.53 – 2.17) | 0.73 |  |  |
| **Trade** |  |  |  |  |  |  |  |  |  |  | 0.026  (-0.057 – 0.109) | 0.52 |
| **AIC** | 1422.76 | | 1362.06 | | 975.07 | | 975.00 | | 976.92 | | 928.38 | |
| **BIC** | 1426.24 | | 1368.97 | | 984.74 | | 987.90 | | 989.82 | | 941.17 | |

**final model

**Table B6.4 Fixed-effect regressions of antenatal care on the poverty headcount ratio at $1.90-per-day.**

|  | **Baseline model** | | **+ GDP** | | **+ Current health expenditure** | | **+ Control of corruption** | | **+ Absence of political instability and violence** | | **+ Trade**** | |
| --- | --- | --- | --- | --- | --- | --- | --- | --- | --- | --- | --- | --- |
|  | Coefficient (95% CI) | p-value | Coefficient (95% CI) | p-value | Coefficient (95% CI) | p-value | Coefficient (95% CI) | p-value | Coefficient (95% CI) | p-value | Coefficient (95% CI) | p-value |
| **Antenatal care** | -0.455  (-0.614 - -0.294) | <0.001 | -0.405  (-0.601 – -0.209) | <0.001 | -0.457  (-0.646 – -0.268) | <0.001 | -0.463  (-0.653 - -0.273) | <0.001 | -0.466  (-0.645 - -0.263) | <0.001 | -0.483  (-0.6492- -0.276) | <0.001 |
| **GDP per capita** |  |  | -0.001  (-0.002 – 0.0002) | 0.14 | -0.001  (-0.002 - -0.0003) | 0.007 | -0.001  (-0.002 - -0.0002) | 0.01 | -0.001  (-0.002 - 0.0001) | 0.07 | -0.001  (-0.002 - -0.0002) | 0.01 |
| **Current health expenditure** |  |  |  |  | -0.470  (-2.13 – 1.19) | 0.58 | -0.508  (-2.22 – 1.20) | 0.56 | -0.533  (-2.15 – 1.08) | 0.51 | -0.042  (-1.62 – 1.70) | 0.96 |
| **Control of corruption** |  |  |  |  |  |  | 1.36  (-4.27 – 6.98) | 0.63 |  |  |  |  |
| **Absence of political instability and violence** |  |  |  |  |  |  |  |  | -2.09  (-5.65 – 1.47) | 0.25 |  |  |
| **Trade** |  |  |  |  |  |  |  |  |  |  | 0.068  (-0.056 – 0.192) | 0.28 |
| **AIC** | 1584.86 | | 1553.42 | | 1106.34 | | 1108.11 | | 1105.15 | | 1054.54 | |
| **BIC** | 1588.33 | | 1560.33 | | 1116.01 | | 1121.01 | | 1118.06 | | 1067.33 | |

**final model

**Table B6.5 Fixed-effect regressions of antenatal care on the poverty headcount ratio at $3.20-per-day.**

|  | **Baseline model** | | **+ GDP** | | **+ Current health expenditure** | | **+ Control of corruption** | | **+ Absence of political instability and violence** | | **+ Trade**** | |
| --- | --- | --- | --- | --- | --- | --- | --- | --- | --- | --- | --- | --- |
|  | Coefficient (95% CI) | p-value | Coefficient (95% CI) | p-value | Coefficient (95% CI) | p-value | Coefficient (95% CI) | p-value | Coefficient (95% CI) | p-value | Coefficient (95% CI) | p-value |
| **Antenatal care** | -0.671  (-0.871 - -0.471) | <0.001 | -0.521  (-0.765 - -0.276) | <0.001 | -0.543  (-0.829 - -0.258) | <0.001 | -0.562  (-0.837 - -0.288) | <0.001 | -0.544  (-0.824 - -0.263) | <0.001 | -0.559  (-0.848 - -0.271) | <0.001 |
| **GDP per capita** |  |  | -0.002  (-0.003 – -0.001) | <0.001 | -0.003  (-0.004 - -0.002) | <0.001 | -0.003  (-0.004 - -0.002) | <0.001 | -0.003  (-0.004 - -0.002) | <0.001 | -0.003  (-0.004 - -0.002) | <0.001 |
| **Current health expenditure** |  |  |  |  | -0.723  (-2.09 – 0.641) | 0.30 | -0.838  (-2.26 – 0.580) | 0.24 | -0.689  (-2.06 – 0.680) | 0.32 | -0.412  (-1.73 – 0.905) | 0.54 |
| **Control of corruption** |  |  |  |  |  |  | 4.19  (-3.89 – 12.3) | 0.31 |  |  |  |  |
| **Absence of political instability and violence** |  |  |  |  |  |  |  |  | 1.14  (-1.50 – 3.78) | 0.39 |  |  |
| **Trade** |  |  |  |  |  |  |  |  |  |  | 0.013  (-0.103 – 0.130) | 0.82 |
| **AIC** | 1551.12 | | 1493.04 | | 1096.62 | | 1096.30 | | 1097.63 | | 1058.32 | |
| **BIC** | 1554.60 | | 1499.95 | | 1106.30 | | 1109.20 | | 1110.54 | | 1071.11 | |

**final model

**Table B6.6 Fixed-effect regressions of antenatal care on the poverty headcount ratio at $5.50-per-day.**

|  | **Baseline model** | | **+ GDP** | | **+ Current health expenditure** | | **+ Control of corruption** | | **+ Absence of political instability and violence** | | **+ Trade**** | |
| --- | --- | --- | --- | --- | --- | --- | --- | --- | --- | --- | --- | --- |
|  | Coefficient (95% CI) | p-value | Coefficient (95% CI) | p-value | Coefficient (95% CI) | p-value | Coefficient (95% CI) | p-value | Coefficient (95% CI) | p-value | Coefficient (95% CI) | p-value |
| **Antenatal care** | -0.608  (-0.796 - -0.419) | <0.001 | -0.325  (-0.540 – -0.109) | 0.004 | -0.349  (-0.631 – -0.067) | <0.001 | -0.373  (-0.652 - -0.095) | <0.001 | -0.351  (-0.620 - -0.082) | <0.001 | -0.381  (-0.642 - -0.119) | 0.005 |
| **GDP per capita** |  |  | -0.004  (-0.005 - -0.003) | <0.001 | -0.004  (-0.006 - -0.003) | <0.001 | -0.004  (-0.006 - -0.003) | <0.001 | -0.005  (-0.006 - -0.003) | <0.001 | -0.004  (-0.006 - -0.003) | <0.001 |
| **Current health expenditure** |  |  |  |  | -0.952  (-1.98 - 0.077) | 0.07 | -1.10  (-2.18 - -0.012) | 0.05 | -0.865  (-1.89 – 0.161) | 0.09 | -0.784  (-1.78 – 0.212) | 0.12 |
| **Control of corruption** |  |  |  |  |  |  | 5.31  (-3.99 – 14.6) | 0.26 |  |  |  |  |
| **Absence of political instability and violence** |  |  |  |  |  |  |  |  | 2.86  (0.055 – 5.66) | 0.05 | 2.86  (-0.076 – 5.80) | 0.06 |
| **Trade** |  |  |  |  |  |  |  |  |  |  | -0.001  (-0.114 – 0.112) | 0.98 |
| **AIC** | 1559.52 | | 1414.93 | | 1083.39 | | 1081.37 | | 1078.61 | | 1031.48 | |
| **BIC** | 1562.99 | | 1421.85 | | 1093.07 | | 1094.27 | | 1091.52 | | 1047.47 | |

**final model

# Tables B7: Fixed-effect regressions of breast cancer screening and on the poverty

**Table B7.1 Fixed-effect regressions of breast cancer screening on the poverty gap at $1.90-per-day.**

|  | Baseline model | | + GDP | | + Current health expenditure ** | | + Control of corruption | | + Absence of political instability and violence | | + Trade | |
| --- | --- | --- | --- | --- | --- | --- | --- | --- | --- | --- | --- | --- |
|  | Coefficient (95% CI) | p-value | Coefficient (95% CI) | p-value | Coefficient (95% CI) | p-value | Coefficient (95% CI) | p-value | Coefficient (95% CI) | p-value | Coefficient (95% CI) | p-value |
| Breast cancer screening | -0.044  (-0.067 - -0.021) | <0.001 | -0.029  (-0.055 - -0.003) | 0.03 | -0.015  (-0.041 – 0.012) | 0.26 | 0.002  (-0.045 – 0.049) | 0.94 | -0.008  (-0.054 – 0.037) | 0.71 | -0.017  (-0.042 – 0.008) | 0.18 |
| GDP per capita |  |  | -0.0002  (-0.0004 – 0.000007) | 0.06 | -0.0002  (-0.0005 – 0.000007) | 0.06 | -0.0002  (-0.0005 – 0.000008) | 0.06 | -0.0002  (-0.0006 – 0.00005) | 0.10 | -0.0002  (-0.0005 – 0.00003) | 0.08 |
| Current health expenditure |  |  |  |  | -0.555  (-1.65 – 0.544) | 0.31 | -0.644  (-1.72 – 0.442) | 0.24 | -0.636  (-1.89 – 0.614) | 0.31 | -0.511  (-1.45 – 0.427) | 0.28 |
| Control of corruption |  |  |  |  |  |  | 1.49  (-1.29 – 4.27) | 0.29 |  |  |  |  |
| Absence of political instability and violence |  |  |  |  |  |  |  |  | 0.579  (-1.34 – 2.50) | 0.55 |  |  |
| Trade |  |  |  |  |  |  |  |  |  |  | 0.008  (-0.049 – 0.066) | 0.77 |
| AIC | 174.85 | | 168.90 | | 136.56 | | 135.31 | | 137.90 | | 137.39 | |
| BIC | 177.15 | | 173.45 | | 143.17 | | 144.13 | | 146.71 | | 146.15 | |

**final model

**Table B7.2 Fixed-effect regressions of breast cancer screening on the poverty gap at $3.20-per-day.**

|  | Baseline model | | + GDP | | + Current health expenditure | | + Control of corruption | | + Absence of political instability and violence | | + Trade** | |
| --- | --- | --- | --- | --- | --- | --- | --- | --- | --- | --- | --- | --- |
|  | Coefficient (95% CI) | p-value | Coefficient (95% CI) | p-value | Coefficient (95% CI) | p-value | Coefficient (95% CI) | p-value | Coefficient (95% CI) | p-value | Coefficient (95% CI) | p-value |
| Breast cancer screening | -0.084  (-0.126 – -0.043) | <0.001 | -0.043  (-0.110 – 0.024) | 0.20 | -0.036  (-0.075 – 0.004) | 0.07 | 0.0002  (-0.078 – 0.079) | 0.99 | -0.003  (-0.087 – 0.082) | 0.95 | -0.004  (-0.078 – 0.086) | 0.92 |
| GDP per capita |  |  | -0.0006  (-0.001 – -0.00008) | 0.02 | -0.0006  (-0.001 – -0.0001) | 0.02 | -0.0006  (-0.001 – -0.0001) | 0.02 | -0.0006  (-0.001 - -0.00001) | 0.05 | -0.001  (-0.001 – 0.0001) | 0.02 |
| Current health expenditure |  |  |  |  | -0.802  (-2.28 – 0.680) | 0.28 | -0.993  (-2.40 – 0.409) | 0.16 | -0.942  (-2.63 – 0.749) | 0.27 | -1.08  (-2.44 – 0.274) | 0.12 |
| Control of corruption |  |  |  |  |  |  | 3.23  (-1.49 – 7.94) | 0.18 | 3.43  (-2.09 – 8.96) | 0.22 | 3.16  (-1.56 – 7.88) | 0.18 |
| Absence of political instability and violence |  |  |  |  |  |  |  |  | 0.455  (-4.49 – 3.58) | 0.82 |  |  |
| Trade |  |  |  |  |  |  |  |  |  |  | -0.018  (-0.097 – 0.062) | 0.65 |
| AIC | 244.17 | | 226.76 | | 193.52 | | 188.86 | | 190.70 | | 188.71 | |
| BIC | 246.47 | | 231.32 | | 200.14 | | 197.68 | | 201.72 | | 199.66 | |

**final model

**Table B7.3 Fixed-effect regressions of breast cancer screening on the poverty gap at $5.50-per-day.**

|  | **Baseline model** | | **+ GDP** | | **+ Current health expenditure** | | **+ Control of corruption** | | **+ Absence of political instability and violence** | | **+ Trade**** | |
| --- | --- | --- | --- | --- | --- | --- | --- | --- | --- | --- | --- | --- |
|  | Coefficient (95% CI) | p-value | Coefficient (95% CI) | p-value | Coefficient (95% CI) | p-value | Coefficient (95% CI) | p-value | Coefficient (95% CI) | p-value | Coefficient (95% CI) | p-value |
| **Breast cancer screening** | -0.132  (-0.222 – -0.042) | 0.005 | -0.034  (-0.189 – 0.120) | 0.66 | -0.045  (-0.124 – 0.034) | 0.25 | 0.008  (-0.099 – 0.116) | 0.88 | 0.009  (-0.105 – 0.122) | 0.88 | 0.027  (-0.008 – 0.139) | 0.62 |
| **GDP per capita** |  |  | -0.001  (-0.002 – -0.0005) | 0.002 | -0.001  (-0.002 – -0.0006) | 0.001 | -0.001  (-0.002 – -0.0006) | 0.001 | -0.001  (-0.002 - -0.0005) | 0.004 | -0.001  (-0.002 – 0.001) | <0.001 |
| **Current health expenditure** |  |  |  |  | -1.04  (3.17 – 1.10) | 0.33 | -1.32  (-3.24 – 0.599) | 0.17 | -1.33  (-3.44 – 0.777) | 0.21 | -1.76  (-3.58 – 0.062) | 0.06 |
| **Control of corruption** |  |  |  |  |  |  | 4.77  (-0.892 – 10.4) | 0.09 |  | 0.17 | 4.43  (-1.33 – 10.2) | 0.13 |
| **Absence of political instability and violence** |  |  |  |  |  |  |  |  | 0.086  (-6.46 – 6.63) | 0.98 |  |  |
| **Trade** |  |  |  |  |  |  |  |  |  |  | -0.089  (-0.185 – 0.007) | 0.07 |
| **AIC** | 351.27 | | 276.19 | | 230.40 | | 223.88 | | 225.87 | | 216.68 | |
| **BIC** | 317.58 | | 280.74 | | 237.01 | | 232.70 | | 236.90 | | 227.63 | |

**final model

**Table B7.4 Fixed-effect regressions of breast cancer screening on the poverty headcount ratio at $1.90-per-day.**

|  | **Baseline model** | | **+ GDP** | | **+ Current health expenditure** | | **+ Control of corruption**** | | **+ Absence of political instability and violence** | | **+ Trade** | |
| --- | --- | --- | --- | --- | --- | --- | --- | --- | --- | --- | --- | --- |
|  | Coefficient (95% CI) | p-value | Coefficient (95% CI) | p-value | Coefficient (95% CI) | p-value | Coefficient (95% CI) | p-value | Coefficient (95% CI) | p-value | Coefficient (95% CI) | p-value |
| **Breast cancer screening** | -0.104  (-0.152 – -0.056) | <0.001 | -0.060  (-0.141 – 0.021) | 0.15 | -0.047  (-0.099 – 0.005) | 0.08 | 0.008  (-0.099 – 0.115) | 0.88 | 0.005  (-0.111 – 0.120) | 0.94 | 0.012  (-0.100 – 0.125) | 0.83 |
| **GDP per capita** |  |  | -0.0006  (-0.001 – 0.000004) | 0.05 | -0.0007  (-0.001 – 0.000002) | 0.05 | -0.001  (-0.001 – 0.000003) | 0.05 | -0.007  (-0.002 – 0.0002) | 0.11 | -0.001  (-0.001 – 0.000001) | 0.05 |
| **Current health expenditure** |  |  |  |  | -1.07  (-2.99 – 0.846) | 0.27 | -1.36  (-3.18 – 0.453) | 0.14 | -1.30  (-3.49 – 0.900) | 0.24 | -1.45  (3.22 – 0.309) | 0.10 |
| **Control of corruption** |  |  |  |  |  |  | 4.95  (-1.60 – 11.5) | 0.14 | 5.23  (-2.31 – 12.8) | 0.17 | 4.88  (-1.64 – 11.4) | 0.14 |
| **Absence of political instability and violence** |  |  |  |  |  |  |  |  | -0.609  (-5.91 – 4.69) | 0.82 |  |  |
| **Trade** |  |  |  |  |  |  |  |  |  |  | -0.018  (-0.132 – 0.095) | 0.75 |
| **AIC** | 281.91 | | 267.60 | | 237.50 | | 231.28 | | 233.12 | | 230.69 | |
| **BIC** | 284.21 | | 272.15 | | 244.11 | | 240.09 | | 244.14 | | 241.64 | |

**final model

**Table B7.5 Fixed-effect regressions of breast cancer screening on the poverty headcount ratio at $3.20-per-day.**

|  | **Baseline model** | | **+ GDP** | | **+ Current health expenditure** | | **+ Control of corruption** | | **+ Absence of political instability and violence** | | **+ Trade**** | |
| --- | --- | --- | --- | --- | --- | --- | --- | --- | --- | --- | --- | --- |
|  | Coefficient (95% CI) | p-value | Coefficient (95% CI) | p-value | Coefficient (95% CI) | p-value | Coefficient (95% CI) | p-value | Coefficient (95% CI) | p-value | Coefficient (95% CI) | p-value |
| **Breast cancer screening** | -0.174  (-0.282 – -0.066) | 0.002 | -0.057  (-0.244 – 0.131) | 0.55 | -0.069  (-0.164 – 0.027) | 0.16 | -0.003  (-0.152 – 0.146) | 0.97 | -0.012  (-0.166 - 0.145) | 0.89 | 0.023  (-0.133 – 0.178) | 0.77 |
| **GDP per capita** |  |  | -0.002  (-0.003 – -0.005) | 0.007 | -0.002  (-0.003 – 0.001) | 0.004 | -0.002  (-0.003 – 0.0005) | 0.005 | -0.002  (-0.003 – 0.0003) | 0.01 | -0.002  (-0.003 – 0.001) | 0.001 |
| **Current health expenditure** |  |  |  |  | -1.37  (-4.08 – 1.34) | 0.32 | -1.72  (-4.14 – 0.712) | 0.16 | -1.57  (-4.23 – 1.10) | 0.24 | -2.29  (-4.70 – 0.116) | 0.06 |
| **Control of corruption** |  |  |  |  |  |  | 5.88  (-2.04 – 13.8) | 0.14 | 6.48  (-2.64 – 15.6) | 0.15 | 5.43  (-2.51 – 13.4) | 0.18 |
| **Absence of political instability and violence** |  |  |  |  |  |  |  |  | -1.31  (-9.96 – 7.34) | 0.76 |  |  |
| **Trade** |  |  |  |  |  |  |  |  |  |  | -0.117  (-0.242 – 0.009) | 0.07 |
| **AIC** | 347.68 | | 309.97 | | 265.65 | | 260.06 | | 261.59 | | 252.32 | |
| **BIC** | 349.98 | | 314.52 | | 272.26 | | 268.88 | | 272.62 | | 263.27 | |

**final model

**Table B7.6 Fixed-effect regressions of breast cancer screening on the poverty headcount ratio at $5.50-per-day.**

|  | **Baseline model** | | **+ GDP** | | **+ Current health expenditure** | | **+ Control of corruption** | | **+ Absence of political instability and violence** | | **+ Trade**** | |
| --- | --- | --- | --- | --- | --- | --- | --- | --- | --- | --- | --- | --- |
|  | Coefficient (95% CI) | p-value | Coefficient (95% CI) | p-value | Coefficient (95% CI) | p-value | Coefficient (95% CI) | p-value | Coefficient (95% CI) | p-value | Coefficient (95% CI) | p-value |
| **Breast cancer screening** | -0.215  (-0.419 - -0.011) | 0.04 | 0.011  (-0.338 – 0.360) | 0.95 | -0.045  (-0.246 – 0.155) | 0.65 | 0.035  (-0.162 – 0.232) | 0.72 | 0.054  (-0.140 – 0.247) | 0.58 | 0.080  (-0.105 – 0.266) | 0.39 |
| **GDP per capita** |  |  | -0.003  (-0.004 - -0.002) | <0.001 | -0.003  (-0.004 – -0.002) | <0.001 | -0.003  (-0.004 – -0.002) | <0.001 | -0.003  (-0.004 – -0.002) | <0.001 | -0.003  (-0.004 – -0.002) | <0.001 |
| **Current health expenditure** |  |  |  |  | 1.19  (-4.78 – 2.40) | 0.51 | -1.61  (-4.82 – 1.59) | 0.32 | -1.97  (-5.05 – 1.11) | 0.21 | -2.65  (-5.32 – 0.013) | 0.05 |
| **Control of corruption** |  |  |  |  |  |  | 7.17  (-0.269 – 14.6) | 0.06 | 5.72  (-4.10 – 15.5) | 0.25 | 6.36  (-1.99 – 14.7) | 0.13 |
| **Absence of political instability and violence** |  |  |  |  |  |  |  |  | 3.14  (8.96 – 15.3) | 0.60 |  |  |
| **Trade** |  |  |  |  |  |  |  |  |  |  | -0.211  (-0.381 – -0.040) | 0.02 |
| **AIC** | 417.17 | | 359.78 | | 292.72 | | 287.20 | | 287.38 | | 269.81 | |
| **BIC** | 419.48 | | 364.33 | | 299.34 | | 296.02 | | 298.40 | | 280.76 | |

**final model

# Tables B8: Fixed-effect regressions of cervical cancer screening and on poverty

**Table B8.1 Fixed-effect regressions of cervical cancer screening on the poverty gap at $1.90-per-day.**

|  | **Baseline model** | | **+ GDP** | | **+ Current health expenditure** | | **+ Control of corruption** | | **+ Absence of political instability and violence** | | **+ Trade**** | |
| --- | --- | --- | --- | --- | --- | --- | --- | --- | --- | --- | --- | --- |
|  | Coefficient (95% CI) | p-value | Coefficient (95% CI) | p-value | Coefficient (95% CI) | p-value | Coefficient (95% CI) | p-value | Coefficient (95% CI) | p-value | Coefficient (95% CI) | p-value |
| **Cervical cancer screening** | -0.092  (-0.132 – 0.052) | <0.001 | 0.002  (-0.070 – 0.075) | 0.95 | 0.042  (-0.040 – -0.0002) | 0.31 | 0.048  (-0.039 – 0.135) | 0.28 | 0.027  (-0.052 – 0.106) | 0.50 | 0.026  (-0.054 – 0.106) | 0.52 |
| **GDP per capita** |  |  | -0.001  (-0.001 – -0.0003) | 0.002 | -0.001  (-0.001 – -0.0002) | 0.007 | -0.001  (-0.001 – -0.0002) | 0.006 | -0.001  (-0.002 – -0.001) | <0.001 | -0.001  (-0.002 – -0.0003) | 0.005 |
| **Current health expenditure** |  |  |  |  | -1.55  (-3.62 – 0.519) | 0.14 | -1.55  (-3.63 – 0.523) | 0.14 | -0.995  (-2.59 – 0.595) | 0.22 | -0.989  (-2.61 – 0.629) | 0.23 |
| **Control of corruption** |  |  |  |  |  |  | 0.712  (-2.85 – 4.27) | 0.69 |  |  |  |  |
| **Absence of political instability and violence** |  |  |  |  |  |  |  |  | 3.38  (0.559 – 6.19) | 0.02 | 2.11  (-1.02 – 5.24) | 0.18 |
| **Trade** |  |  |  |  |  |  |  |  |  |  | -0.057  (-0.121 – 0.007) | 0.08 |
| **AIC** | 416.77 | | 387.12 | | 324.13 | | 325.84 | | 308.42 | | 297.91 | |
| **BIC** | 419.47 | | 392.47 | | 331.88 | | 336.18 | | 318.76 | | 310.79 | |

**final model

**Table B8.2 Fixed-effect regressions of cervical cancer screening on the poverty gap at $3.20-per-day.**

|  | **Baseline model** | | **+ GDP** | | **+ Current health expenditure** | | **+ Control of corruption** | | **+ Absence of political instability and violence** | | **+ Trade**** | |
| --- | --- | --- | --- | --- | --- | --- | --- | --- | --- | --- | --- | --- |
|  | Coefficient (95% CI) | p-value | Coefficient (95% CI) | p-value | Coefficient (95% CI) | p-value | Coefficient (95% CI) | p-value | Coefficient (95% CI) | p-value | Coefficient (95% CI) | p-value |
| **Cervical cancer screening** | -0.195  (-0.263 – -0.127) | <0.001 | 0.019  (-0.098 – 0.135) | 0.75 | 0.060  (-0.064 – 0.185) | 0.34 | 0.060  (-0.061 – 0.181) | 0.33 | 0.028  (-0.076 – 0.133) | 0.59 | 0.025  (-0.090 – 0.141) | 0.66 |
| **GDP per capita** |  |  | -0.002  (-0.003 - -0.001) | 0.001 | -0.002  (-0.003 - -0.001) | 0.001 | -0.002  (-0.003 - -0.001) | <0.001 | -0.003  (-0.004 - -0.001) | <0.001 | -0.002  (-0.003 - -0.001) | 0.001 |
| **Current health expenditure** |  |  |  |  | -1.51  (-3.98 – 0.962) | 0.23 | -1.51  (-3.97 – 0.961) | 0.23 | -0.338  (-1.87 – 1.20) | 0.66 | -0.381  (-2.04 – 1.28) | 0.65 |
| **Control of corruption** |  |  |  |  |  |  | -0.043  (-8.46 - 8.37) | 0.99 |  |  |  |  |
| **Absence of political instability and violence** |  |  |  |  |  |  |  |  | 7.10  (1.07 – 13.1) | 0.02 | 4.22  (-1.04 – 9.48) | 0.11 |
| **Trade** |  |  |  |  |  |  |  |  |  |  | -0.133  (-0.293 – 0.027) | 0.10 |
| **AIC** | 539.81 | | 489.09 | | 438.79 | | 440.79 | | 415.53 | | 391.42 | |
| **BIC** | 542.51 | | 494.44 | | 446.55 | | 451.13 | | 425.87 | | 404.30 | |

**final model

**Table B8.3 Fixed-effect regressions of cervical cancer screening on the poverty gap at $5.50-per-day.**

|  | **Baseline model** | | **+ GDP** | | **+ Current health expenditure** | | **+ Control of corruption** | | **+ Absence of political instability and violence** | | **+ Trade**** | |
| --- | --- | --- | --- | --- | --- | --- | --- | --- | --- | --- | --- | --- |
|  | Coefficient (95% CI) | p-value | Coefficient (95% CI) | p-value | Coefficient (95% CI) | p-value | Coefficient (95% CI) | p-value | Coefficient (95% CI) | p-value | Coefficient (95% CI) | p-value |
| **Cervical cancer screening** | -0.353  (-0.451 – -0.256) | <0.001 | -0.005  (-0.154 – 0.145) | 0.95 | 0.029  (-0.141 – 0.199) | 0.73 | 0.021  (-0.149 – 0.191) | 0.81 | -0.015  (-0.131 – 0.100) | 0.78 | -0.019  (-0.150 – 0.112) | 0.78 |
| **GDP per capita** |  |  | -0.003  (-0.004 - -0.002) | <0.001 | -0.003  (-0.004 - -0.002) | <0.001 | -0.003  (-0.004 - -0.002) | <0.001 | -0.004  (-0.007 - -0.002) | <0.001 | -0.004  (-0.005 - -0.002) | <0.001 |
| **Current health expenditure** |  |  |  |  | -1.45  (-4.38 – 1.48) | 0.33 | -1.45  (-4.35 – 1.46) | 0.32 | 0.177  (-1.61 – 1.96) | 0.84 | -0.219  (-2.00 – 2.44) | 0.84 |
| **Control of corruption** |  |  |  |  |  |  | -0.964  (-13.7 – 11.8) | 0.88 |  |  |  |  |
| **Absence of political instability and violence** |  |  |  |  |  |  |  |  | 9.89  (1.34 – 18.5) | 0.02 | 5.26  (-0.457 -10.9) | 0.07 |
| **Trade** |  |  |  |  |  |  |  |  |  |  | -0.208  (0.459 – 0.043) | 0.10 |
| **AIC** | 619.86 | | 551.13 | | 499.61 | | 501.5 | | 475.06 | | 446.01 | |
| **BIC** | 622.56 | | 556.48 | | 507.37 | | 511.9 | | 485.40 | | 458.89 | |

**final model

**Table B8.4 Fixed-effect regressions of cervical cancer screening on the poverty headcount ratio at $1.90-per-day.**

|  | **Baseline model** | | **+ GDP** | | **+ Current health expenditure** | | **+ Control of corruption** | | **+ Absence of political instability and violence** | | **+ Trade**** | |
| --- | --- | --- | --- | --- | --- | --- | --- | --- | --- | --- | --- | --- |
|  | Coefficient (95% CI) | p-value | Coefficient (95% CI) | p-value | Coefficient (95% CI) | p-value | Coefficient (95% CI) | p-value | Coefficient (95% CI) | p-value | Coefficient (95% CI) | p-value |
| **Cervical cancer screening** | -0.234  (-0.328 - -0.140) | <0.001 | 0.033  (-0.127 – 0.194) | 0.68 | 0.084  (-0.089 – 0.257) | 0.34 | 0.085  (-0.084 – 0.254) | 0.32 | 0.041  (-0.108 – 0.189) | 0.59 | 0.036  (-0.128 -0.199) | 0.66 |
| **GDP per capita** |  |  | -0.002  (-0.004 – -0.001) | 0.001 | -0.002  (-0.004 – -0.001) | 0.003 | -0.002  (-0.004 – -0.001) | 0.002 | 0.041  (-0.005 – -0.002) | <0.001 | -0.003  (-0.004 – -0.001) | 0.003 |
| **Current health expenditure** |  |  |  |  | -1.84  -5.18 – 1.50 | 0.27 | -1.84  (-5.18 – 1.49) | 0.27 | -0.250  (-2.43 – 1.93) | 0.82 | -0.366  (-2.70 – 1.97) | 0.75 |
| **Control of corruption** |  |  |  |  |  |  | 0.164  (-11.3 – 11.6) | 0.98 |  |  |  |  |
| **Absence of political instability and violence** |  |  |  |  |  |  |  |  | 9.68  (1.23 – 18.1) | 0.03 | 6.04  (-1.60 – 13.7) | 0.12 |
| **Trade** |  |  |  |  |  |  |  |  |  |  | -0.171  (-0.391 – 0.491) | 0.13 |
| **AIC** | 600.52 | | 552.58 | | 500.93 | | 502.93 | | 478.11 | | 453.13 | |
| **BIC** | 603.22 | | 557.93 | | 508.69 | | 513.27 | | 488.45 | | 467.00 | |

**final model

**Table B8.5 Fixed-effect regressions of cervical cancer screening on the poverty headcount ratio at $3.20-per-day**

|  | **Baseline model** | | **+ GDP** | | **+ Current health expenditure** | | **+ Control of corruption** | | **+ Absence of political instability and violence** | | **+ Trade**** | |
| --- | --- | --- | --- | --- | --- | --- | --- | --- | --- | --- | --- | --- |
|  | Coefficient (95% CI) | p-value | Coefficient (95% CI) | p-value | Coefficient (95% CI) | p-value | Coefficient (95% CI) | p-value | Coefficient (95% CI) | p-value | Coefficient (95% CI) | p-value |
| **Cervical cancer screening** | -0.449  (-0.587 – -0.311) | <0.001 | 0.031  (-0.177 – 0.239) | 0.76 | 0.065  (-0.171 – 0.302) | 0.58 | 0.049  (0.175 – 0.272) | 0.66 | 0.041  (-0.159 – 0.168) | 0.96 | -0.002  (-0.197 – 0.194) | 0.98 |
| **GDP per capita** |  |  | -0.004  (-0.006 – -0.002) | <0.001 | -0.004  (-0.006 – -0.002) | <0.001 | -0.004  (-0.006 – -0.002) | <0.001 | -0.006  (-0.009 – -0.003) | <0.001 | -0.005  (-0.007 – -0.002) | <0.001 |
| **Current health expenditure** |  |  |  |  | -1.27  (-5.59 – 3.05) | 0.56 | -1.25  (-5.53 – 3.01) | 0.56 | 0.990  (-1.93 - 3.91) | 0.50 | -0.985  (-2.62 – 4.59) | 0.58 |
| **Control of corruption** |  |  |  |  |  |  | -2.01  (-20.6 - 16.6) | 0.83 |  |  |  |  |
| **Absence of political instability and violence** |  |  |  |  |  |  |  |  | 13.7  (0.216 – 27.2) | 0.05 | 7.26  (-2.29 – 16.8) | 0.13 |
| **Trade** |  |  |  |  |  |  |  |  |  |  | -0.293  (-0.695 – 0.110) | 0.15 |
| **AIC** | 698.23 | | 632.96 | | 579.77 | | 581.61 | | 559.81 | | 533.73 | |
| **BIC** | 700.93 | | 638.30 | | 587.53 | | 591.94 | | 570.15 | | 546.60 | |

**final model

**Table B8.6 Fixed-effect regressions of cervical cancer screening on the poverty headcount ratio at $5.50-per-day**

|  | **Baseline model** | | **+ GDP** | | **+ Current health expenditure** | | **+ Control of corruption** | | **+ Absence of political instability and violence** | | **+ Trade**** | |
| --- | --- | --- | --- | --- | --- | --- | --- | --- | --- | --- | --- | --- |
|  | Coefficient (95% CI) | p-value | Coefficient (95% CI) | p-value | Coefficient (95% CI) | p-value | Coefficient (95% CI) | p-value | Coefficient (95% CI) | p-value | Coefficient (95% CI) | p-value |
| **Cervical cancer screening** | -0.660  (-0.820 - -0.501) | <0.001 | -0.104  (-0.334 – 0.127) | 0.37 | -0.083  (-0.387 – 0.221) | 0.59 | -0.106  (-0.458 – 0.247) | 0.55 | -0.145  (-0.352 – 0.061) | 0.17 | -0.149  (-0.358 – 0.059) | 0.16 |
| **GDP per capita** |  |  | -0.005  (-0.007 – 0.003) | <0.001 | -0.005  (-0.007 – -0.003) | <0.001 | -0.005  (-0.007 – -0.003) | <0.001 | -0.006  (-0.009 – -0.004) | <0.001 | -0.005  (-0.007 – -0.004) | <0.001 |
| **Current health expenditure** |  |  |  |  | -1.40  (-5.34 - 2.53) | 0.48 | -1.39  (-5.26 – 2.48) | 0.47 | 0.893  (-2.16 – 3.95) | 0.56 | 1.15  (-2.58 – 4.89) | 0.54 |
| **Control of corruption** |  |  |  |  |  |  | -2.75  (-22.1 – 16.6) | 0.78 |  |  |  |  |
| **Absence of political instability and violence** |  |  |  |  |  |  |  |  | 13.9  (2.60 – 25.3) | 0.02 | 6.51  (0.876 – 12.1) | 0.02 |
| **Trade** |  |  |  |  |  |  |  |  |  |  | -0.324  (-0.660 – 0.011) | 0.06 |
| **AIC** | 706.03 | | 621.29 | | 561.82 | | 563.44 | | 535.62 | | 500.30 | |
| **BIC** | 708.73 | | 626.54 | | 569.57 | | 573.78 | | 545.96 | | 513.17 | |

**final model
