# Supplementary material for: Assessing the relationship between coverage of essential health services and poverty levels in low- and middle-income countries
Source: Health Policy Plan. 2024 Feb 1;39(2):156–67. doi: 10.1093/heapol/czae002 (PMC10883664; doi:10.1093/heapol/czae002)
Supplement: czae002_Supp [file czae002_supp.zip › suppl_data/Appendix III - Hausman test amended.docx]

## **Appendix III – Assessing consistency of final models for complete case analyses with the Hausman test**

***Table C1. Results of the Hausman test on final models***

|  | **Inpatient Admissions** | | | | | **Skilled Birth Attendance** | | | | | **Diarrhoea treatment for children** | | | | |
| --- | --- | --- | --- | --- | --- | --- | --- | --- | --- | --- | --- | --- | --- | --- | --- |
|  | **Fixed- effects** | | **Random- effects** | | **Hausman** | **Fixed- effects** | | **Random- effects** | | **Hausman** | **Fixed- effects** | | **Random- effects** | | **Hausman** |
|  | Coefficient  (95% CI) | p-value | Coefficient  (95% CI) | p-value | p-value | Coefficient  (95% CI) | p-value | Coefficient  (95% CI) | p-value | p-value | Coefficient  (95% CI) | p-value | Coefficient  (95% CI) | p-value | p-value |
| Poverty gap $1.90 (2011 PPP) | -0.222  (-0.472 – 0.029) | 0.082 | -0.196  (-0.399 – -0.007) | 0.059 | <0.001 | -0.209  (-0.290 – -0.125) | <0.001 | -0.179  ( -0.236 – -0.123) | <0.001 | 0.014 | -0.267  (-0.408 – -0.127) | <0.001 | -0.137  (-0.218 – -0.056) | 0.001 | <0.001 |
| Poverty gap $3.20 (2011 PPP) | -0.588  (-1.07 – -0.109) | 0.017 | -0.543  (-0.945 – -0.141) | 0.008 | 0.004 | -0.264  (-0.374 – -0.155) | <0.001 | -0.259  (-0.330 – -0.187) | <0.001 | 0.110 | -0.335  (-0.514 – -0.156) | <0.001 | -0.185  (-0.289 – -0.081) | 0.001 | <0.001 |
| Poverty gap $5.50 (2011 PPP) | -0.782  (-1.38 – -0.173) | 0.016 | -0.716  (-1.28 – -0.156) | 0.012 | 0.107 | -0.238  (-0.367 – -0.109) | <0.001 | -0.262  (-0.342– -0.182) | <0.001 | 0.628 | -0.326  (-0.573 – -0.079) | 0.010 | -0.168  (-0.283 – -0.054) | 0.004 | 0.001 |
| Poverty headcount ratio $1.90 (2011 PPP) | -0.790  (-1.41 – -0.153) | 0.021 | -0.722  (-1.26 – -0.182) | 0.009 | <0.001 | -0.372  (-0.529 – -0.216) | <0.001 | -0.364  (-0.465 – -0.262) | <0.001 | 0.042 | -0.437  (-0.702 – -0.173) | 0.001 | -0.249  (-0.398 – -0.100) | 0.001 | <0.001 |
| Poverty headcount ratio $3.20 (2011 PPP) | -1.190  (-2.18 – -0.193) | 0.020 | -0.928  (-1.80 – -0.059) | 0.036 | 0.054 | -0.298  (-0.497 – -0.098) | 0.004 | -0.358  (-0.472 – -0.245) | <0.001 | 0.435 | -0.387  (-0.768 – -0.007) | 0.046 | -0.176  (-0.336 – -0.016) | 0.031 | 0.003 |
| Poverty headcount ratio $5.50 (2011 PPP) | -0.922  (-1.69 – -0.152) | 0.020 | -0.871  (-1.56 – -0.181) | 0.013 | 0.897 | -0.136  (-0.258 – -0.015) | 0.029 | -0.203  (-0.302 – -0.103) | <0.001 | 0.108 | -0.244  (-0.599 – 0.110) | 0.175 | -0.102  (-0.237 – 0.033) | 0.139 | 0.006 |

Note: These fixed-effects regressions do not represent the ones tested in the Hausman test, but the ones presented in the complete case analyses, which use robust standard errors. Both have the same coefficients, but CIs and p-values vary slightly

|  | **Acute respiratory infection treatment** | | | | | **Antenatal care utilization** | | | |  | **Full immunization** | | | | |
| --- | --- | --- | --- | --- | --- | --- | --- | --- | --- | --- | --- | --- | --- | --- | --- |
|  | **Fixed- effects** | | **Random- effects** | | **Hausman** | **Fixed- effects** | | **Random- effects** | | **Hausman** | **Fixed- effects** | | **Random- effects** | | **Hausman** |
|  | Coefficient  (95% CI) | p-value | Coefficient  (95% CI) | p-value | p-value | Coefficient | p-value | Coefficient | p-value | p-value | Coefficient | p-value | Coefficient | p-value | p-value |
| Poverty gap $1.90 (2011 PPP) | -0.108  (-0.296 – 0.081) | 0.259 | -0.101  (-0.197 – -0.004) | 0.040 | 0.439 | -0.231  (-0.330 – -0.131) | <0.001 | -0.184  (-0.254 – -0.114) | <0.001 | 0.019 | -0.191  (-0.323 - -0.060) | 0.005 | -0.140  (-0.204 – -0.076) | <0.001 | 0.008 |
| Poverty gap $3.20 (2011 PPP) | -0.124  (-0.314 – 0.066) | 0.198 | -0.137  (-0.256 – -0.017) | 0.025 | 0.748 | -0.357  (-0.494 – -0.221) | <0.001 | -0.314  (-0.404 – -0.224) | <0.001 | 0.363 | -0.229  (-0.381 - -0.077) | 0.004 | -0.197  (-0.280 – -0.114) | <0.001 | 0.142 |
| Poverty gap $5.50 (2011 PPP) | -0.088  (-0.244 - 0.068) | 0.267 | -0.122  (-0.246 – 0.003) | 0.055 | 0.911 | -0.411  (-0.592 – -0.229) | <0.001 | -0.375  (-0.472 – -0.279) | <0.001 | 0.823 | -0.218  (-0.362 - -0.073) | 0.004 | -0.209  (-0.301 – -0.117) | <0.001 | 0.558 |
| Poverty headcount ratio $1.90 (2011 PPP) | -0.188  (-0.452 – 0.077) | 0.162 | -0.207  (-0.378 – -0.037) | 0.017 | 0.662 | -0.483  (-0.6492 – -0.276) | <0.001 | -0.423  (-0.557 – -0.289) | <0.001 | 0.351 | -0.316  (-0.524 - -0.109) | 0.003 | -0.263  (-0.382 – -0.144) | <0.001 | 0.015 |
| Poverty headcount ratio $3.20 (2011 PPP) | -0.101  (-0.282 – 0.079) | 0.267 | -0.149  (-0.319 – 0.020) | 0.084 | 0.907 | -0.559  (-0.848 – -0.271) | <0.001 | -0.514  (-0.651 – -0.378) | <0.001 | 0.922 | -0.255  (-0.452 - -0.059) | 0.011 | -0.268  (-0.397 – -0.139) | <0.001 | 0.615 |
| Poverty headcount ratio $5.50 (2011 PPP) | 0.019  (-0.106 – 0.145) | 0.755 | -0.053  (-0.194 – 0.088) | 0.459 | 0.052 | -0.381  (-0.642 – -0.119) | 0.005 | -0.377  (-0.499 – -0.254) | <0.001 | 0.624 | -0.149  (-0.289 - -0.009) | 0.037 | -0.182  (-0.292 – -0.072) | 0.001 | 0.463 |

***Table C1. [continued]***

**Table C1 [end]**

|  | Breast cancer screening | | | | | Cervical cancer screening | | | | |
| --- | --- | --- | --- | --- | --- | --- | --- | --- | --- | --- |
|  | Fixed- effects | | Random- effects | | Hausman | Fixed- effects | | Random- effects | | Hausman |
|  | Coefficient  (95% CI) | p-value | Coefficient  (95% CI) | p-value | p-value | Coefficient | p-value | Coefficient | p-value | p-value |
| Poverty gap $1.90 (2011 PPP) | -0.015  (-0.041 – 0.012) | 0.264 | -0.032  (-0.096 – 0.032) | 0.323 | 0.178 | 0.026  (-0.054 – 0.106) | 0.521 | -0.011  (-0.063 – 0.041) | 0.677 | 0.585 |
| Poverty gap $3.20 (2011 PPP) | 0.004  (-0.078 - 0.086) | 0.922 | -0.043  (-0.155 – 0.069) | 0.449 | 0.043 | 0.025  (-0.090 – 0.141) | 0.663 | -0.051  (-0.159 – 0.058) | 0.298 | 0.106 |
| Poverty gap $5.50 (2011 PPP) | 0.027  (-0.084 - 0.138) | 0.623 | -0.034  (-0.172 – -0.104) | 0.629 | 0.082 | -0.019  (-0.150 – 0.112) | 0.775 | -0.048  (-0.183 – 0.086) | 0.069 | 0.192 |
| Poverty headcount ratio $1.90 (2011 PPP) | 0.008  (-0.099 – 0.115) | 0.875 | -0.061  (-0.217 – 0.091) | 0.434 | 0.031 | 0.036  (-0.128 -0.199) | 0.662 | -0.057  (-0.175 – 0.061) | 0.342 | 0.787 |
| Poverty headcount ratio $3.20 (2011 PPP) | 0.023  (-0.133 - 0.178) | 0.771 | -0.053  (-0.240 – 0.135) | 0.582 | 0.028 | -0.002  (-0.197 – 0.194) | 0.984 | -0.129  (-0.283 – 0.026) | 0.081 | 0.477 |
| Poverty headcount ratio $5.50 (2011 PPP) | 0.080  (-0.105 - 0.266) | 0.388 | 0.0004  (-0.190 – 0.191) | 0.997 | 0.583 | -0.149  (-0.358 – 0.059) | 0.157 | -0.184  (-0.315 - -0.054) | 0.006 | 0.178 |
