# Supplementary material for: Assessing the relationship between coverage of essential health services and poverty levels in low- and middle-income countries
Source: Health Policy Plan. 2024 Feb 1;39(2):156–67. doi: 10.1093/heapol/czae002 (PMC10883664; doi:10.1093/heapol/czae002)
Supplement: czae002_Supp [file czae002_supp.zip › suppl_data/Appendix IV - list of countries amended.docx]

**Appendix IV – List of 96 countries tested in complete case analyses**

| Country | Year | Country | Year | Country | Year | Country | Year |
| --- | --- | --- | --- | --- | --- | --- | --- |
| Angola | 2001 | Dominican Republic | 2013 | Lao PDR | 2000 | Russian Federation | 2012 |
| Angola | 2015 | Dominican Republic | 2014 | Lao PDR | 2003 | Russian Federation | 2013 |
| Albania | 2000 | Ecuador | 2003 | Lao PDR | 2006 | Russian Federation | 2014 |
| Albania | 2002 | Ecuador | 2004 | Lao PDR | 2011 | Rwanda | 2000 |
| Albania | 2005 | Egypt, Arab Rep. | 1995 | Lao PDR | 2017 | Rwanda | 2005 |
| Albania | 2008 | Egypt, Arab Rep. | 2000 | Liberia | 2006 | Rwanda | 2007 |
| Albania | 2012 | Egypt, Arab Rep. | 2003 | Liberia | 2013 | Rwanda | 2010 |
| Albania | 2017 | Egypt, Arab Rep. | 2005 | Liberia | 2014 | Rwanda | 2014 |
| Argentina | 2003 | Egypt, Arab Rep. | 2008 | Sri Lanka | 2003 | Sudan | 2000 |
| Argentina | 2005 | Egypt, Arab Rep. | 2014 | Sri Lanka | 2015 | Sudan | 2010 |
| Argentina | 2009 | Egypt, Arab Rep. | 2015 | Sri Lanka | 2016 | Sudan | 2014 |
| Armenia | 2000 | Ethiopia | 2000 | Lesotho | 2000 | Senegal | 1997 |
| Armenia | 2001 | Ethiopia | 2003 | Lesotho | 2004 | Senegal | 2000 |
| Armenia | 2002 | Ethiopia | 2005 | Lesotho | 2009 | Senegal | 2003 |
| Armenia | 2003 | Ethiopia | 2011 | Lesotho | 2014 | Senegal | 2005 |
| Armenia | 2004 | Ethiopia | 2015 | Morocco | 1992 | Senegal | 2010 |
| Armenia | 2005 | Ethiopia | 2016 | Morocco | 2000 | Senegal | 2012 |
| Armenia | 2006 | Gabon | 2000 | Morocco | 2003 | Senegal | 2014 |
| Armenia | 2007 | Gabon | 2012 | Moldova | 2000 | Senegal | 2015 |
| Armenia | 2008 | Georgia | 2000 | Moldova | 2005 | Senegal | 2016 |
| Armenia | 2009 | Georgia | 2003 | Moldova | 2011 | Senegal | 2017 |
| Armenia | 2010 | Georgia | 2005 | Moldova | 2012 | Sierra Leone | 2000 |
| Armenia | 2011 | Georgia | 2011 | Moldova | 2013 | Sierra Leone | 2005 |
| Armenia | 2012 | Georgia | 2016 | Madagascar | 1997 | Sierra Leone | 2008 |
| Armenia | 2013 | Ghana | 1993 | Madagascar | 2000 | Sierra Leone | 2010 |
| Armenia | 2015 | Ghana | 1998 | Madagascar | 2001 | Sierra Leone | 2011 |
| Armenia | 2016 | Ghana | 2003 | Madagascar | 2003 | Sierra Leone | 2013 |
| Burundi | 2000 | Ghana | 2005 | Madagascar | 2005 | Sierra Leone | 2017 |
| Burundi | 2005 | Ghana | 2006 | Madagascar | 2008 | El Salvador | 1998 |
| Burundi | 2006 | Ghana | 2007 | Maldives | 2009 | El Salvador | 2003 |
| Burundi | 2010 | Ghana | 2008 | Maldives | 2016 | El Salvador | 2008 |
| Burundi | 2016 | Ghana | 2011 | Mexico | 2000 | El Salvador | 2014 |
| Benin | 1996 | Ghana | 2012 | Mexico | 2001 | Serbia | 2002 |
| Benin | 2001 | Ghana | 2014 | Mexico | 2002 | Serbia | 2003 |
| Benin | 2006 | Guinea | 1999 | Mexico | 2006 | Serbia | 2005 |
| Benin | 2011 | Guinea | 2005 | Mexico | 2012 | Serbia | 2007 |
| Benin | 2014 | Guinea | 2012 | Mexico | 2015 | Serbia | 2010 |
| Benin | 2015 | Guinea | 2016 | North Macedonia | 2005 | Serbia | 2013 |
| Benin | 2017 | Gambia, The | 2000 | North Macedonia | 2011 | Serbia | 2014 |
| Burkina Faso | 1992 | Gambia, The | 2005 | Mali | 1995 | South Sudan | 2000 |
| Burkina Faso | 1998 | Gambia, The | 2013 | Mali | 2001 | South Sudan | 2010 |
| Burkina Faso | 2002 | Gambia, The | 2015 | Mali | 2003 | Sao Tome and Principe | 2000 |
| Burkina Faso | 2003 | Guinea-Bissau | 2000 | Mali | 2006 | Sao Tome and Principe | 2008 |
| Burkina Faso | 2006 | Guinea-Bissau | 2006 | Mali | 2009 | Sao Tome and Principe | 2014 |
| Burkina Faso | 2010 | Guinea-Bissau | 2014 | Mali | 2012 | Eswatini | 2000 |
| Burkina Faso | 2013 | Guatemala | 1995 | Mali | 2015 | Eswatini | 2003 |
| Bangladesh | 1993 | Guatemala | 1998 | Myanmar | 2000 | Eswatini | 2006 |
| Bangladesh | 1996 | Guatemala | 2000 | Myanmar | 2003 | Eswatini | 2010 |
| Bangladesh | 1999 | Guatemala | 2002 | Myanmar | 2009 | Eswatini | 2014 |
| Bangladesh | 2003 | Guatemala | 2003 | Myanmar | 2014 | Chad | 1996 |
| Bangladesh | 2004 | Guatemala | 2006 | Myanmar | 2015 | Chad | 2000 |
| Bangladesh | 2006 | Guatemala | 2011 | Montenegro | 2005 | Chad | 2003 |
| Bangladesh | 2007 | Guatemala | 2014 | Montenegro | 2013 | Chad | 2004 |
| Bangladesh | 2011 | Honduras | 2005 | Mongolia | 2000 | Chad | 2010 |
| Bangladesh | 2012 | Honduras | 2011 | Mongolia | 2002 | Chad | 2014 |
| Bangladesh | 2014 | Indonesia | 1997 | Mongolia | 2005 | Togo | 1998 |
| Bangladesh | 2016 | Indonesia | 2000 | Mongolia | 2007 | Togo | 2000 |
| Bulgaria | 2000 | Indonesia | 2001 | Mongolia | 2009 | Togo | 2006 |
| Bulgaria | 2003 | Indonesia | 2002 | Mongolia | 2010 | Togo | 2010 |
| Bulgaria | 2006 | Indonesia | 2004 | Mongolia | 2013 | Togo | 2013 |
| Bulgaria | 2007 | Indonesia | 2005 | Mozambique | 1997 | Thailand | 2003 |
| Bulgaria | 2008 | Indonesia | 2006 | Mozambique | 2003 | Thailand | 2005 |
| Bulgaria | 2014 | Indonesia | 2007 | Mozambique | 2008 | Thailand | 2008 |
| Bosnia and Herzegovina | 2000 | Indonesia | 2008 | Mozambique | 2011 | Thailand | 2009 |
| Bosnia and Herzegovina | 2001 | Indonesia | 2009 | Mauritania | 2003 | Thailand | 2011 |
| Bosnia and Herzegovina | 2003 | Indonesia | 2010 | Mauritania | 2007 | Thailand | 2012 |
| Bosnia and Herzegovina | 2006 | Indonesia | 2011 | Mauritania | 2011 | Thailand | 2013 |
| Bosnia and Herzegovina | 2011 | Indonesia | 2012 | Mauritania | 2015 | Thailand | 2015 |
| Belarus | 2005 | Indonesia | 2013 | Malawi | 1992 | Tajikistan | 1999 |
| Belarus | 2012 | Indonesia | 2014 | Malawi | 2000 | Tajikistan | 2000 |
| Belarus | 2016 | Indonesia | 2015 | Malawi | 2003 | Tajikistan | 2003 |
| Bolivia | 1993 | India | 1992 | Malawi | 2004 | Tajikistan | 2005 |
| Bolivia | 1998 | India | 1998 | Malawi | 2006 | Tajikistan | 2007 |
| Bolivia | 2000 | India | 2003 | Malawi | 2010 | Tajikistan | 2009 |
| Bolivia | 2003 | India | 2005 | Malawi | 2013 | Tajikistan | 2011 |
| Bolivia | 2008 | India | 2007 | Malawi | 2015 | Tajikistan | 2012 |
| Brazil | 1996 | India | 2015 | Malawi | 2016 | Tajikistan | 2017 |
| Brazil | 1998 | Iran, Islamic Rep. | 2000 | Namibia | 1992 | Timor-Leste | 2001 |
| Brazil | 2003 | Iran, Islamic Rep. | 2010 | Namibia | 2000 | Timor-Leste | 2007 |
| Brazil | 2006 | Iraq | 2000 | Namibia | 2003 | Timor-Leste | 2009 |
| Brazil | 2008 | Iraq | 2006 | Namibia | 2006 | Timor-Leste | 2014 |
| Bhutan | 2010 | Iraq | 2011 | Namibia | 2013 | Timor-Leste | 2016 |
| Bhutan | 2011 | Iraq | 2015 | Niger | 1998 | Tunisia | 2003 |
| Bhutan | 2014 | Jamaica | 1990 | Niger | 2000 | Tunisia | 2011 |
| China | 2000 | Jamaica | 1991 | Niger | 2006 | Turkey | 1993 |
| China | 2003 | Jamaica | 1992 | Niger | 2011 | Turkey | 1998 |
| China | 2007 | Jamaica | 1993 | Niger | 2012 | Turkey | 2000 |
| China | 2011 | Jamaica | 1994 | Nigeria | 1990 | Turkey | 2003 |
| Cote d'Ivoire | 1994 | Jamaica | 1995 | Nigeria | 2000 | Turkey | 2014 |
| Cote d'Ivoire | 1998 | Jamaica | 1996 | Nigeria | 2003 | Turkey | 2017 |
| Cote d'Ivoire | 2000 | Jamaica | 1997 | Nigeria | 2007 | Tanzania | 1996 |
| Cote d'Ivoire | 2006 | Jamaica | 2002 | Nigeria | 2008 | Tanzania | 1999 |
| Cote d'Ivoire | 2011 | Jamaica | 2003 | Nigeria | 2010 | Tanzania | 2004 |
| Cote d'Ivoire | 2016 | Jamaica | 2004 | Nigeria | 2011 | Tanzania | 2008 |
| Cameroon | 1991 | Jamaica | 2005 | Nigeria | 2012 | Tanzania | 2009 |
| Cameroon | 1998 | Jamaica | 2007 | Nigeria | 2013 | Tanzania | 2010 |
| Cameroon | 2000 | Jamaica | 2008 | Nigeria | 2016 | Tanzania | 2012 |
| Cameroon | 2004 | Jamaica | 2011 | Nicaragua | 1993 | Tanzania | 2014 |
| Cameroon | 2006 | Jordan | 1990 | Nicaragua | 1997 | Tanzania | 2015 |
| Cameroon | 2011 | Jordan | 1997 | Nicaragua | 1998 | Uganda | 1995 |
| Cameroon | 2014 | Jordan | 2000 | Nicaragua | 2001 | Uganda | 2000 |
| Congo, Dem. Rep. | 2001 | Jordan | 2002 | Nicaragua | 2009 | Uganda | 2006 |
| Congo, Dem. Rep. | 2007 | Jordan | 2006 | Nicaragua | 2014 | Uganda | 2011 |
| Congo, Dem. Rep. | 2010 | Jordan | 2007 | Nepal | 1996 | Uganda | 2014 |
| Congo, Dem. Rep. | 2012 | Jordan | 2012 | Nepal | 2001 | Uganda | 2016 |
| Congo, Dem. Rep. | 2013 | Jordan | 2017 | Nepal | 2003 | Ukraine | 2002 |
| Congo, Rep. | 2005 | Kazakhstan | 1995 | Nepal | 2006 | Ukraine | 2005 |
| Congo, Rep. | 2011 | Kazakhstan | 1999 | Nepal | 2011 | Ukraine | 2007 |
| Congo, Rep. | 2014 | Kazakhstan | 2001 | Nepal | 2014 | Ukraine | 2012 |
| Colombia | 1995 | Kazakhstan | 2002 | Nepal | 2016 | Uzbekistan | 1996 |
| Colombia | 2000 | Kazakhstan | 2003 | Pakistan | 1990 | Uzbekistan | 2000 |
| Colombia | 2003 | Kazakhstan | 2006 | Pakistan | 2003 | Uzbekistan | 2002 |
| Colombia | 2004 | Kazakhstan | 2010 | Pakistan | 2006 | Uzbekistan | 2003 |
| Colombia | 2008 | Kazakhstan | 2015 | Pakistan | 2012 | Uzbekistan | 2006 |
| Colombia | 2009 | Kenya | 1993 | Pakistan | 2014 | Uzbekistan | 2011 |
| Colombia | 2010 | Kenya | 1998 | Peru | 1991 | Uzbekistan | 2014 |
| Colombia | 2015 | Kenya | 2000 | Peru | 1996 | Vietnam | 1992 |
| Comoros | 1996 | Kenya | 2003 | Peru | 2000 | Vietnam | 1997 |
| Comoros | 2000 | Kenya | 2004 | Peru | 2003 | Vietnam | 2000 |
| Comoros | 2004 | Kenya | 2005 | Peru | 2006 | Vietnam | 2002 |
| Comoros | 2012 | Kenya | 2008 | Peru | 2007 | Vietnam | 2004 |
| Costa Rica | 1999 | Kenya | 2014 | Peru | 2008 | Vietnam | 2006 |
| Costa Rica | 2006 | Kenya | 2015 | Peru | 2009 | Vietnam | 2008 |
| Costa Rica | 2011 | Kyrgyz Republic | 1997 | Peru | 2010 | Vietnam | 2010 |
| Costa Rica | 2012 | Kyrgyz Republic | 1998 | Peru | 2011 | Vietnam | 2012 |
| Dominican Republic | 1996 | Kyrgyz Republic | 2005 | Peru | 2012 | Vietnam | 2013 |
| Dominican Republic | 1999 | Kyrgyz Republic | 2006 | Peru | 2013 | Vietnam | 2014 |
| Dominican Republic | 2000 | Kyrgyz Republic | 2012 | Peru | 2014 | Vietnam | 2015 |
| Dominican Republic | 2002 | Kyrgyz Republic | 2014 | Peru | 2015 | Kosovo | 2000 |
| Dominican Republic | 2003 |  |  | Peru | 2016 | Kosovo | 2007 |
| Dominican Republic | 2007 |  |  | Philippines | 1993 | Kosovo | 2013 |
|  |  |  |  | Philippines | 1998 | Kosovo | 2016 |
|  |  |  |  | Philippines | 1999 | South Africa | 1998 |
|  |  |  |  | Philippines | 2003 | South Africa | 2002 |
|  |  |  |  | Philippines | 2008 | South Africa | 2007 |
|  |  |  |  | Philippines | 2013 | South Africa | 2016 |
|  |  |  |  | Philippines | 2017 | Zambia | 1996 |
|  |  |  |  | Paraguay | 1990 | Zambia | 1999 |
|  |  |  |  | Paraguay | 2002 | Zambia | 2001 |
|  |  |  |  | Paraguay | 2016 | Zambia | 2003 |
|  |  |  |  | West Bank and Gaza | 2010 | Zambia | 2007 |
|  |  |  |  | West Bank and Gaza | 2014 | Zambia | 2013 |
|  |  |  |  | Russian Federation | 1994 | Zimbabwe | 1994 |
|  |  |  |  | Russian Federation | 1995 | Zimbabwe | 1999 |
|  |  |  |  | Russian Federation | 2000 | Zimbabwe | 2003 |
|  |  |  |  | Russian Federation | 2003 | Zimbabwe | 2005 |
|  |  |  |  | Russian Federation | 2007 | Zimbabwe | 2009 |
|  |  |  |  | Russian Federation | 2010 | Zimbabwe | 2010 |
|  |  |  |  | Russian Federation | 2011 | Zimbabwe | 2014 |
|  |  |  |  |  |  | Zimbabwe | 2015 |
|  |  |  |  |  |  |  |  |
