# Supplementary material for: Assessing the relationship between coverage of essential health services and poverty levels in low- and middle-income countries
Source: Health Policy Plan. 2024 Feb 1;39(2):156–67. doi: 10.1093/heapol/czae002 (PMC10883664; doi:10.1093/heapol/czae002)
Supplement: czae002_Supp [file czae002_supp.zip › suppl_data/Appendix V - sensitivity analyses amended.docx]

|  | Antenatal care utilization | | | Full immunization in children | | | Breast cancer screening | | | | Cervical cancer screening | | |
| --- | --- | --- | --- | --- | --- | --- | --- | --- | --- | --- | --- | --- | --- |
|  | Coefficient  (95% CI) | p-value | Hausman | Coefficient  (95% CI) | p-value | Hausman | Coefficient  (95% CI) | | p-value | Hausman | Coefficient  (95% CI) | p-value | Hausman |
| Poverty gap $1.90 (2011 PPP) | -0.142  (-0.249 – -0.036) | 0.009 | Fixed | -0.151  (-0.258 – -0.043) | 0.006 | Fixed | -0.005  (-0.046 – 0.035) | 0.792 | | Random | -0.035  (-0.077 – 0.007) | 0.100 | Random |
| Poverty gap $3.20 (2011 PPP) | -0.245  (-0.295 – -0.194) | <0.001 | Random | -0.169  (-0.214 – -0.124) | <0.001 | Random | 0.0178  (-0.056 – 0.092) | 0.631 | | Fixed | -0.089  (-0.156 – -0.022) | 0.009 | Random |
| Poverty gap $5.50 (2011 PPP) | -0.272  (-0.328 – -0.217) | <0.001 | Random | -0.163  (-0.214 – -0.112) | <0.001 | Random | 0.008  (-0.084 – 0.100) | 0.863 | | Random | -0.173  (-0.258 – -0.088) | <0.001 | Random |
| Poverty headcount ratio $1.90 (2011 PPP) | -0.344  (-0.413 – -0.275) | <0.001 | Random | -0.234  (-0.392 – -0.075) | 0.004 | Fixed | 0.019  (-0.087 – 0.124) | 0.724 | | Fixed | -0.107  (-0.199 – -0.015) | 0.023 | Random |
| Poverty headcount ratio $3.20 (2011 PPP) | -0.387  (-0.465 – -0.309) | <0.001 | Random | -0.186  (-0.259 – -0.114) | <0.001 | Random | 0.033  (-0.118 – 0.183) | 0.667 | | Fixed | -0.222  (-0.342 – -0.101) | <0.001 | Random |
| Poverty headcount ratio $5.50 (2011 PPP) | -0.229  (-0.299 – -0.159) | <0.001 | Random | -0.123  (-0.185 – -0.062) | <0.001 | Random | 0.057  (-0.075 – 0.189) | 0.394 | | Random | -0.306  (-0.416 – -0.197) | <0.001 | Random |

**Appendix V – Results of sensitivity analyses**

***Table D1. Imputed data: Final models using fixed-effect regressions.***

|  | Inpatient admission | | | Skilled birth attendance | | | Diarrhoea treatment in children | | | Acute respiratory infection treatment | | |
| --- | --- | --- | --- | --- | --- | --- | --- | --- | --- | --- | --- | --- |
|  | Coefficient  (95% CI) | p-value | Hausman | Coefficient  (95% CI) | p-value | Hausman | Coefficient  (95% CI) | p-value | Hausman | Coefficient  (95% CI) | p-value | Hausman |
| Poverty gap $1.90 (2011 PPP) | -0.350  (-0.663 - -0.035) | 0.029 | Fixed | -0.215  (-0.295 – -0.135) | <0.001 | Fixed | -0.165  (-0.283 – -0.046) | 0.007 | Fixed | -0.098  (-0.148 – -0.047) | <0.001 | Random |
| Poverty gap $3.20 (2011 PPP) | -0.685  (-1.22 – -0.145) | 0.014 | Fixed | -0.282  (-0.323 – -0.240) | <0.001 | Random | -0.203  (-0.358 – -0.047) | 0.011 | Fixed | -0.132  (-0.191 – -0.072) | <0.001 | Random |
| Poverty gap $5.50 (2011 PPP) | -0.734  (-1.12 – -0.350) | <0.001 | Random | -0.250  (-0.298 – -0.202) | <0.001 | Random | -0.179  (-0.380 – 0.231) | 0.082 | Fixed | -0.117  (-0.177 – -0.056) | <0.001 | Random |
| Poverty headcount ratio $1.90 (2011 PPP) | -0.880  (-1.62 – -0.135) | 0.021 | Fixed | -0.394  (-0.528 – -0.261) | <0.001 | Fixed | -0.276  (-0.485 – -0.066) | 0.010 | Fixed | -0.192  (-0.274 – -0.110) | <0.001 | Random |
| Poverty headcount ratio $3.20 (2011 PPP) | -1.035  (-1.60 – -0.468) | <0.001 | Random | -0.327  (-0.395 – -0.258) | <0.001 | Random | -0.199  (-0.504 – 0.105) | 0.197 | Fixed | -0.154  (-0.236 – -0.073) | <0.001 | Random |
| Poverty headcount ratio $5.50 (2011 PPP) | -0.627  (-1.12 – -0.137) | 0.012 | Random | -0.107  (-0.170 – -0.044) | 0.001 | Random | -0.103  (-0.367 – 0.162) | 0.442 | Fixed | -0.034  (-0.104 – 0.036) | 0.487 | Fixed |

***Table D1 [continued]***

***Table D2. Final models using fixed-effect regressions only***

|  | **Inpatient admission** | | **Skilled birth attendance** | | **Diarrhoea treatment** | | | **Acute respiratory infection treatment** | | | |
| --- | --- | --- | --- | --- | --- | --- | --- | --- | --- | --- | --- |
|  | Coefficient (95% CI) | p-value | Coefficient  (95% CI) | p-value | Coefficient  (95% CI) | p-value | | Coefficient (95% CI) | | p-value | |
| Poverty gap $1.90 (2011 PPP) | -0.222  (-0.472 – 0.029) | 0.082 | -0.209  (-0.290 – -0.127) | <0.001 | -0.267  (-0.408 – -0.127) | <0.001 | | -0.108  (-0.296 – 0.081) | | 0.259 | |
| Poverty gap $3.20 (2011 PPP) | -0.583  (-1.07 – -0.093) | 0.017 | -0.264  (-0.374 – -0.155) | <0.001 | -0.335  (-0.514 – -0.156) | <0.001 | | -0.124  (-0.314 – 0.066) | | 0.198 | |
| Poverty gap $5.50 (2011 PPP) | -0.777  (-1.38 – -0.173) | 0.016 | -0.238  (-0.367 – -0.109) | <0.001 | -0.326  (-0.573 – -0.079) | 0.010 | | -0.088  (-0.244 - 0.068) | | 0.267 | |
| Poverty headcount ratio $1.90 (2011 PPP) | -0.790  (-1.46 – -0.123) | 0.021 | -0.372  (-0.529 – -0.216) | <0.001 | -0.437  (-0.702 – -0.173) | 0.001 | | -0.188  (-0.452 – 0.077) | | 0.162 | |
| Poverty headcount ratio $3.20 (2011 PPP) | -1.19  (-2.21 – -0.157) | 0.020 | -0.298  (-0.497 – -0.098) | 0.004 | -0.387  (-0.768 – -0.007) | 0.046 | -0.101  (-0.282 – 0.079) | | 0.267 | |  |
| Poverty headcount ratio $5.50 (2011 PPP) | -0.922  (-1.69 – -0.152) | 0.020 | -0.118  (-0.254 – 0.018) | 0.087 | -0.244  (-0.599 – 0.110) | 0.175 | 0.019  (-0.106 – 0.145) | | 0.755 | |  |

***Table D2 [continue]***

|  | **Antenatal care utilization** | | **Full immunization in children** | | **Breast cancer screening** | | **Cervical cancer screening** | |
| --- | --- | --- | --- | --- | --- | --- | --- | --- |
|  | Coefficient  (95% CI) | p-value | Coefficient  (95% CI) | p-value | Coefficient  (95% CI) | p-value | Coefficient (95% CI) | p-value |
| Poverty gap $1.90 (2011 PPP) | -0.231  (-0.330 – -0.131) | <0.001 | -0.191  (-0.323 – -0.060) | 0.005 | -0.015  (-0.041 – 0.012) | 0.264 | 0.026  (-0.054 – 0.106) | 0.521 |
| Poverty gap $3.20 (2011 PPP) | -0.357  (-0.494 – -0.221) | <0.001 | -0.229  (-0.381 – -0.077) | 0.004 | 0.004  (-0.078 – 0.086) | 0.922 | 0.025  (-0.090 – 0.141) | 0.663 |
| Poverty gap $5.50 (2011 PPP) | -0.411  (-0.592 – -0.229) | <0.001 | -0.218  (-0.362 – -0.073) | 0.004 | 0.027  (-0.084 – 0.138) | 0.623 | -0.019  (-0.150 – 0.112) | 0.775 |
| Poverty headcount ratio $1.90 (2011 PPP) | -0.483  (-0.6492 – -0.276) | <0.001 | -0.316  (-0.524 – -0.109) | 0.003 | 0.008  (-0.099 – 0.115) | 0.875 | 0.036  (-0.128 – 0.199) | 0.662 |
| Poverty headcount ratio $3.20 (2011 PPP) | -0.559  (-0.848 – -0.271) | <0.001 | -0.255  (-0.452 – -0.059) | 0.011 | 0.023  (-0.133 – 0.178) | 0.771 | -0.002  (-0.197 – 0.194) | 0.984 |
| Poverty headcount ratio $5.50 (2011 PPP) | -0.381  (-0.642 – -0.119) | 0.005 | -0.149  (-0.289 – -0.009) | 0.037 | 0.080  (-0.105 – 0.266) | 0.388 | -0.149  (-0.358 – 0.059) | 0.157 |
